# Effect of Chirality on the Binding of Viologen Guests in Porphyrin Macrocycles

**DOI:** 10.1002/ejoc.201900221

**Published:** 2019-05-03

**Authors:** Shaji Varghese, Bram Spierenburg, Jeroen P. J. Bruekers, Anne Swartjes, Paul B. White, Johannes A. A. W. Elemans, Roeland J. M. Nolte

**Affiliations:** ^1^ Institute for Molecules and Materials Radboud University Heyendaalseweg 135 6525 AJ Nijmegen The Netherlands

**Keywords:** Host–guest systems, Chirality, Porphyrinoids, Threading, Pseudo‐rotxanes, Cage compounds

## Abstract

As part of a project aimed at the development of chiral processive catalysts that can write information on a polymer chain we describe the synthesis of two optically active porphyrin macrocycles, which are prepared in 3 steps from an achiral precursor compound. Fluorescence and ^1^H‐NMR studies show that one of the macrocycles displays selectivity in the binding of chiral viologen guest molecules.

## Introduction

**Figure 1:**
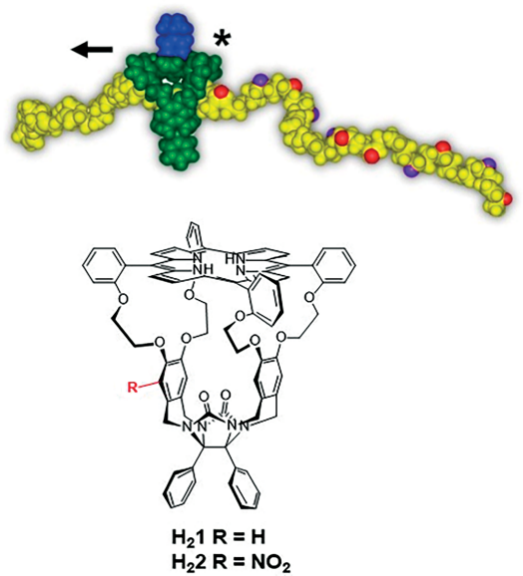
(Top) Chiral porphyrin cage catalyst writing chemical information ((*R*,*R*)‐ and (*S*,*S*)‐epoxides; red and purple balls, respectively) on a polymer chain while moving along it. (Bottom) Chemical structures of achiral porphyrin cage compound **H_2_1** and its chiral derivative (*R_p_*,*S_p_*)‐**H_2_2**.

The work described in this paper is part of a larger project aimed at encoding information into polymers with the help of chiral cage catalysts that thread onto a polymer chain and, while moving along it unidirectionally, write chiral (*R*,*R*)‐ or (*S*,*S*)‐epoxides (digital codes 0 and 1) on this chain (Figure 1)^1^, ^2^, ^3^, ^4^, ^5^ In a previous paper, we reported on the facile, one‐step synthesis of a chiral porphyrin cage compound, i.e. (*R_p_*,*S_p_*)‐**H_2_2** (for notation of chirality, see below) from the achiral derivative **H_2_1**.^6^ The manganese complex of compound **H_2_2** may be an interesting catalyst for the above‐mentioned writing process. As a first step in this direction we report here on the reduction of (*R_p_*,*S_p_*)‐**H_2_2** to its amine, (*R_p_*,*S_p_*)‐**H_2_3**, and the resolution of the Mosher derivatives of this compound into two diastereomers, i.e. compounds (*R*,S_p_^*^)‐**H_2_4** and (*R*,R_p_^*^)‐**H_2_5**. Furthermore, we show that (*R*,S_p_^*^)‐**H_2_4** and (*R*,R_p_^*^)‐**H_2_5** have different affinities for chiral viologen guest molecules. (for recent other chiral cage compounds, see refs.^7^, ^8^, ^9^, ^10^, ^11^, ^12^, ^13^, ^14^, ^15^, ^16^). These experiments will help us decide what chiral polymeric guests should be used in the future threading and epoxidation experiments needed to develop a catalytic machine that can write chiral information on a polymer chain.

## Results and Discussion

Racemic compound (*R_p_*,*S_p_*)‐**H_2_2** was synthesized from **H_2_1** by nitration with HNO_3_ in chloroform at –40 °C (isolated yield 75 %).^6^ Reduction of the nitro function of (*R_p_*,*S_p_*)‐**H_2_2** to its amine was initially tried with palladium on carbon under hydrogen pressure (40 bar) at 40 °C. This resulted in the desired conversion, but also in the reduction of one of the β‐pyrrole double bonds of the porphyrin moiety (as shown by e.g. Maldi‐TOF: *m/z* 1362 instead of 1360). Compound (*R_p_*,*S_p_*)‐**H_2_3** could be obtained, however, by using SnCl_2_ in dioxane/HCl as the reducing agent in a Schlenk bomb.

**Scheme 1:**
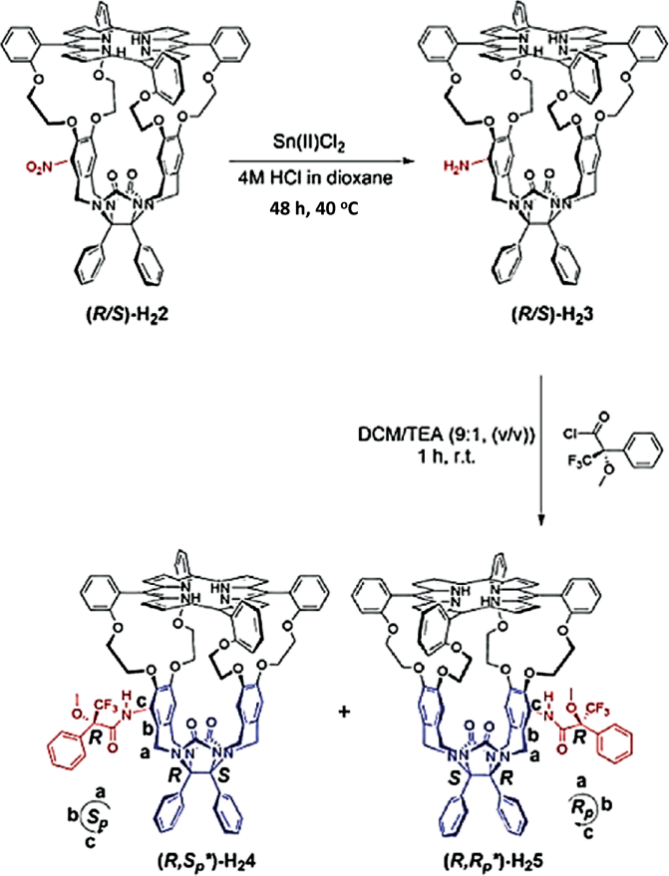
Synthesis of cavity containing porphyrin compounds (*R*,S_p_^*^)‐**H_2_4** and (*R*,R_p_*)‐**H_2_5**. Reaction conditions: (i) Sn(II)Cl_2_, 4 M HCl in 1,4‐dioxane, (ii) (*R*)‐(–)‐α‐methoxy‐α‐(trifluoromethyl)phenylacetyl chloride, chloroform, TEA, 40 % yield over 2 steps.

Reaction of the amine with enantiopure (*R*)‐*(–)*‐α‐methoxy‐α‐(trifluoromethyl)phenylacetyl chloride (Mosher's acid chloride) in dichloromethane using triethylamine (TEA) as a base, readily provided the diastereomeric amides (*R*,S_p_^*^)‐**H_2_4** and (*R*,R_p_^*^)‐**H_2_5** in 40 % yield over two steps (Scheme 1). These diastereomers could be separated by conventional column chromatography (60H silica, eluent CHCl_3_/MeOH). Compounds **H_2_4** and **H_2_5** display planar chirality, for which the notations *S_p_* and *R_p_* can be derived, as indicated in Scheme 1 (see ref.^17^ for nomenclature of compounds displaying planar chirality). In addition to the chirality of the Mosher substituent, which is (*R*), the cage compounds each contain two chiral centers of which the chirality is coupled to the planar chirality of the particular molecule. These chiral centers are also indicated in Scheme 1. Hence, the notations for the two diastereomeric compounds are: (*R*,*S_p_*,*R*,*S*)‐**H_2_4** and (*R*,*R_p_*,*S*,*R*)‐**H_2_5**. Since we do not know which absolute chirality should be assigned to **H_2_4** and **H_2_5** (this requires X‐ray structures of these compounds), we will denote this uncertainty by adding an asterisk: (*R*,S_p_^*^)‐**H_2_4** and (*R*,R_p_^*^)‐**H_2_5**, in which the first letter *R* refers to the chirality of the Mosher substituent and the second letter to the unknown chirality of the porphyrin cage compound.

**Figure 2:**
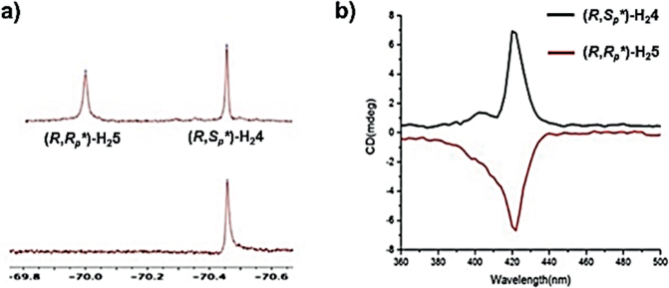
(a) ^19^F‐NMR spectra of the reaction mixture of (*R*,S_p_*)‐**H_2_4** and (*R*,R_p_*)‐**H_2_5** (top) and that of pure (*R*,S_p_*)‐**H_2_4** after separation (bottom). (b) CD spectra of (*R*,S_p_*)‐**H_2_4** and (*R*,R_p_*)‐**H_2_5**.

The structures of the newly synthesized compounds were confirmed by MALDI‐TOF and 1D and 2D NMR spectroscopy. The ^1^H‐NMR spectra of (*R*,S_p_*)‐**H_2_4** and (*R*,R_p_*)‐**H_2_5** showed different peaks as can be expected for diastereomeric compounds. ^19^F‐NMR spectroscopy was used as a tool to check the diastereomeric purity of the compounds. The spectra of the reaction mixture of (*R*,S_p_*)‐**H_2_4** and (*R*,R_p_*)‐**H_2_5** and that of pure (*R*,S_p_*)‐**H_2_4** after separation are shown in Figure 2a as examples. Circular dichroism revealed that the two isolated diastereo‐pure compounds had Cotton effects with opposite sign (Figure 2b).

**Scheme 2:**
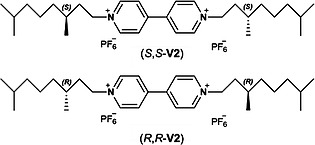
Structures of chiral guest molecules (*S*,*S*)‐ and (*R*,*R*)‐**V2**.

Having the diastereomeric pure hosts (*R*,S_p_*)‐**H_2_4** and (*R*,R_p_*)‐**H_2_5** in hand we investigated whether these compounds could bind the previously studied guest *N*,*N′*‐dimethylviologen dihexafluorophosphate (**V1**) and the chiral guest molecules (*S*,*S*)‐**V2** and (*R*,*R*)‐**V2** (Scheme 2). Furthermore, we were interested to see whether the diastereomeric hosts could discriminate between the latter two chiral guests. To this end, fluorescence titrations were performed in a solvent mixture of acetonitrile and chloroform (1:1, v/v). On binding of the guest, the fluorescence of the porphyrin is quenched, and this process can be followed as a function of the amount of added guest by recording the intensities of the maxima at 653 and 719 nm in the fluorescence spectra. The results are presented in Table 1.

**Table 1:** Association constants and Gibbs free energies of binding for various host–guest complexes^a^

Host	(*R*,S_p_*)‐H_2_4			(*R*,S_p_*)‐H_2_5		
Guest	V1	(*R*,*R*)‐V2	(*S*,*S*)‐V2	V1	(*R*,*R*)‐V2	(*S*,*S*)‐V2
*K*_a_/M^–1^	0.883 × 10^6^	1.388 × 10^6^	3.88 × 10^6^	1.29 × 10^6^	8.0 × 10^6^	7.3 × 10^6^
Error *K*_a_ /M^–1^	0.089 × 10^6^	0.045 × 10^6^	0.26 × 10^6^	0.21 × 10^6^	1.4 × 10^6^	0.37 × 10^6^
ΔG° /kJ mol^–1^	–33.9	–35.0	–37.6	–34.9	–39.4	–39.1

a Measurements were performed in duplicate or triplicate in acetonitrile‐chloroform (1:1. v/v). The errors in the K_a_ values are indicated in the table. The errors in the fitting of the data points are given in the Supporting Information. λ_exc_. 420 nm, λ_meas._ 653 and 719 nm.

**V1** binds in **H_2_1** with a binding constant of *K_a_* = 0.60 × 10^6^ M^–1^ 18 and in (*R*,S_p_*)‐**H_2_4** and (*R*,R_p_*)‐**H_2_5** with binding constants of *K*_*a*_ = 0.88 × 10^6^ and 1.39 × 10^6^ M^–1^, respectively. The somewhat higher affinities of **V1** for the latter two hosts compared to **H_2_1** may be attributed to the additional van der Waals interactions that are possible with the Mosher substituent present on the side‐walls of (*R*,S_p_*)‐**H_2_4** and (*R*,R_p_*)‐**H_2_5**. Table 1 shows that the chiral guests (*S*,*S*)‐**V2** and (*R*,*R*)‐**V2** all bind stronger to the two hosts than** V1**. This must be the result of the longer tails in **V2**, which allow for additional binding interactions with the hosts. Binding of the chiral guests is lower in (*R*,S_p_*)‐**H_2_4** than in (*R*,R_p_*)‐**H_2_5**, suggesting that the substituent in the former diastereomer shields the entrance of the cavity more than the substituent in the latter diastereomer. Interestingly, host (*R*,S_p_*)‐**H_2_4** displays selectivity towards the guest (*S*,*S*)‐**V2** (factor of 2.8), whereas (*R*,R_p_*)‐**H_2_5** does not discriminate between (*S*,*S*)‐**V2** and (*R*,*R*)‐**V2**, within experimental error. The fact that compound (*R*,S_p_*)‐**H_2_4** displays selectivity is promising because the chiral centers in the guests are quite far away from the viologen binding moieties, making that the non‐bonded interactions with the host that lead to chiral discrimination will not be optimal. Guests containing bulkier chiral substituents can be expected to be more efficient and experiments in this direction are underway.

**Scheme 3:**
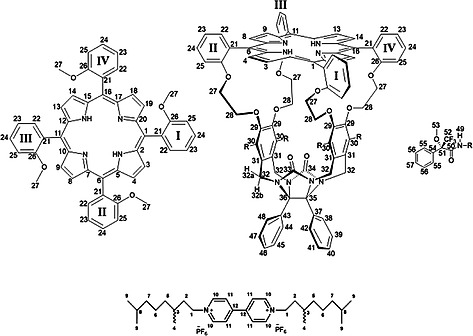
Proton and carbon numbering in host and guest compounds. For protons **27**, **28**, and **32** two different signals are present, but only for the CH_2_‐**32** protons, a distinction was made between the proton pointing upwards (**32a**) and the proton pointing downwards (**32b**).

**Figure 3:**
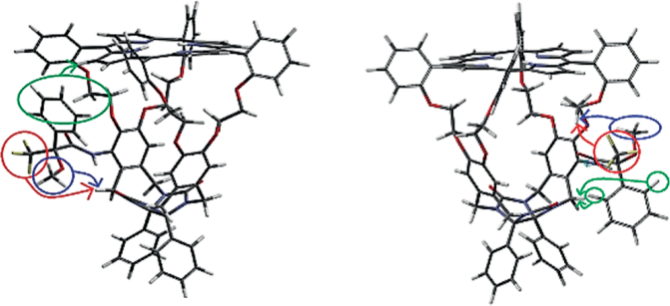
3D calculated models (Spartan '14) of (*R*,S_p_*)‐**H_2_4** (left) and (*R*,R_p_*)‐**H_2_5** (right). The colored circles and arrows indicate nOe contacts observed in the ^1^H‐^19^F and ^1^H‐^1^H correlation spectra, see text for details.

In order to find out what the orientations of the chiral substituents in hosts **H_2_4** and **H_2_5** are and to see to what extent they block the entrance of the cavities, 2D‐NMR experiments were carried out. In compound (*R*,S_p_*)‐**H_2_4**, ^1^H‐^19^F nOe correlations are found between the trifluoromethyl group and the *out*‐proton *32a* (for numbering see Scheme 3) of one of the benzylic methylene groups of the most nearby side‐wall (red arrow in Figure 3 left). Additionally, in the 2D‐ROESY spectrum nOe contacts are present between the methoxy protons of the Mosher amide substituent and the same *out*‐proton of the afore‐mentioned methylene group (blue arrow in Figure 3 left). 3D‐modelling (Spartan '14) revealed that such interactions are only possible when the phenyl group of the Mosher substituent is pointing upwards, i.e. in the direction of the *meso*‐phenyl‐substituted porphyrin ring. In line with this, nOe contacts are observed between the phenyl protons of the Mosher amide group at 7.22 ppm and the peripheral β‐pyrrole protons *3* and *4* of the porphyrin at 8.94 ppm (green arrow in Figure 3, left). Furthermore, an unusual upfield shift from 7.40 to 6.64 ppm was seen for the *ortho*‐proton *25* of the *meso*‐phenyl group of the porphyrin ring and for both upper methylene protons *27* of the oxyethylene spacer (from ca. 4.5–3.9 to 3.7 and 3.4 ppm) on the side to which the Mosher amide group is attached.

Diastereomer (*R*,S_p_*)‐**H_2_5**, showed ^1^H‐^19^F nOe correlations between the trifluoro group of the Mosher amide substituent and one of the protons *27* (4.48 ppm) of the oxyethylene spacer (Figure 3, right, red arrow). Additionally, in the 2D‐ROESY spectrum, nOe contacts were present between the methoxy protons of the Mosher amide group and the same proton *27* of the oxyethylene spacer (Figure 3, right, blue arrow). These observations indicate that the phenyl group of the Mosher amide is oriented downwards, i.e. in the direction of the diphenylglycoluril part of the cage compound. Further evidence for this orientation comes from nOe contacts between the *ortho*‐phenyl protons *55* of the Mosher phenyl group and the *out*‐proton *32a* of the benzylic methylene group of one of the side‐walls (Figure 3, right, green arrow).

The upward orientation of the Mosher phenyl group in (*R*,S_p_*)‐**H_2_4** allows the formation of a hydrogen bond between the Mosher amide proton and the nearby carbonyl group (*33*) of the glycoluril framework of the cage compound. According to the calculations we performed, such a hydrogen bond is not possible for compound (*R*,R_p_*)‐**H_2_5**. The chemical shift of the amide proton in (*R*,S_p_*)‐**H_2_4** is 9.17 ppm and in (*R*,R_p_*)‐**H_2_5** 8.13 ppm. The hydrogen bond in the former compound is expected to have a length of circa 2.5 Å,^19^ resulting in a 1 ppm downfield shift compared to the non‐hydrogen bonded amide proton in the latter compound. For comparison we also measured the chemical shift of the amide proton in the model compound (*R*)‐*N*‐(2,6‐dimethylphenyl)‐3,3,3‐trifluoro‐2‐methoxy‐2‐phenylpropanamide (**5**), which in diluted form cannot form a hydrogen bond. It was found to be located at 8.17 ppm, which is similar to the shift value in (*R*,R_p_*)‐**H_2_5**. Based on the results above in combination with the calculated structures of the two compounds (see Figure 3) we tentatively propose that compound **H_2_4** has the (*R*,S_p_^*^)‐ configuration and compound **H_2_5** the (*R*,R_p_^*^)‐one, but further studies, e.g. elucidation of the X‐ray structures of the compounds, are needed to substantiate these assignments.
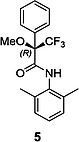



With the above information on the structures of the diastereomeric host compounds available, we may further interpret the measured binding constants (Table 1). Due to the upward orientation of the phenyl group in (*R*,S_p_*)‐**H_2_4** and the possibility of a hydrogen bond between the Mosher amide group and the carbonyl group of the diphenylglycoluril framework in this compound, fixating this upward orientation, the cavity of this host is partly blocked, making it less accessible for viologen guests than the cavity of (*R*,R_p_*)‐**H_2_5**. This orientation may also lead to the observed chiral discrimination between the two enantiomeric guests (*R*,*R*)‐**V2** and (*S*,*S*)‐**V2**.

In subsequent experiments, we further analyzed the structures of the complexes between hosts** H_2_4** and** H_2_5** and guests **V1** and **V2** by NMR. Complexation of** V1** to** H_2_1** has been described in our previous paper.^18^ This guest has two pyridinium rings that are slightly rotated with respect to each other. According to our previous NMR studies, these pyridinium rings of **V1** are sandwiched between the side‐walls of **H_2_1** and this orientation is stabilized by π‐π stacking interactions of the guest with the *o*‐xylylene rings and electrostatic interactions with the crown ether‐like rings. Complexation of **V1** to (*R*,S_p_*)‐**H_2_4** results in upfield shifts of most of the signals of the protons lining the inside of the cage, e.g. the side‐wall protons *30*, the oxyethylene protons *28*, and the pyrrole NH protons.^18^ Downfield shifts were observed for the signal of the amide protons *49* and the methoxy protons *53* of the Mosher substituent. From the various CISVs. (Table S1 of SI) it can be concluded that **V1** is located unsymmetrically between the walls, i.e. more pushed towards the wall opposite to that where the Mosher substituent is located (compare the CISVs. of the signals of the side‐wall protons *30*: +0.02, –0.18, and –0.29 ppm).

**Figure 4:**
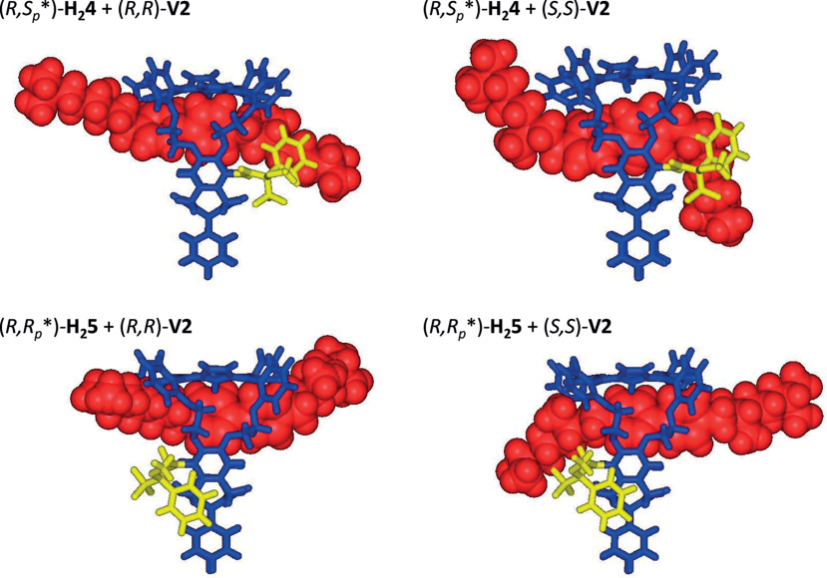
Calculated structures (Spartan '14) of the diastereomeric host–guest complexes based on the NMR experiments. The viologens guests are indicated space‐filling in red, the Mosher's substituent in yellow.

Binding of guests (*S*,*S*)‐ and (*R*,*R*)‐**V2** to host (*R*,S_p_*)‐**H_2_4** yields similar, but different spectra. The porphyrin pyrrole NH protons signal displayed similar shifts and CIS values as observed for the complex with **V1** indicating that the guests are also positioned perpendicular to the porphyrin planes. The CISVs. of the signals of the oxyethylene protons *27* and *28* were significantly different and from the shift values (see Table S2 of SI) it can be concluded that the guests are oriented asymmetrically in the complexes with (*R*,S_p_*)‐**H_2_4**, i.e. more to the back side of the host (under phenyl rings *III* and *IV*), where no substituent is present. This means that the centers of the guests are not aligned with the center of the cavity of (*R*,S_p_*)‐**H_2_4**, which can also be concluded from the CISVs. of the signals of the guest protons, which are different for the two sides of the guests (see Table S2 of SI). The amide proton signal of the Mosher substituent in (*R*,S_p_*)‐**H_2_4** was shifted downfield in both complexes with **V2**, but this shift was larger for the complex with the (*S*,*S*)‐guest (+0.31 ppm, see Table S2 in SI) than for the complex with the (*R*,*R*)‐guest (0.07 ppm). This indicates that the positive charge of the viologen moiety is closer to the Mosher amide group in the former case than in the latter, indicating that the (*S*,*S*)‐guest binds lower (more to the bottom) in the cavity of (*R*,S_p_*)‐**H_2_4** than the (*R*,*R*)‐guest (Figure 4). The methyl protons *4* of the latter guest have nOe interactions with the β‐pyrrole protons *3* and *4* of the porphyrin ring, i.e. the protons that are located at the side of the Mosher substituent. This indicates that this guest is positioned higher (more to the top) in the cage than the (*S*,*S*)‐guest, in line with what was concluded above. The lower location of the (*S*,*S*)‐guest was further checked with a 1D‐ROESY experiment, in which the protons of the methyl groups* 4* of the (*R*,*R*)‐ and (*S*,*S*)‐guests inside the cavity, were excited and the amount of magnetization transfer to the methoxy group of the Mosher substituent of (*R*,S_p_*)‐**H_2_4** was measured and compared (see Figure S72 of SI). From this experiment it appeared that more magnetization was transferred from the (*S*,*S*)‐guest to the Mosher methoxy substituent (1.06 %) than from the (*R*,*R*)‐guest (0.36 %), suggesting that the former is situated closer to the substituent and hence located lower in the cavity than the latter. It should be noted, however, that this is a qualitative experiment because the amount of NOE transfer might be dependent on the exchange rate, which could be different for the two guests.

The same types of NMR experiments were carried out with guests **V1** and **V2** and the diastereomeric host (*R*,R_p_*)‐**H_2_5**. In this host **V1** is also located unsymmetrically, i.e. more in the direction of the side wall where the Mosher substituent is not located (see CISVs. of the side wall protons *30* in Table S1 of SI). For the complexes with **V2** also upfield shifts of the signals of the pyrrole NH protons were observed, indicating a perpendicular orientation of the guests with respect to the porphyrin plane. The oxyethylene protons *27* and *28* displayed a similar shift pattern as observed for host (*R*,S_p_*)‐**H_2_4**, again suggesting an asymmetric binding of the guests, i.e. more to the backside of the host and away from the Mosher substituent (see Table S3 of SI). The protons at the lower part of the cage all became deshielded upon binding of the guest molecules. The largest (downfield) CIS values were found for the Mosher amide proton signal, i.e. +1.24 for the complex with the (*R*,*R*)‐guest and +1.11 ppm for the complex with the (*S*,*S*)‐guest. The methoxy protons of the Mosher substituent became only slightly deshielded after binding of the guests, but they did not show any nOe interactions with these molecules, in contrast to what was observed for the binding of the guests in the diastereomeric (*R*,S_p_*)‐**H_2_4** host. The CIS values of the protons of the guest molecules indicate that they are bound in an asymmetric fashion in the cavity, as could also be concluded from the CISVs. of the host oxyethylene protons* 27* and *28* (see above). The pyrrole NH protons and the β‐pyrrole protons *3/4* and *13/14* of the porphyrin ring displayed nOe interactions with the protons *10* and *11* of the viologen moieties, which indicates that both enantiomers of the** V2** guest reside in the upper part of host (*R*,R_p_*)‐**H_2_5**.

In conclusion, the NMR studies on the 4 diastereomeric host–guest complexes reveal that guests** V2** are bound higher, i.e. more in the upper part of the host, in (*R*,R_p_*)‐**H_2_5** than in (*R*,S_p_*)‐**H_2_4**, hence they are farther away from the chiral Mosher substituent (Figure 4). This is probably the result of the fact that the phenyl group of the Mosher substituent in (*R*,R_p_*)‐**H_2_5** points downwards, making room for binding at a higher location in the cage. This binding in the upper part of the host may explain why (*R*,R*)‐**H_2_5** does not discriminate well between the (*R*,*R*)‐guest and the (*S*,*S*)‐guest. The NMR studies revealed that the **V2** guests bind lower in host (*R*,S_p_*)‐**H_2_4** than in (*R*,R_p_*)‐**H_2_5**, hence, they will feel the influence of the Mosher substituent more strongly, leading to the observed discrimination between the (*R*,*R*)‐ and (*S*,*S*)‐forms. The studies further show that the (*S*,*S*)‐guest binds lower in the cavity of (*R*,S_p_*)‐**H_2_4** and is closer to the Mosher substituent than the (*R*,*R*)‐one, leading to stronger π‐π‐stacking and van der Waals interactions and hence to a higher binding constant (Table 1).

## Conclusion

We have synthesized the new chiral porphyrin cage compounds (*R*,S_p_*)‐**H_2_4** and (*R*,R_p_*)‐**H_2_5**, which are derived from the achiral diphenylglycoluril derivative **H_2_1**. The reaction sequence involved a regioselective nitration, followed by a reduction of the formed nitro‐compound to the amine, and functionalization of the latter with the chiral derivatizing agent Mosher's acid chloride. ^1^H‐NMR studies revealed that the Mosher group attached to the side‐wall of the toroidal‐shaped host compounds has different orientations in (*R*,S_p_*)‐**H_2_4** and (*R*,R_p_*)‐**H_2_5**, leading to different binding affinities for chiral viologen guest molecules. Compound (*R*,S_p_*)‐**H_2_4** is capable of discriminating between enantiomeric guests (by a factor of 2.8), whereas compound (*R*,R_p_*)‐**H_2_5** does not have this capability. Current work is directed towards threading experiments with chiral polymers with the objective to realize unidirectional movement of the chiral porphyrin cage compounds on the polymer chains. In subsequent studies these cage compounds will be provided with manganese centers to realize writing on chiral polymers chains in the form of (*R*,*R*)‐ and (*S*,*S*)‐epoxides (digits 1 and 0).

## Experimental Section

General Information: All chemicals were commercially obtained and used without further purification unless stated otherwise. ^1^H, ^13^C and ^19^F NMR spectra were collected on a Bruker 500 MHz Avance III spectrometer equipped with a Prodigy BB cryoprobe, a Bruker 400 MHz Avance III HD nanobay spectrometer equipped with a BBFO probe, or a Agilent 400 MHz INOVA spectrometer equipped with a dual‐channel inverse probe. All spectra were obtained at room temperature. Manual phase correction and automatic baseline correction were applied to all spectra. NMR data is presented as follows: chemical shift (in ppm relative to internal trimethylsilane), multiplicity, coupling constant in Hz, integration, assignment. Abbreviations used are: s=singlet, d=doublet, dd=doublet of doublets, t=triplets, dt=doublet of triplets, td=triplet of doublets and m=multiplet. All NMR signals were assigned on the basis of ^1^H NMR, ^13^C NMR, ^1^H‐^1^H COSY, ^1^H‐^13^C HSQC, ^1^H‐^15^N HSQC, ^1^H‐^13^C HMBC, ^1^H‐1^5^N HMBC, ^1^H‐^1^H ROESY and ^1^H TOCSY experiments. Mass spectra were recorded on a Thermo Finnigan LCQ Advantage Max mass spectrometer (MS) or on a Bruker Microflex LRF Maldi‐tof system in the reflective mode employing dithranol as a matrix.

**Synthesis. H_2_1** **and H_2_2:** These compounds were synthesized and characterized as reported previously.^6^, ^18^

(*R*,S_p_^*^)‐H_2_4 and (*R*,R_p_*)‐H_2_5: Compound **H_2_2** (33.7 mg, 24 µmol) and SnCl_2_ (184 mg, 969 µmol) were dissolved in HCl in dioxane (4 mL, 4 M) in a Schlenk‐bomb under an inert atmosphere. Concentrated HCl (10 µL) was added and the Schlenk‐bomb was closed. The suspension was stirred vigorously at 45 °C for 40 hours, after which it was quenched with aqueous NaOH (30 mL, 3 M). The aqueous layer was extracted with CHCl_3_ (2 × 20 mL). The combined organic layers were washed with aqueous NaOH (20 mL, 3 M), saturated aqueous NaHCO_3_ (20 mL), brine (20 mL), and subsequently evaporated to dryness. The residue was re‐dissolved in freshly distilled CHCl_3_ (1 mL), after which triethylamine (0.07 mL, 0.5 mmol) and (*R*)‐(‐)‐*α*‐methoxy‐*α*‐(trifluoromethyl)phenylacetyl chloride (12 µL, 64 µmol) were added. The mixture was stirred at 20 °C for 1 h and subsequently quenched with saturated aqueous NaHCO_3_ (20 mL). The aqueous layer was extracted with CHCl_3_ (10 mL). The organic layer was washed with saturated aqueous NaHCO_3_ (25 mL), brine (25 mL), and the solvents evaporated to dryness. The residue was purified by silica gel flash column chromatography (60H Silica, CHCl_3_/MeOH, 99.73:0.27, v/v) and subsequently by precipitation from CH_2_Cl_2_ and *n*‐heptane yielding (*R*,S_p_*)‐**H_2_4** (12.1 mg, 7.7 µmol, 32 %) and (*R*,R_p_*)‐**H_2_5** (6.2 mg, 3.9 µmol, 16 %) as purple solids.

(*R*,S_p_*)‐H_2_4: ^1^H NMR (500 MHz, [D]chloroform). The respective protons are indicated in bold. For numbering see Scheme 3. δ 9.17 (s, 1H, **49**), 8.95 (d, *J* = 4.7 Hz, 1H, **4**), 8.80 (d, *J* = 4.8 Hz, 1H, **3**), 8.79–8.75 (m, 2H, **8, 9, 13, 14, 18 or 19**), 8.70–8.66 (m, 3H, **8, 9, 13, 14, 18 or 19**), 8.63 (d, *J* = 4.8 Hz, 1H, **8, 9, 13, 14, 18 or 19**), 8.28 (dd, *J* = 7.3, 1.8 Hz, 1H, **22(II)**), 8.18 (dd, *J* = 7.3, 1.7 Hz, 1H, **22(I,III or IV)**), 8.14 (dd, *J* = 7.4, 1.7 Hz, 1H, **22(I,III or IV)**), 8.10 (dd, *J* = 7.4, 1.7 Hz, 1H, **22(I,III or IV)**), 7.80–7.71 (m, 3H, **24**(**I,III, IV)**), 7.66–7.59 (m, 1H, **24(II)**), 7.48 (d, *J* = 7.6 Hz, 2H, **55**), 7.45–7.33 (m, 7H, **23, 25(I,III,IV)**), 7.27–7.18 (m, 3H,** 56, 57**), 7.05–6.86 (m, 9H, **39–42, 44–48**), 6.74 (dd, *J* = 8.1, 1.8 Hz, 1H, **38**), 6.64 (d, *J* = 8.2 Hz, 1H, **25(II)**), 6.28 (s, 1H, **30(IV)**), 6.13 (s, 1H, **30(III)**), 6.11 (s, 1H, **30(I)**), 4.41 (d, *J* = 16.3 Hz, 1H, **32a(II)**), 4.27 (d, *J* = 15.7 Hz, 1H **32a(III)**), 4.22 (d, *J* = 15.6 Hz, 1H, **32a(I)**), 4.25–4.10 (m, 3H, **27(I), 27a(IV)**), 4.17 (d, *J* = 15.9 Hz, 1H **32a(IV)**), 4.00–3.87 (m, 2H, **27b(IV), 28a(II)**), 3.78 (d, *J* = 15.6 Hz, 1H, **32b(III)**), 3.73 (d, *J* = 15.8 Hz, 1H, **32b(I)**) 3.72 (d, *J* = 15.9 Hz, 1H, **32b(IV)**), 3.81–3.54 (m, 4H, **27a(II,III) or 28a(I,III)**), 3.57 (d, *J* = 16.3 Hz, 1H,** 32b(II)**), 3.45–3.37 (m, 3H, **27b(II), 28b(I,II)**), 3.25 (s, 4H, **27b(III), 53**), 3.20 (t, *J* = 5.7 Hz, 2H, **28(IV)**), 3.09–3.02 (m, 1H, **28b(III)**), –2.69 (s, 2H, pyr‐NH). **^13^C NMR** (126 MHz, [D]chloroform) δ 165.00 (**50**), 159.71 (**26**), 159.05 (**26**), 158.98 (**26**), 158.93 (**26**), 157.71 (**33**), 156.91 (**34**), 156.10, 150.49(**29(III)**), 147.57 (**29(I)**), 146.40 (**29(IV)**), 141.97 (**29(II)**), 135.94 (**22**), 135.90 (**22**), 135.80 (**22**), 135.37 (**22**), 133.85 (**37 or 43**), 133.22 (**37 or 43**), 132.73 (**21(I,III or IV)**), 132.51 (**31(III)**), 132.14 (**21(I,III or IV)**), 131.99 (**21(I,III or IV)**), 131.32 (**54**), 130.81 (**31(I) or 21(II)**), 130.74 (**31(I) or 21(II)**), 129.71, 129.67, 129.57, 129.54, 129.43, 129.35 (**31(IV)**), 128.70, 128.66, 128.62, 128.51, 128.40, 128.38, 128.33, 127.83, 127.59, 127.36 (**30(II)**), 124.84 (**31(II)**), 120.77 (**23(I,III or IV)**), 120.13 (**23(I,III or IV)**), 119.93 (**23(I,III or IV)**), 119.43 (**23(II)**), 117.99 (**30(IV)**), 115.61 (**6**), 115.46 (**1, 11, 16 or 25)**, 115.33 (**1, 11, 16 or 25)**, 115.27 (**1, 11, 16 or 25)**, 114.96 (**1, 11, 16 or 25)**, 114.79 (30(I)), 112.91 (**25**), 112.17 (**1, 11, 16 or 25 or 30 (III))**, 112.14 (**1, 11, 16 or 25 or 30 (III))**, 112.05 (**1, 11, 16 or 25 or 30 (III))**, 85.29 (**35**), 84.64 (**36**), 72.26, 71.14 (**27 or 28**), 69.26 (**27 or 28**), 68.58 (**27 or 28**), 68.43 (**27 or 28**), 67.59 (**27 or 28**), 67.41 (**27 or 28**), 67.12 (**27 or 28**), 61.90, 55.02 (**53**), 44.59 (**32(I,III or IV)**), 44.22 (**32(I,III or IV)**), 39.23 (**32**(II)). **^19^F NMR** (471 MHz, [D]chloroform) δ –69.22 (**52**). **MALDI‐TOF** (*m/z*): [M + H]^+^ calculated for C_94_H_72_F_3_N_9_O_12_+H^+^, 1577.54; found 1577.355.** HRMS** (*m/z*): [M + Na]^+^_ calcd._ for C_94_H_72_F_3_N_9_Na_1_O_12_, 1598.51502; found 1598.51431. **UV/Vis:** *λ*_max_ = 420 nm. **Fluorescence: *λ*** = 653 and 719 nm (c = 3 µM in CHCl_3_/MeCN, 1:1, v/v, *T* = 295 K, λ_ex_ = 420 nm). M.p. 292 °C. No optical rotation could be measured because solutions of this compound were too colored. For CD‐spectrum, see Figure 2.

(*R*,R_p_*)‐H_2_5: ^1^H NMR (500 MHz, [D]chloroform) δ 8.78 (d, *J* = 4.8 Hz, 1H,** 3, 4**, **8, 9, 13, 14, 18 or 19**), 8.76–8.70 (m, 4H,** 3, 4**, **8, 9, 13, 14, 18 or 19**), 8.69–8.61 (m, 3H,** 3, 4**, **8, 9, 13, 14, 18 or 19**), 8.28 (dd, *J* = 7.4, 1.7 Hz, 1H, **22**), 8.16 (dd, *J* = 7.4, 1.8 Hz, 1H, **22**), 8.13 (s, 1H, **49**), 8.11 (dd, *J* = 7.5, 1.6 Hz, 1H, **22**), 8.07 (dd, *J* = 7.4, 1.7 Hz, 1H, **22**), 7.80–7.73 (m, 4H, **24**), 7.60–7.56 (m, 2H, **55**), 7.48–7.25 (m, 11H, **23, 25, 56, 57**), 6.99–6.86 (m, 6H, **39, 40, 41, 45, 46, 47**), 6.83–6.79 (m, 1H, **38**), 6.76 (d, *J* = 8.1 Hz, 1H, **44 or 48**), 6.72 (dd, *J* = 7.5, 2.0 Hz, 1H, **44 or 48**), 6.57–6.52 (m, 1H, **42**), 6.22 (s, 1H, **30(III)**), 6.16 (s, 1H, **30(IV)**), 6.13 (s, 1H, **30(II)**), 4.49 (td, *J* = 9.9, 2.5 Hz, 1H, **27(I)**), 4.29–4.09 (m, 3H, **27(II), 27a(III)**), 4.21 (d, *J* = 15.6 Hz, 1H, **32a(IV)**), 4.15 (d, *J* = 15.9 Hz, 1H, **32a(II or III)**), 4.15 (d, *J* = 15.7 Hz, 1H, **32a(II or III)**), 4.04 (d, *J* = 9.9 Hz, 1H, **27b(I)**), 4.02–3.86 (m, 3H, **27a(IV), 27b(III), 28a(I)**), 3.78–3.50 (m, 6H, **27b(IV), 28a(III,IV), 28b(I), 32a(I), 32b(II,III,IV**), 3.46–3.38 (m, 1H, **28b(II)**), 3.30–3.22 (m, 4H, **32b(I), 53**), 3.22–3.14 (m, 2H), 3.14–3.08(m, 1H, **28b(IV)**), –2.69 (s, 2H). **^13^C NMR** (126 MHz, [D]chloroform) δ 165.46 (**50**), 159.48 (**26**), 159.04 (**26**), 158.79 (**26**), 157.21 (**33**), 156.86 (**34**), 149.76 (**29(IV)**), 147.45 (**29(II)**), 146.44 (**29(III)**, 142.71, 142.21 (**29(I)**), 135.96 (**22**), 135.91 (**22**), 135.40 (**22**), 133.83, 133.53, 133.23, 132.48, 132.09, 132.00, 131.87 (**31(IV)**), 131, 13 130.60 (**31(II)**), 129.87, 129.65, 129.36 (**31(III)**), 129.11, 128.62, 128.55, 128.51, 128.50, 128.46, 128.36, 128.28, 128.22, 128.17, 127.98, 127.76, 127.31 (**55**), 126.62 (**30(I)**), 125.43 (**31(I)**), 120.43 (**23)**, 120.06 (**23)**, 119.90 (**23)**, 119.71 (**23)**, 115.50 (**1, 6, 11 or 16**), 115.21 (**1, 6, 11 or 16**), 115.13 (**1, 6, 11 or 16**), 115.01 (**1, 6, 11 or 16**), 114.94, 114.90 (**30(II)**), 114.12 (**25**), 112.77 (**25 or 30(IV)**), 112.61 (**25 or 30(IV)**), 112.09 (**25**), 111.71 (**25**), 85.09 (**36**), 84.41 (**35**), 71.26 (**28(I)**), 68.60 (**27(IV)**), 68.52 (**27(I)**), 68.43 (**28(III)**), 67.39 (**27(III)**), 67.33 (**27(I)**), 67.21 (**28(II)**), 67.11 (**28(III)**), 55.51 (**53**), 44.62 (**32(III)**), 44.40 (**32(II)**), 44.22 (**32(IV)**), 38.42 (**32(I)**). **^19^F NMR** (471 MHz, [D]chloroform) δ –68.42 (**52**). **MALDI‐TOF:** *m/z*: 1576.562 [M + H]^+^; calculated for C_94_H_72_F_3_N_9_O_12_ +H^+^: *m/z*: 1576.53. **HRMS:** (*m/z*): [M + H]^+^_ calcd._ for C_94_H_73_F_3_N_9_O_12_, 1576.53308; found 1576.53376. UV/Vis: *λ*_max_ = 420 nm. Fluorescence:***λ*** = 653 and 719 nm (c = 3 µM in CHCl_3_/MeCN, 1:1, v/v, *T* = 295 K, λ_ex_ = 420 nm). M.p. 150 °C. No optical rotation could be measured because solutions of this compound were too colored. For CD‐spectrum, see Figure 2.

**(*R*)‐*N*‐(2,6‐dimethylphenyl)‐3,3,3‐trifluoro‐2‐methoxy‐2‐phenylpropanamide (Mosher model):** A Schlenk flask was evacuated three times and 2,6‐dimethylaniline (51 µL, 0.41 mmol) and (*R*)‐3,3,3‐trifluoro‐2‐methoxy‐2‐phenylpropanoyl chloride (0.19 mL, 1.0 mmol) were dissolved in dry chloroform (10 mL) and this solution was added to the flask. Triethylamine (1 mL) was added dropwise and the resulting mixture was stirred at room temperature for 16 hours. The reaction was quenched by adding saturated aqueous NaHCO_3_ and the mixture was extracted with chloroform. The organic layer was washed with NaHCO_3_ (2 ×) and brine (2 ×). The organic layers were combined, dried with NaSO_4_, and concentrated in vacuo to give the desired product as a white powder. Yield 0.11 g (0.31 mmol, 76 %). M.p. 124 °C.
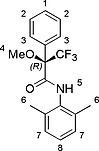



^1^H NMR (500 MHz, [D]Chloroform) δ 8.17 (s, 1H, **5**), 7.69–7.62 (m, 2H, **3**), 7.47–7.39 (m, 3H, **1**,** 2**), 7.12–7.07 (m, 1H), 7.09–7.03 (m, 3H, **7, 8**), 3.53 (q, *J* = 1.6 Hz, 3H, **4**), 2.18 (s, 6H, **6**). **HRMS** *(m/z)*: [M + H]^+^_ calcd._ for C_18_H_18_F_3_NNaO_2_, 360.11873; found 360.11848.

**Synthesis of guest compounds**

**Scheme 4:**
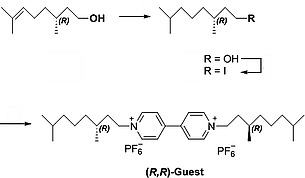
Synthesis of (*R*,*R*)‐**V2**.

Compound **V1** was a commercial product. Compounds (*R*,*R*)‐ and (*S*,*S*)‐**V2** were synthesized as shown in Scheme 4 for the (*R*,*R*)‐ derivative.
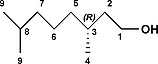



**(*R*)‐3,7‐dimethyloctan‐1‐ol:** Palladium on carbon (35 mg, 33 µmol, 10 wt.‐%) was submerged in CH_2_Cl_2_ before MeOH (10 mL) was added. *(R)*‐3,7‐dimethyl‐6‐octen‐1‐ol (0.12 mL, 0.66 mmol) was added under inert atmosphere and the mixture was bubbled through with H_2_ at 20 °C for 4 hours, after which the suspension was filtered through Celite. The filtrate was concentrated in vacuo, yielding *(R)*‐3,7‐dimethyloctan‐1‐ol (0.11 g, 0.68 mmol, quantitative yield) as a colourless oil. **^1^H NMR** (500 MHz, [D]chloroform) δ 3.77–3.62 (m, 2H, **1**), 1.65–1.47 (m, 3H, **2a, 3, 8**), 1.42–1.20 (m, 4H, **2b, 5a, 6a, 7a**), 1.20–1.07 (m, 4H, **5b, 6b, 7b**), 0.91–0.84 (m, 9H, **4, 9**). **^13^C NMR** (126 MHz, [D]chloroform) δ 61.29 (**1**), 40.02 (**2**), 39.26 (**7**), 37.37 (**5**), 29.51 (**3**), 27.97 (**8**), 24.69 (**6**), 22.70 (**9**), 22.60 (**9**), 19.65 (**4**).^21^

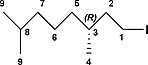



**(*R*)‐1‐iodo‐3,7‐dimethyloctane:** PPh_3_ (1.03 g, 3.9 mmol) and imidazole (0.39 g, 5.7 mmol) were added to a degassed solution of *(R)*‐3,7‐dimethyloctan‐1‐ol (0.31 g, 2.0 mmol) in MeCN (3 mL) and Et_2_O (3 mL). Iodine (0.63 g, 2.5 mmol) was added under inert atmosphere at 0 °C. The mixture was stirred for 30 min at 0 °C and for 2 hours at 20 °C, after which it was diluted with *n*‐heptane (20 mL). The organic layer was washed with a saturated aqueous solution of NaS_2_O_3_ (20 mL), a saturated aqueous solution of CuSO_4_ (20 mL), brine (20 mL), dried with MgSO_4_, and concentrated in vacuo. The residue was purified by silica gel flash column chromatography (eluent *n*‐heptane/EtOAc, 9:1, v/v) yielding *(R)*‐1‐iodo‐3,7‐dimethyloctane (0.39 g, 1.5 mmol, 74 %) as a colourless oil. **^1^H NMR** (500 MHz, [D]chloroform) δ 3.25 (ddd, *J* = 9.6, 8.6, 5.7 Hz, 1H, **1a**), 3.17 (ddd, *J* = 9.5, 8.3, 7.2 Hz, 1H, **1b**), 1.87 (dddd, *J* = 13.9, 8.5, 7.2, 5.4 Hz, 1H, **2a**), 1.64 (dtd, *J* = 13.9, 8.0, 5.7 Hz, 1H, **2b**), 1.60–1.45 (m, 2H, **3, 8**), 1.38–1.06 (m, 6H, **5, 6, 7**), 0.91–0.82 (m, 9H, **4,9**). **^13^C NMR** (126 MHz, [D]chloroform) δ 41.13 (**2**), 39.32 (**7**), 36.62 (**5**), 34.03 (**3**), 28.09 (**8**), 24.67 (**6**), 22.84 (**9**), 22.74 (**9**), 18.89 (**4**), 5.50 (**1**).^21^

**1,1′‐Bis(*R*)‐3,7‐dimethyloctyl‐4,4′‐bipyridi‐1,1′‐diium dihexafluorophosphate (*R*,*R*)‐V2**: 4,4′‐bipyridine (0.14 g, 0.90 mmol) and *(R)*‐1‐iodo‐3,7‐dimethyloctane (0.96 g, 3.6 mmol) were suspended in DMF (4 mL). The mixture was stirred at 100 °C for 40 hours. After cooling the reaction mixture Et_2_O (10 mL) was added and the formed precipitate was filtered off. The orange solid was washed with Et_2_O (10 mL) and dried on the air. The orange solid was dissolved in the smallest possible amount of boiling MeCN, after which a saturated aqueous solution of NH_4_PF_6_ (5 mL) was added dropwise. The layers were separated and the yellow MeCN layer was concentrated in vacuo. The yellow precipitate was filtered off and recrystallized from MeOH yielding 1,1′‐bis((*R*)‐3,7‐dimethyloctyl‐4,4′‐bipyridi‐1,1′‐diium di hexafluorophosphate (0.18 g, 0.25 mmol, 28 %) as a nearly white solid. M.p. 279 °C (decomposition). [*α*]^20^_D_ –0.018° (*c* 2.451, CH_3_CN). **^1^H NMR** (500 MHz, [D_3_]acetonitrile) (for proton numbering see Scheme 3). δ 8.93–8.88 (m, 4H, **10**), 8.38–8.34 (m, 4H, **11**), 4.71–4.58 (m, 4H, **1**), 2.07–1.98 (m, 2H, **2a**), 1.84 (dddd, *J* = 13.7, 11.6, 8.8, 4.8 Hz, 2H, **2b**), 1.63–1.48 (m, 4H, **3, 8**), 1.42–1.13 (m, 12H, **5, 6, 7**), 1.01 (d, *J* = 6.7 Hz, 6H, **4**), 0.88 (d, *J* = 6.6 Hz, 12H, **9**). **^13^C NMR** (126 MHz, [D_3_]acetonitrile) δ 150.81 (**12**), 146.59 (**10**), 128.17 (**11**), 61.64 (**1**), 39.83 (**7**), 39.13 (**2**), 37.48 (**5**), 31.28 (**3**), 28.71 (**8**), 25.25 (**6**), 22.93 (**9**), 22.83 (**9**), 19.48 (**4**).** HRMS** *(m/z)*: [M – PF_6_]^+^ calcd. for C_30_H_50_F_6_N_2_P_1_, 583.36029; found 583.36093.

**1,1′‐Bis(*S*)‐3,7‐dimethyloctyl‐4,4′‐bipyridi‐1,1′‐diium dihexafluorophosphate (*S*,*S*)‐V2:** this compound was prepared as described for the (*R*,*R*)‐ derivative starting from (*S*)‐3,7‐dimethyloctan‐1‐ol. M.p. 283 °C (decomposition). [*α*]^20^_D_ +0.019 (*c* 2.453, CH_3_CN).**^ 1^H NMR** (500 MHz, [D_3_]acetonitrile) (for proton numbering see Scheme 3). δ 8.93 (d, *J* = 6.9 Hz, 4H, **10**), 8.39 (d, *J* = 6.4 Hz, 4H, **11**), 4.71–4.58 (m, 4H, **1**), 2.09–1.99 (m, 2H, **2a**), 1.85 (dddd, *J* = 13.6, 10.1, 8.0, 5.9 Hz, 2H, **2b**), 1.62–1.50 (m, 4H, **3, 8**), 1.42–1.14 (m, 12H, **5, 6, 7**), 1.01 (d, *J* = 6.6 Hz, 6H, **4**), 0.88 (d, *J* = 6.6 Hz, 12H, **9**). **^13^C NMR** (126 MHz, [D_3_]acetonitrile) δ 150.82 (**12**), 146.59 (**10**), 128.17 (**11**), 61.62 (**1**), 39.84 (**7**), 39.13 (**2**), 37.49 (**5**), 31.28 (**3**), 28.71 (**8**), 25.24 (**6**), 22.94 (**9**), 22.83 (**9**), 19.48 (**4**). **HRMS** *(m/z)*: [M – PF_6_]^+^ calcd. for C_30_H_50_F_6_N_2_P_1_, 583.36029; found 583.36029.

**Host–guest complexes**

(*R*,S_p_*)‐H_2_4 with (*R*,*R*)‐V2: ^1^H NMR (500 MHz, [D_3_]acetonitrile: [D]chloroform, 1:1). Guest peaks are marked red: δ 9.29 (d, *J* = 4.8 Hz, 1H, **4**), 9.18 (d, *J* = 4.7 Hz, 1H, **8 or 9**), 9.15 (d, *J* = 4.8 Hz, 1H, **3**), 9.14 (d, *J* = 4.7 Hz, 1H, **8 or 9**), 9.05 (s, 1H, **49**), 8.92 (s, 0H, **V10 unbound**), 8.86 (d, *J* = 4.8 Hz, 2H, **8, 9, 18 or 19**), 8.83–8.75 (m, 2H, **8, 9, 18 or 19**), 8.40 (s, 1H, **V11 unbound**), 8.32 (dd, *J* = 7.4, 1.7 Hz, 1H, **22(II)**), 8.22 (dd, *J* = 7.4, 1.7 Hz, 1H, **22(I)**), 8.15–8.08 (m, 2H, **22(III,IV)**), 7.91–7.81 (m, 4H, **24**), 7.63–7.45 (m, 7H, **23, 25(I,III,IV)**), 7.47–7.25 (m, 8H, **55, 56, 57**, **V10 out**), 7.19–6.96 (m, 9H, **39–42, 44–48**), 6.95 (d, *J* = 8.2 Hz, 1H, **38**), 6.91 (d, *J* = 8.3 Hz, 1H, **25(II)**), 6.08 (s, 1H, **30(IV)**), 6.07 (s, 1H, **30(III)**), 5.88 (s, 1H, **30(I)**), 5.21 (s, 2H, **V10 in**), 4.93 (s, 2H, **V11 out**), 4.63 (d, *J* = 23.9 Hz, 1H,** V1 unbound**), 4.38 (d, *J* = 16.0 Hz, 1H, **32a(III)**), 4.33–4.20 (m, 5H, **27a(III,IV), 32a(I,II,IV)**), 4.15 (t, *J* = 9.1 Hz, 2H, **27a(I), V1a out**), 4.09–3.96 (m, 4H, **27b(I,III,IV), V1b out**), 3.90 (d, *J* = 16.1 Hz, 1H, **32b(III)**), 3.87 (d, *J* = 16.4 Hz, 1H, **32b(I)**), 3.83 (t, *J* = 16.2 Hz, 1H, **32b(IV)**), 3.80–3.74 (m, 1H, **27a(I)**), 3.68 (d, *J* = 16.5 Hz, 1H, **32b(II)**), 3.65–3.46 (m, 5H, **27b(I)**,** V11 in**), 3.40–3.23 (m, 9H, **28a, 53**,** V1a in**), 3.09–3.02 (m, 1H, **28b(II)**), 2.96–2.86 (m, 1H, **V1b in**), 2.84–2.76 (m, 1H, **28b(I)**), 2.65 (t, *J* = 10.3 Hz, 1H, **28b(III)**), 2.46 (t, *J* = 10.0 Hz, 1H, **28b(IV)**), 1.72–0.82 (m, 50H), 0.77 (d, *J* = 6.7 Hz, 3H, **V4 in**), 0.74–0.68 (m, 1H, **V2a in**), 0.39 (s, 1H, **V2b in**), –2.81 (s, 2H, pyr‐NH). ^13^C NMR (126 MHz, [D_3_]acetonitrile: [D]chloroform, 1:1) δ 165.72, 160.11, 159.97, 159.66, 159.60, 158.69, 158.36, 151.03, 148.16, 147.31, 145.91, 145.76, 145.05, 142.95, 142.93, 142.91, 142.52, 136.76, 136.63, 136.43, 134.76, 133.93, 133.47, 132.94, 132.46, 132.20, 131.87, 131.75, 131.70, 131.61, 131.44, 131.40, 131.30, 130.89, 130.81, 130.41, 130.29, 130.26, 129.91, 129.86, 129.81, 129.61, 129.07, 129.02, 128.94, 128.37, 128.34, 125.28, 123.08, 121.99, 121.78, 121.66, 117.70, 117.61, 117.36, 117.19, 114.58, 114.22, 114.05, 113.85, 113.83, 97.19, 87.15, 86.42, 71.14, 70.06, 69.30, 68.84, 68.28, 61.52, 61.40, 56.24, 45.56, 45.18, 44.74, 40.28, 40.07, 39.87, 38.91, 37.81, 37.36, 32.85, 31.86, 30.62, 30.24, 29.01, 25.65, 25.42, 23.65, 23.61, 23.58, 23.49, 23.37, 23.30, 19.84, 19.49, 14.85. ^19^F NMR (471 MHz, [D_3_]acetonitrile: [D]chloroform, 1:1) δ –69.15 (**52**), –73.78 (d, *J* = 708.1 Hz, PF_6_).

(*R*,S_p_*)‐H_2_4 with (*S*,*S*)‐V2: ^1^H NMR (500 MHz, [D_3_]acetonitrile: [D]chloroform, 1:1). Guest protons are marked red: δ 9.31 (d, *J* = 4.7 Hz, 1H, **4**), 9.29 (s, 1H, **49**), 9.21 (d, *J* = 4.7 Hz, 1H, **8 or 9**), 9.15 (d, *J* = 4.7 Hz, 1H, **8 or 9**), 9.13 (d, *J* = 4.8 Hz, 1H, **3**), 8.94 (d, *J* = 6.3 Hz, 2H, **V10 unbound**), 8.87 (d, *J* = 4.8 Hz, 2H, **8, 18 or 19**), 8.79 (d, *J* = 4.8 Hz, 1H, **9**), 8.76 (d, *J* = 4.9 Hz, 1H, **18 or 19**), 8.42 (d, *J* = 6.2 Hz, 2H, **V11 unbound**), 8.30 (dd, *J* = 7.4, 1.7 Hz, 1H, **22(II)**), 8.21 (dd, *J* = 7.4, 1.7 Hz, 1H, **22(I)**), 8.15–8.09 (m, 2H, **22(III,IV)**), 7.94–7.79 (m, 6H, **24**, **V10 out**), 7.63–7.31 (m, 12H, **23, 25(I,III,IV), 55, 56, 57**), 7.18–6.98 (m, 9H, **39–42, 44–48**), 6.97–6.92 (m, 1H, **38**), 6.88 (d, *J* = 8.3 Hz, 1H, **25(II)**), 6.09 (s, 1H, **26(IV)**), 6.04 (s, 1H, **26(III)**), 5.93 (s, 1H, **26(I)**), 5.46 (s, 2H, **V11 out**), 4.73–4.48 (m, 3H, **V1 unbound**, **V10 in**), 4.39 (d, *J* = 16.0 Hz, 1H, **32a(III)**), 4.38–4.28 (m, 2H, **V1 out**), 4.28–4.19 (m, 6H, **27a(III,IV), 32a(I,II,IV)**), 4.15 (t, *J* = 9.3 Hz, 1H, **27a(I)**), 4.05–3.94 (m, 3H, **27b(I,III,IV)**), 3.90 (d, *J* = 15.9 Hz, 1H, **32b(III)**), 3.87 (d, *J* = 15.9 Hz, 1H, **32b(I)**), 3.83 (d, *J* = 15.9 Hz, 1H, **32b(IV)**), 3.79 (d, *J* = 10.2 Hz, 1H, **27a(II)**), 3.70 (d, *J* = 16.5 Hz, 1H, **32b(II)**), 3.68–3.57 (m, 2H, **27b(II)**), 3.40–3.30 (m, 5H, **28b**, **V11 in**), 3.28 (s, 3H, **53**), 3.27–3.16 (m, 1H, **V1a in**), 3.11 (d, *J* = 9.8 Hz, 1H, **28b(II)**), 2.90 (d, *J* = 10.9 Hz, 1H, **28b(I)**), 2.52 (t, *J* = 10.4 Hz, 1H, **28b(III)**), 2.49–2.36 (m, 1H, **V1b in**), 2.34–2.26 (m, 1H, **28b(IV)**), 1.90–0.77 (m, 44H), 0.69 (d, *J* = 6.5 Hz, 3H, **V4 in**), 0.52–0.37 (m, 1H, **V2a in**), 0.01 to –0.12 (m, 1H, **V2b in**), –2.81 (s, 2H, pyr‐NH). ^13^C NMR (126 MHz, [D_3_]acetonitrile: [D]chloroform, 1:1) δ 165.29, 159.74, 159.45, 159.32, 159.16, 159.03, 158.72, 150.57, 146.81, 146.24, 146.17, 146.16, 146.10, 145.59, 145.02, 141.90, 141.47, 141.22, 137.87, 136.22, 136.06, 135.97, 135.91, 134.20, 133.34, 132.83, 132.44, 131.76, 131.47, 131.38, 131.12, 131.08, 131.00, 130.92, 130.76, 130.71, 130.22, 129.86, 129.71, 129.68, 129.33, 129.27, 129.22, 129.03, 128.52, 128.42, 128.33, 127.89, 127.80, 125.28, 122.17, 122.15, 121.39, 121.08, 121.01, 119.58, 117.08, 116.54, 116.34, 113.21, 113.14, 113.07, 86.61, 85.88, 69.23, 68.46, 67.59, 67.44, 61.33, 60.97, 60.74, 55.52, 45.01, 44.58, 44.17, 39.71, 39.55, 39.49, 39.26, 39.09, 38.94, 37.49, 37.18, 31.28, 31.21, 31.00, 28.42, 28.39, 28.34, 25.35, 25.02, 24.90, 23.08, 23.02, 22.91, 22.83, 22.73, 19.39, 19.31, 18.52. ^19^F NMR (471 MHz, [D_3_]acetonitrile: [D]Chloroform, 1:1) δ –69.14 (**52**), –73.76 (d, *J* = 708.4 Hz, PF_6_).

(*R*,R_p_*)‐H_2_5 with (*R*,*R*)‐V2: ^1^H NMR (500 MHz, [D_3_]acetonitrile: [D]chloroform, 1:1). Guest protons are marked red: δ 9.31 (s, 1H, **49**), 9.21 (d, *J* = 4.7 Hz, 1H, **13 or 14**), 9.20 (d, *J* = 4.7 Hz, 1H, **3 or 4**), 9.13 (d, *J* = 4.7 Hz, 1H, **13 or 14**), 9.03 (d, *J* = 4.8 Hz, 1H, **3 or 4**), 8.93 (d, *J* = 4.7 Hz, 3H, **19, V10 unbound**), 8.83 (d, *J* = 4.7 Hz, 1H, **8 or 9**), 8.80 (d, *J* = 4.8 Hz, 1H, **18**), 8.76 (d, *J* = 4.8 Hz, 1H, **8 or 9**), 8.44–8.39 (m, 1H**, V11 unbound**), 8.35 (dd, *J* = 7.4, 1.7 Hz, 1H, **22**), 8.15 (dd, *J* = 7.2, 1.8 Hz, 2H, **22**), 8.10 (dd, *J* = 7.5, 1.8 Hz, 1H, **22**), 7.93–7.82 (m, 4H, **24**), 7.70–7.57 (m, 2H, **V10 out**), 7.54–7.35 (m, 28H, **23, 25, 55, 56, 57, CHCl_3_**), 7.25–6.97 (m, 9H, **38–42, 45–48**), 6.96–6.91 (m, 1H, **44**), 6.07 (s, 1H, **30(III)**), 6.04 (s, 1H, **30(IV)**), 5.96 (s, 1H, **30(II)**), 5.14 (s, 2H, **V11 out**), 4.93 (s, 2H, **V10 in**), 4.75–4.54 (m, 1H, **V1 unbound**), 4.38 (d, *J* = 16.0 Hz, 1H, **32a(IV)**), 4.32–4.11 (m, 9H, **27a, 32a(I,II,III)**, **V1 out**), 4.06–3.98 (m, 3H, **27b(II,III,IV)**), 3.97–3.88 (m, 2H, **27b(I), 32b(IV)**), 3.86 (d, *J* = 13.2 Hz, 1H, **32b(II)**), 3.83 (d, *J* = 16.0 Hz, 1H, **32b(III)**), 3.75 (d, *J* = 16.3 Hz, 1H, **32b(I)**), 3.49–3.40 (m, 1H, **V1a in**), 3.39–3.30 (m, 7H, **28a**, **V11 in**), 3.27 (s, 3H, **53**), 3.17 (d, *J* = 26.5 Hz, 1H, **28b(I)**), 2.91 (d, *J* = 8.7 Hz, 1H, **28b(II)**), 2.66 (s, 1H, **V1b in**), 2.52 (t, *J* = 10.5 Hz, 1H, **28b(IV)**), 2.35 (t, *J* = 9.8 Hz, 1H, **28b(III)**), 1.70–0.86 (m, 76H), 0.86 (d, *J* = 6.6 Hz, 3H, **V4 in**), 0.62 (d, *J* = 11.8 Hz, 1H, **V2a in**), 0.20–0.04 (m, 1H, **V2b in**), –2.83 (s, 2H, pyr‐NH). ^13^C NMR (126 MHz, [D_3_]acetonitrile: [D]chloroform, 1:1) δ 165.01, 159.61, 159.50, 159.39, 158.96, 155.70, 150.78, 147.49, 145.04, 145.02, 142.29, 136.50, 136.37, 136.19, 134.31, 133.71, 133.17, 131.69, 131.50, 131.45, 131.27, 131.21, 131.14, 131.12, 131.02, 130.95, 130.59, 130.10, 130.04, 129.75, 129.64, 129.60, 129.53, 129.42, 129.40, 129.14, 128.75, 128.72, 128.70, 125.29, 122.44, 121.70, 121.41, 121.39, 117.43, 113.64, 113.30, 87.02, 86.24, 68.70, 68.56, 68.12, 67.67, 64.15, 62.73, 62.67, 62.52, 61.27, 40.23, 40.08, 39.85, 39.58, 37.86, 37.56, 31.79, 31.63, 28.83, 28.80, 28.73, 28.53, 26.42, 25.77, 25.43, 23.53, 23.44, 23.32, 23.29, 23.18, 19.78, 18.68, 14.71, 14.65. ^19^F NMR (471 MHz, [D_3_]acetonitrile: [D]chloroform, 1:1) δ –68.80 (**52**), –73.79 (d, *J* = 708.6 Hz, PF_6_).

(*R*,R_p_*)‐H_2_5 with (*S*,*S*)‐V2: ^1^H NMR (500 MHz, [D_3_]acetonitrile: [D]chloroform, 1:1). Guest protons are marked red: δ 9.25–9.15 (m, 3H, **3, 4, 13 or 14, 49**), 9.12 (d, *J* = 4.7 Hz, 1H, **3, 4, 13 or 14)**, 9.04 (d, *J* = 4.7 Hz, 1H, **3, 4, 13 or 14)**, 8.95 (d, *J* = 6.3 Hz, 3H,** V10 unbound**), 8.93–8.89 (m, 1H, **19**), 8.84–8.74 (m, 3H, **8, 9, 18**), 8.44 (d, *J* = 6.2 Hz, 3H,** V11 unbound**), 8.35 (dd, *J* = 7.4, 1.7 Hz, 1H, **22**), 8.18–8.11 (m, 2H, **22**), 8.09 (dd, *J* = 7.3, 1.8 Hz, 1H, **22**), 7.93–7.82 (m, 4H, **24**), 7.62–7.36 (m, 34H, **23, 25, 55, 56, 57**CHCl_3_), 7.17 (d, *J* = 6.0 Hz, 2H,** V10 out**), 7.17–6.97 (m, 9H, **38–42, 45–48**), 6.93 (d, *J* = 7.9 Hz, 1H, **44**), 6.11 (s, 1H, **30(IV)**), 6.10 (s, 1H, **30(III)**), 5.97 (s, 1H, **30(II)**), 5.52 (s, 2H,** V10 in**), 4.75 (s, 2H,** V11 out**), 4.73–4.56 (m, 2H,** V1 unbound**), 4.39 (d, *J* = 16.0 Hz, 1H, **32a(IV)**), 4.34–4.16 (m, 7H, **27a, 32a(I,II,III)**), 4.09–3.98 (m, 8H, **27b(I,II,III)**,** V1a out**), 3.99–3.78 (m, 5H, **27b(IV), 32b(II,III,IV)**,** V1b out**), 3.78–3.50 (m, 7H, **32b(I)**,** V11 in, V1a in**), 3.43–3.34 (m, 4H, **28a**), 3.29 (s, 3H, **53**), 3.26–3.14 (m, 1H, **28b(I)**), 3.16–3.04 (m, 1H,** V1b in**), 2.86 (s, 1H, **28b(II)**), 2.69 (s, 1H, **28b(IV)**), 2.50 (t, *J* = 9.6 Hz, 1H, **28b(III)**), 1.92–1.09 (m, 41H), 1.04–0.95 (m, 19H), 0.90–0.87 (m, 9H), 0.84 (d, *J* = 6.6 Hz, 3H,** V4 in**), 0.49 (s, 1H,** V2b in**). ^13^C NMR (126 MHz, [D_3_]acetonitrile: [D]chloroform, 1:1) δ 157.92, 157.71, 157.62, 157.17, 146.27, 146.11, 145.64, 144.81, 143.54, 142.78, 134.84, 134.66, 134.44, 132.66, 131.96, 131.42, 130.78, 130.33, 130.06, 129.84, 129.80, 129.72, 129.50, 129.36, 129.30, 128.92, 128.82, 128.35, 128.33, 128.29, 128.11, 128.00, 127.89, 127.85, 127.75, 127.74, 127.63, 127.38, 126.95, 126.46, 122.95, 121.11, 121.10, 121.03, 119.93, 119.79, 119.74, 116.62, 112.15, 111.95, 111.78, 111.69, 85.26, 84.46, 69.34, 69.05, 67.85, 67.83, 67.14, 67.03, 66.44, 66.15, 60.01, 59.46, 54.54, 54.16, 53.64, 43.58, 43.24, 42.83, 38.33, 38.19, 38.13, 37.84, 37.62, 37.24, 35.84, 35.33, 29.88, 29.78, 29.66, 27.11, 26.98, 23.72, 23.55, 23.47, 21.74, 21.61, 21.55, 21.53, 21.42, 17.99, 17.94, 17.50, 12.99. ^19^F NMR (471 MHz, [D_3_]acetonitrile: [D]chloroform, 1:1) δ –68.92 (**52**), –73.93 (d, *J* = 708.1 Hz, PF_6_).

**Host–guest complexation experiments:** The solutions for the UV/Vis and fluorescence titration experiments were prepared in a mixture of MeCN and CHCl_3_ (1:1, v/v). The MeCN was freshly distilled prior to use. The anhydrous CHCl_3_ was bought from Acros Organics and filtered through anhydrous K_2_CO_3_ prior to use to prevent protonation of the porphyrin cage compounds during the titrations. The stock solutions of the hosts (ca. 0.1 mM) were prepared by dissolving circa 1–2 mg of the respective compound in the solvent mixture. From this stock solution, three measuring solutions were prepared containing the host in a concentration of 3.0 µM. The guest stock solution (2.0 mM) was prepared by dissolving 14.56 mg of the guest in the solvent mixture. From both stock solutions, two titration solutions were prepared both containing the host at a concentration of 3.0 µM and the guest at a concentration of 0.80 mM and of 80 µM. Prior to the fluorescence titrations, the excitation wavelength (λ ca. 420 nm) was determined by measuring the maximum absorption wavelength in the UV/Vis spectrum. The decays of fluorescence intensities as a result of the addition of small amounts of guests were recorded. The resulting data was uploaded to a web application to fit the data and calculate the association constants.^20^

## Supporting information

Supporting Information
