# Supplementary material for: Effect of Chirality on the Binding of Viologen Guests in Porphyrin Macrocycles
Source: European J Org Chem. 2019 May 3;2019(21):3525–33. doi: 10.1002/ejoc.201900221 (PMC6582502; doi:10.1002/ejoc.201900221)
Supplement: Supplementary file 1 — Supporting Information [file EJOC-2019-3525-s001.pdf]

European Journal of Organic Chemistry

Supporting Information

Effect of Chirality on the Binding of Viologen Guests in Porphyrin Macrocycles

Shaji Varghese, Bram Spierenburg, Jeroen P. J. Bruekers,
Anne Swartjes, Paul B. White, Johannes A. A. W. Elemans,*
and Roeland J. M. Nolte*

ejoc201900221-sup-0001-SupMat.pdf

Table of contents

Table S1 ……………………………………………………………………………………..S5

Table S2 ……………………………………………………………………………………..S6

Table S3 ……………………………………………………………………………………..S8

Table S4 ……………………………………………………………………………………S10

Table S5……………………………………………………………………………………..S11

Figure S1 …………………………………………………………………………………S12

Figure S2 …………………………………………………………………………………S13

Figure S3 …………………………………………………………………………………S14

Figure S4 ………………………………………………………………………………...S15

Figure S5 ………………………………………………………………………………… S16

Figure S6 ………………………………………………………………………………… S17

Figure S7 ………………………………………………………………………………… S18

Figure S8 ………………………………………………………………………………… S19

Figure S9 ………………………………………………………………………………… S20

Figure S10 ……………………………………………………………………………….. S21

Figure S11 ……………………………………………………………………………….. S22

Figure S12 ……………………………………………………………………………….. S23

Figure S13 ……………………………………………………………………………… .S24

Figure S14 ………………………………………………………………………………...S25

Figure S15 ……………………………………………………………………………… .S26

Figure S16 …………………………………………………………………………………… S27

Figure S17 …………………………………………………………………………………… S28

Figure S18 …………………………………………………………………………………… S29

Figure S19 …………………………………………………………………………………… S30

Figure S20 …………………………………………………………………………………… S31

Figure S21 …………………………………………………………………………………… S32

Figure S22 …………………………………………………………………………………… S33

Figure S23 …………………………………………………………………………………… S34

Figure S24 …………………………………………………………………………………… S35

Figure S25 …………………………………………………………………………………… S36

Figure S26……………………………………………………………………………………..S37

Figure S27……………………………………………………………………………………..S38

Figure S28……………………………………………………………………………………..S39

Figure S29……………………………………………………………………………………..S40

Figure S30……………………………………………………………………………………..S41

Figure S31……………………………………………………………………………………..S42

Figure S32……………………………………………………………………………………..S43

Figure S33……………………………………………………………………………………..S44

Figure S34……………………………………………………………………………………..S45

Figure S35……………………………………………………………………………………..S46

Figure S36……………………………………………………………………………………..S47

Figure S37……………………………………………………………………………………..S48

Figure S38…………………………………………………………………………………...S49

Figure S39…………………………………………………………………………………...S50

Figure S40…………………………………………………………………………………...S51

Figure S41…………………………………………………………………………………...S52

Figure S42…………………………………………………………………………………...S53

Figure S43…………………………………………………………………………………...S54

Figure S44…………………………………………………………………………………...S55

Figure S45…………………………………………………………………………………...S56

Figure S46…………………………………………………………………………………...S57

Figure S47…………………………………………………………………………………...S58

Figure S48…………………………………………………………………………………...S59

Figure S49…………………………………………………………………………………...S60

Figure S50…………………………………………………………………………………...S61

Figure S51…………………………………………………………………………………...S62

Figure S52…………………………………………………………………………………...S63

Figure S53…………………………………………………………………………………...S64

Figure S54…………………………………………………………………………………...S65

Figure S55…………………………………………………………………………………...S66

Figure S56…………………………………………………………………………………...S67

Figure S57…………………………………………………………………………………...S68

Figure S58…………………………………………………………………………………...S69

Figure S59…………………………………………………………………………………...S70

Figure S60……………………………………………………………………………………..S71

Figure S61……………………………………………………………………………………..S72

Figure S62……………………………………………………………………………………..S73

Figure S63……………………………………………………………………………………..S74

Figure S64……………………………………………………………………………………..S75

Figure S65……………………………………………………………………………………..S76

Figure S66……………………………………………………………………………………..S77

Figure S67……………………………………………………………………………………..S78

Figure S68……………………………………………………………………………………..S79

Figure S69……………………………………………………………………………………..S80

Figure S70……………………………………………………………………………………..S81

Figure S71……………………………………………………………………………………..S82

Figure S72 ……………………………………………………………………………………. S83

Figure S73………………………………………………………………………………………S84

Figure S74………………………………………………………………………………………S85

Figure S75………………………………………………………………………………………S86

Figure S76………………………………………………………………………………………S87

Figure S77………………………………………………………………………………………S88

Table S1. Selected CISVs for protons in the complexes of (*R*,S_p*)-$\mathbf{H_24}$ and (*R*,R_p*)-$\mathbf{H_25}$ with guest **V1**.[a]

Host	*NH*	*28a*	*28b*	*30*	*49*	*53*
(*R*,S_p*)-$\mathbf{H_24}$	–0.09	–0.21 (I) –0.16 (II)<br>–0.38 (III) –0.11 (IV)	–0.26 (I) –0.24 (II)<br>–1.01 (III) –1.12(IV)	+0.02 (I)<br>–0.18 (III)<br>–0.29 (IV)	+0.24	+0.17
(*R*,R_p*)-$\mathbf{H_25}$	–0.07	–0.47 (I) –0.23 (II)<br>–0.09 (III) –0.38 (IV)	–0.30 (I) –0.44 (II)<br>–0.98 (III) –0.93(IV)	0.00 (II)<br>–0.22 (III)<br>–0.16 (IV)	+0.94	+0.14

[a] The roman numbers in brackets refer to the locations of the protons in the complexes, i.e. below the respective porphyrin meso-phenyl rings.

Table S2. CISVs for protons in complexes between (*R*,S_p*)-**H$_2$4** and the guests (*R*,*R*)-**V2** and (*S*,*S*)-**V2**[a]

Proton	Guest δ [ppm]	(*R*,S_p*)-**H$_2$4** δ [ppm]	(*R*,S_p*)-**H$_2$4** + (*R*,*R*)-**V2** δ [ppm]	CISV [ppm]	(*R*,S_p*)-**H$_2$4** + (*S*,*S*)-**V2** δ [ppm]	CISV [ppm]
Pyr NH		-2.76	-2.81	-0.05	-2.81	-0.05
3, 4	–	8.82, 8.96	9.15, 9.29	+0.33, +0.33	9.13, 9.32	+0.31, +0.36
8, 9		8.78/8.68/8.66/ 8.61	8.86/8.79	~+0.1	8.87, 8.79	~+0.1
13, 14			9.18/9.14	~+0.5	9.21/9.15	~+0.5
18, 19			8.86/8.79	~+0.1	8.87/8.76	~+0.1
27(I)a		4.16	4.16	0	4.16	0
27(II)a		4.14	3.77	-0.37	3.81	-0.33
27(III)a		3.93	4.29	+0.36	4.26	+0.33
27(IV)a		4.06	4.28	+0.22	4.26	+0.20
27(I)b		3.71	4.02	+0.31	4.03	+0.32
27(II)b		3.60	3.58	-0.02	3.64	+0.04
27(III)b		3.83	4.05	+0.22	4.00	+0.17
27(IV)b		3.67	4.05	+0.38	4.02	+0.35
28(I)a		3.65	3.35	-0.30	3.34	-0.31
28(II)a		3.82	3.33	-0.49	3.35	-0.47
28 (III)a		3.70	3.36	-0.34	3.35	-0.35
28(IV)a		3.42	3.36	-0.06	3.35	-0.07
28(I)b		3.22	2.79	-0.43	2.90	-0.32
28(II)b		3.33	3.06	-0.27	3.12	-0.21
28 (III)b		3.22	2.66	-0.56	2.51	-0.71
28(IV)b		3.22	2.46	-0.76	2.31	-0.91
30(I)		6.05	5.88	-0.17	5.93	-0.12
30(III)		6.21	6.07	-0.14	6.04	-0.17
30(IV)		6.22	6.08	-0.14	6.09	-0.13
32(I)a		4.16	4.24	+0.08	4.24	-0.08
32(II)a		4.32	4.27	-0.05	4.28	-0.04
32(III)a		4.26	4.38	+0.12	4.39	+0.13
32(IV)a		4.13	4.24	+0.11	4.26	+0.13
32(I)b		3.72	3.87	+0.15	3.87	+0.15
32(II)b		3.58	3.68	+0.10	3.70	+0.12
32 (III)b		3.80	3.90	+0.10	3.90	+0.10
32(IV)b		3.70	3.83	+0.13	3.84	+0.14
NH 49		8.98	9.05	+0.07	9.29	+0.31
53		3.26	3.34	+0.08	3.28	+0.02
V1a,b in	4.66, 4.61		2.91,2.28	-1.75,-2.33	3.20, 2.41	-1.46, -2.20
V1a,b out	4.66, 4.61		4.15,4.05	-0.51, -0.56	4.36, 4.31	-0.30, -0.30
V2a,b in	2.04, 1.86		0.71, 0.39	-1.33, -1.47	-0.03, 0.47	-2.07, -1.39
V2a,b out	2.04, 1.86		1.41, 1.34	-0.63, -0.52	1.67, 1.51	-0.37, -0.35
V3 in	1.60		1.18	-0.42	1.00	-0.60
V3 out	1.60		1.47	-0.13	1.50	-0.10
V4 in	1.02		0.76	-0.26	0.69	-0.33

V4 out	1.02	0.98	-0.04	1.02	0
V10 in	8.93	5.21	-3.72	4.53	-4.40
V10 out	8.93	7.36	-1.57	7.88	-1.05
V11 in	8.41	3.51	-4.90	3.34	-5.07
V11 out	8.41	4.93	-3.48	5.46	-2.95

[a] Protons indicated with a ‘V’ belong to the guest molecule. A slash indicates that it is uncertain which signal belongs to which proton. A comma is used to indicate that proton signals belong to the respective protons. CISVs provided with an ‘~’ are uncertain due to uncertain proton assignment. The notation ‘in’ refers to the guest proton on the same side as the Mosher amide moiety and the notation ‘out’ to the guest proton on the other side as the Mosher amide moiety.

Table S3. CISVs for protons in complexes between (*R*,*R*$_p$*)-$\mathbf{H_25}$ and guests (*R*,*R*)-**V2** and (*S*,*S*)-**V2**[a]

Proton	Guest	(*R*,*R*$_p$*)-$\mathbf{H_25}$	(*R*,*R*$_p$*)-$\mathbf{H_25}$ + *R*,*R*)-Guest		(*R*,*R*$_p$*)-$\mathbf{H_25}$ + (*S*,*S*)-Guest	
	δ [ppm]	δ [ppm]	δ [ppm]	CISV [ppm]	δ [ppm]	CISV [ppm]
Pyr NH		-2.69	-2.83	-0.14	-2.84	-0.15
3, 4	-----------	8.81-8.70/ 8.65/8.61	9.20/9.03	~+0.5	9.19/9.04	~+ 0.5
8, 9			8.83/8.76	~+0.1	8.83/8.78	~+0.1
13, 14			9.21/9.13	~+0.5	9.19/9.12	~+0.5
18, 19			8.80, 8.90	~+0.1	8.80, 8.91	~+0.1
27(I)a		4.46	4.28	-0.18	4.31	-0.15
27(II)a		4.18	4.16	-0.02	4.18	0
27(III)a		4.12	4.28	+0.16	4.32	+0.20
27(IV)a		4.06	4.26	+0.20	4.32	+0.26
27(I)b		4.05	3.93	-0.12	3.93	-0.12
27(II)b		4.06	4.02	-0.04	4.03	-0.03
27(III)b		4.03	4.01	-0.02	4.05	+0.02
27(IV)b		3.92	4.00	+0.08	4.05	+0.13
28(I)a		3.96	3.35	-0.61	3.38	-0.58
28(II)a		3.66	3.36	-0.30	3.38	-0.28
28 (III)a		3.37	3.34	-0.03	3.38	+0.01
28(IV)a		3.75	3.36	-0.39	3.38	-0.37
28(I)b		3.47	3.18	-0.29	3.18	-0.29
28(II)b		3.31	2.92	-0.39	2.86	-0.45
28 (III)b		3.18	2.35	-0.83	2.52	-0.66
28(IV)b		3.21	2.52	-0.69	2.68	-0.53
30(II)		6.11	5.96	-0.15	5.96	-0.15
30(III)		6.18	6.07	-0.11	6.10	-0.08
30(IV)		6.22	6.04	-0.18	6.11	-0.11
32(I)a		3.67	4.27	+0.60	4.25	+0.58
32(II)a		4.08	4.25	+0.17	4.26	+0.18
32(III)a		4.12	4.26	+0.14	4.26	+0.14
32(IV)a		4.21	4.38	+0.17	4.39	+0.18
32(I)b		3.28	3.75	+0.47	3.72	+0.44
32(II)b		3.67	3.87	+0.20	3.86	+0.19
32 (III)b		3.66	3.83	+0.17	3.82	+0.16
32(IV)b		3.68	3.89	+0.21	3.88	+0.20
NH 49		8.07	9.31	+1.24	9.18	+1.11
53		3.22	3.27	+0.05	3.29	+0.07
V1a,b in	4.66, 4.61		3.44, 2.66	-1.22, -1.95	3.09, 3.56	-1.57, -1.05
V1a,b out	4.66, 4.61		4.22, 4.17	-0.44, -0.44	4.03, 3.94	-0.63, -0.67
V2a,b in	2.04, 1.86		0.10, 0.62	-1.94, -1.24	0.90, 0.49	-1.14, -1.37
V2a,b out	2.04, 1.86		1.59, 1.45	-0.45, -0.41	1.35, 1.29	-0.69, -0.57
V3 in	1.60		1.17	-0.43	1.32	-0.28
V3 out	1.60		1.50	-0.10	1.47	-0.13
V4 in	1.02		0.86	-0.16	0.84	-0.18

V4 out	1.02	1.00	-0.02	0.98	-0.04
V10 in	8.93	4.94	-3.99	5.52	-3.41
V10 out	8.93	7.63	-1.30	7.17	-1.76
V11 in	8.41	3.37	-5.04	3.69	-4.72
V11 out	8.41	5.14	-3.27	4.76	-3.65

[a] Protons indicated with a ‘V’ belong to the guest molecule. A slash indicates that it is uncertain which signal belongs to which proton. A comma is used to indicate that proton signals belong to the respective protons. CISVs provided with an ‘~’ are uncertain due to uncertain proton assignment. The notation ‘in’ refers to the guest proton on the same side as the Mosher amide moiety and the notation ‘out’ to the guest proton on the other side as the Mosher amide moiety.

Table S4. Association constants of complexes between (R,S_p*)-**H₂4** and **V1** and **V2**.

Host	Guest	K [M^{-1}]	Fit error [%]	URL
(R,S_p*)-**$H_2$4**	**V1**	9.78 * 10^5	6	http://app.supramolecular.org/bindfit/view/1e95e65f-a5a7-48c2-b1a9-b2228b41d174
(R,S_p*)-**$H_2$4**	**V1**	8.02 * 10^5	1	http://app.supramolecular.org/bindfit/view/08e82184-b858-491e-bced-90892ab9f0c5
(R,S_p*)-**$H_2$4**	**V1**	8.69 * 10^5	2	http://app.supramolecular.org/bindfit/view/8c3ca7c7-f3df-4fec-9850-4feee77a54eb
Average for (R,S_p*)-**$H_2$4**	**V1**	8.83 * 10^5 ± 8.9 * 10^4		

Host	Guest	K [M^{-1}]	Fit error [%]	URL
(R,S_p*)-**$H_2$4**	(R,R)-**V2**	1.337 * 10^6	6	http://app.supramolecular.org/bindfit/view/5d2c0483-2c08-42e7-aaad-75185712a5d4
(R,S_p*)-**$H_2$4**	(R,R)-**V2**	1.412 * 10^6	8	http://app.supramolecular.org/bindfit/view/ed8296fb-4279-433e-b046-59aee7708e04
(R,S_p*)-**$H_2$4**	(R,R)-**V2**	1.418 * 10^6	6	http://app.supramolecular.org/bindfit/view/a8996a07-84d2-461e-a8f1-0d4701a0430d
Average for (R,S_p*)-**$H_2$4**	(R,R)-**V2**	1.388 * 10^6 ± 4.5 * 10^4		

Host	Guest	K [M^{-1}]	Fit error [%]	URL
(R,S_p*)-**$H_2$4**	(S,S)-**V2**	3.73 * 10^6	17	http://app.supramolecular.org/bindfit/view/759b066d-3afc-4f9b-bb86-fae00abd3a83
(R,S_p*)-**$H_2$4**	(S,S)-**V2**	3.73 * 10^6	20	http://app.supramolecular.org/bindfit/view/c841de76-bd7b-4df2-a311-e68cfc35113b
(R,S_p*)-**$H_2$4**	(S,S)-**V2**	4.18 * 10^6	26	http://app.supramolecular.org/bindfit/view/673e6274-850b-44b5-a559-88028cff955e
Average for (R,S_p*)-**$H_2$4**	(S,S)-**V2**	3.88 * 10^6 ± 2.6 * 10^5		

Table S5. Association constants of complexes between (*R*,R_p*)-**$H_2$5** and guests **V1** and **V2**.[a]

Host	Guest	K [M^{-1}]	Fit error [%]	URL
(*R*,R_p*)-**$H_2$5**	**V1**	$1.04 * 10^6$	5	http://app.supramolecular.org/bindfit/view/18219a74-b104-45f6-ba76-f56b82aaa112
(*R*,R_p*)-**$H_2$5**	**V1**	$1.42 * 10^6$	3	http://app.supramolecular.org/bindfit/view/4bf485eb-5007-4ee4-a14f-6e37d768be94
(*R*,R_p*)-**$H_2$5**	**V1**	$1.40 * 10^6$	3	http://app.supramolecular.org/bindfit/view/23f40633-656c-4061-a238-d2ec73924645
Average for (*R*,R_p*)-**$H_2$5**	**V1**	$1.29 * 10^6 \pm 2.1 * 10^5$		

Host	Guest	K [M^{-1}]	Fit error [%]	URL
(*R*,R_p*)-**$H_2$5**	(*R*,*R*)-**V2**	$7.1 * 10^6$	18	http://app.supramolecular.org/bindfit/view/3155f464-3fc5-4cd0-af0e-160047e1ff64
(*R*,R_p*)-**$H_2$5**	(*R*,*R*)-**V2**	$2.1 * 10^7$	66	http://app.supramolecular.org/bindfit/view/ecd71a2f-3a57-4ce0-ae38-57ae4abdd443
(*R*,R_p*)-**$H_2$5**	(*R*,*R*)-**V2**	$9.0 * 10^6$	34	http://app.supramolecular.org/bindfit/view/59fc8613-9b40-4c43-979d-1afd74c9fd8f
Average for	(*R*,*R*)-**V2**	$8.0 * 10^6 \pm 1.4 * 10^6$		

Host	Guest	K [M^{-1}]	Fit error [%]	URL
(*R*,R_p*)-**$H_2$5**	(*S*,*S*)-**V2**	$7.52 * 10^6$	20	http://app.supramolecular.org/bindfit/view/d16144c9-2580-432d-9f0e-9ff4ab746d24
(*R*,R_p*)-**$H_2$5**	(*S*,*S*)-**V2**	$7.00 * 10^6$	23	http://app.supramolecular.org/bindfit/view/0316ba92-bef1-422c-86b8-d27631843a96
(*R*,R_p*)-**$H_2$5**	(*S*,*S*)-**V2**	$1.4 * 10^7$	63	http://app.supramolecular.org/bindfit/view/3945be8a-cced-4099-b6e0-5d17c9a46187
Average for (*R*,R_p*)-**$H_2$5**	(*S*,*S*)-**V2**	$7.3 * 10^6 \pm 3.7 * 10^5$		

[a] Data points with a too large fitting error (66 and 63 %) were omitted in the calculation of the final association constants

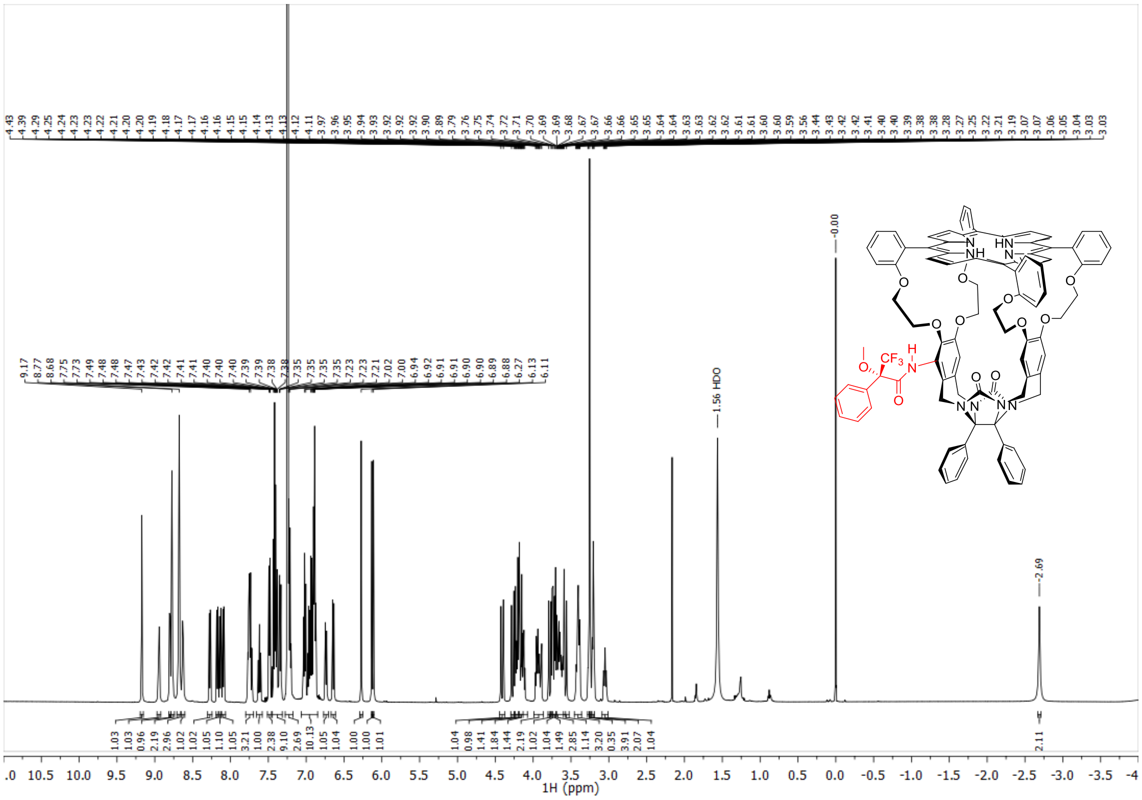

Figure S1. ^{1}H NMR spectrum (500 MHz, 298 K) of (R,S_p*)-$\mathbf{H_24}$ in $CDCl_3$.

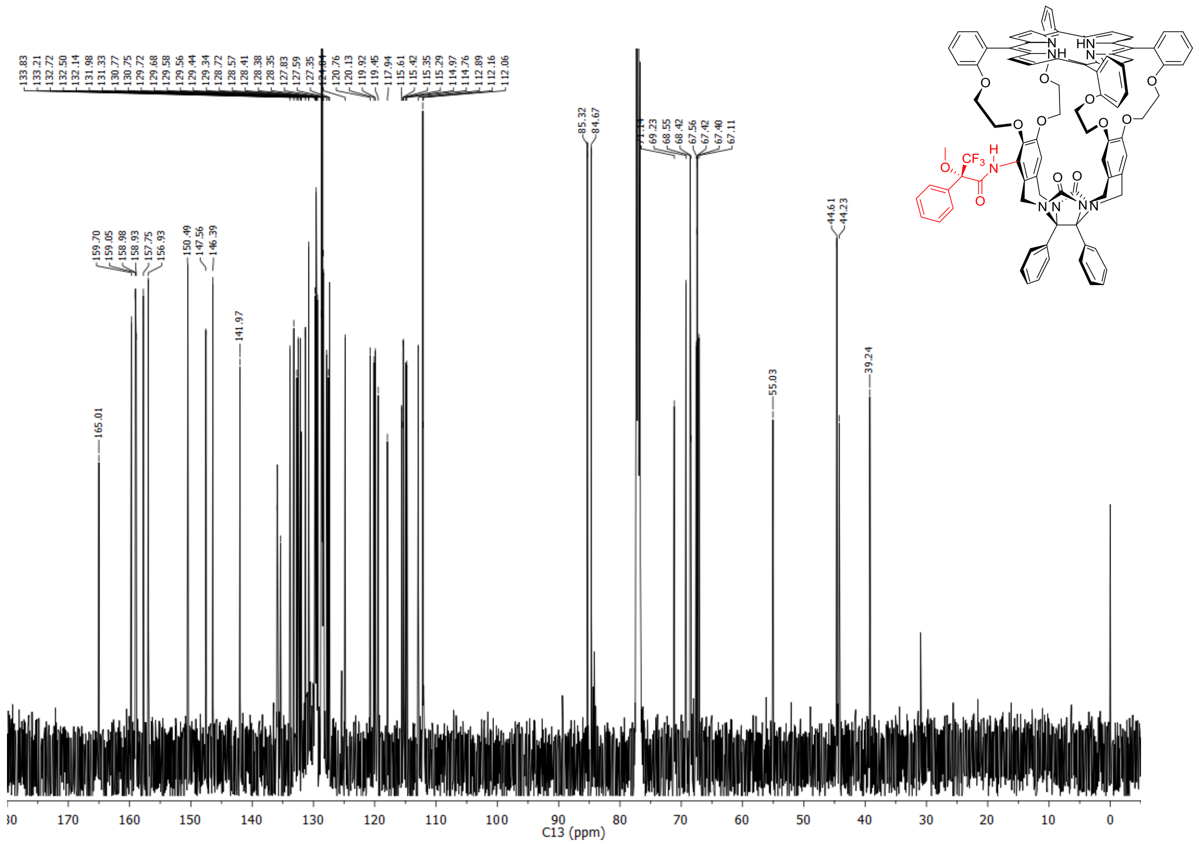

Figure S2. $^{13}C\{^{1}H\}$ NMR spectrum (126 MHz, 298 K) of (*R*,*S*$_p$*)-**H$_2$4** in $CDCl_3$.

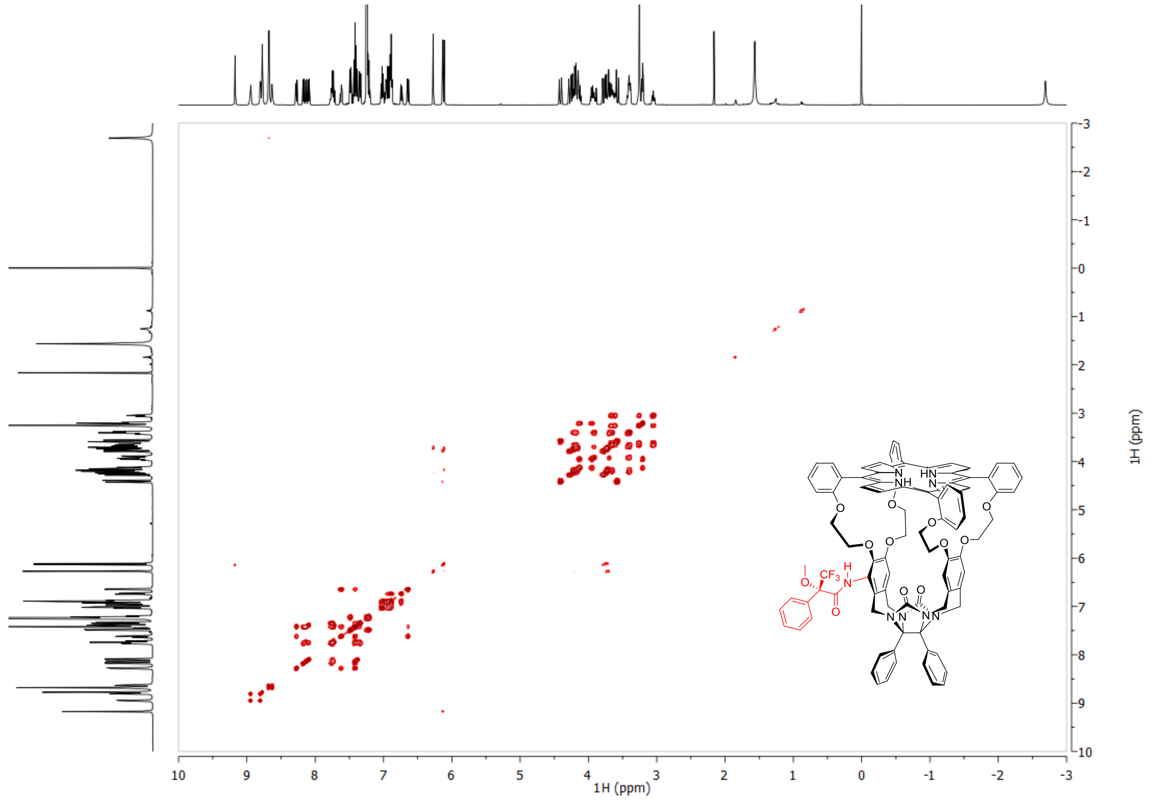

Figure S3. ^{1}H-^{1}H DQF-COSY NMR spectrum (500 MHz, 298 K) of (*R*,*S*$_p$*)-**H$_2$4** in CDCl$_3$.

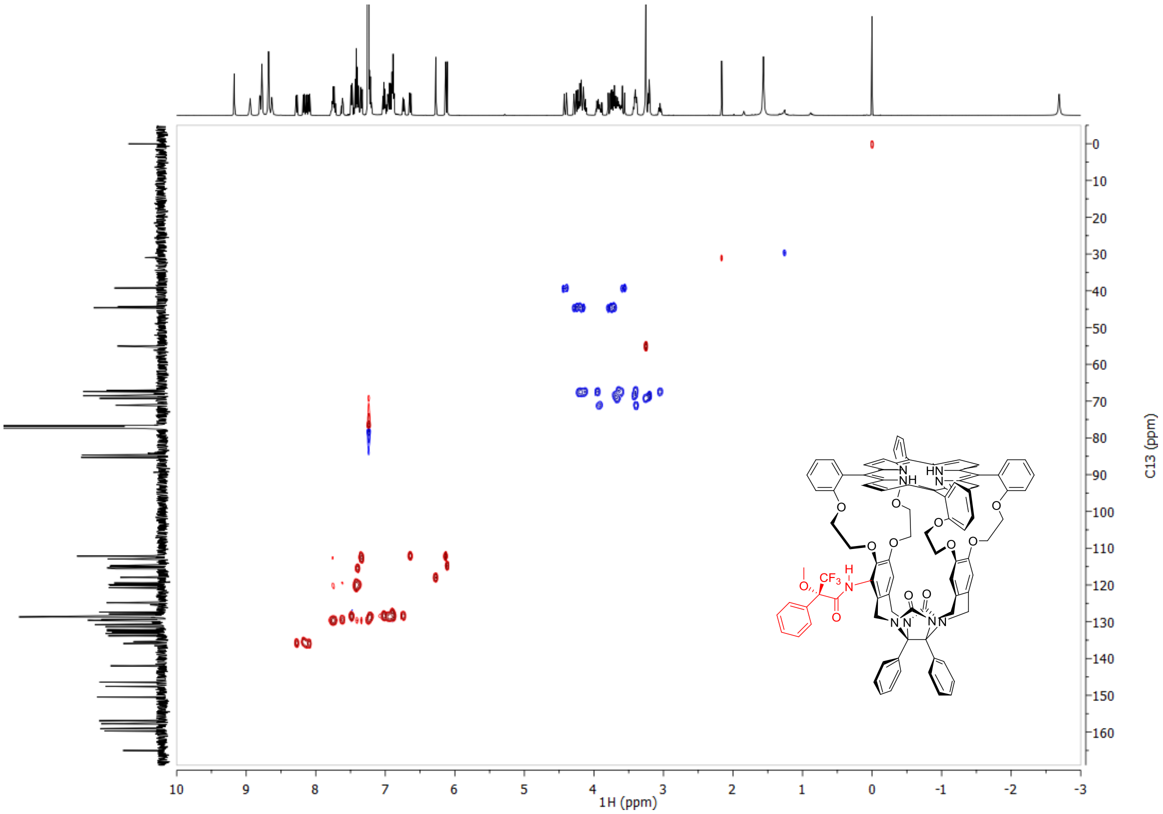

Figure S4. ^{1}H-^{13}C edited HSQC NMR spectrum (500 MHz, 298 K) of (R,S_p*)-**$H_2$4** in $CDCl_3$. CH_2 groups are indicated in blue and CH/CH_3 groups in red.

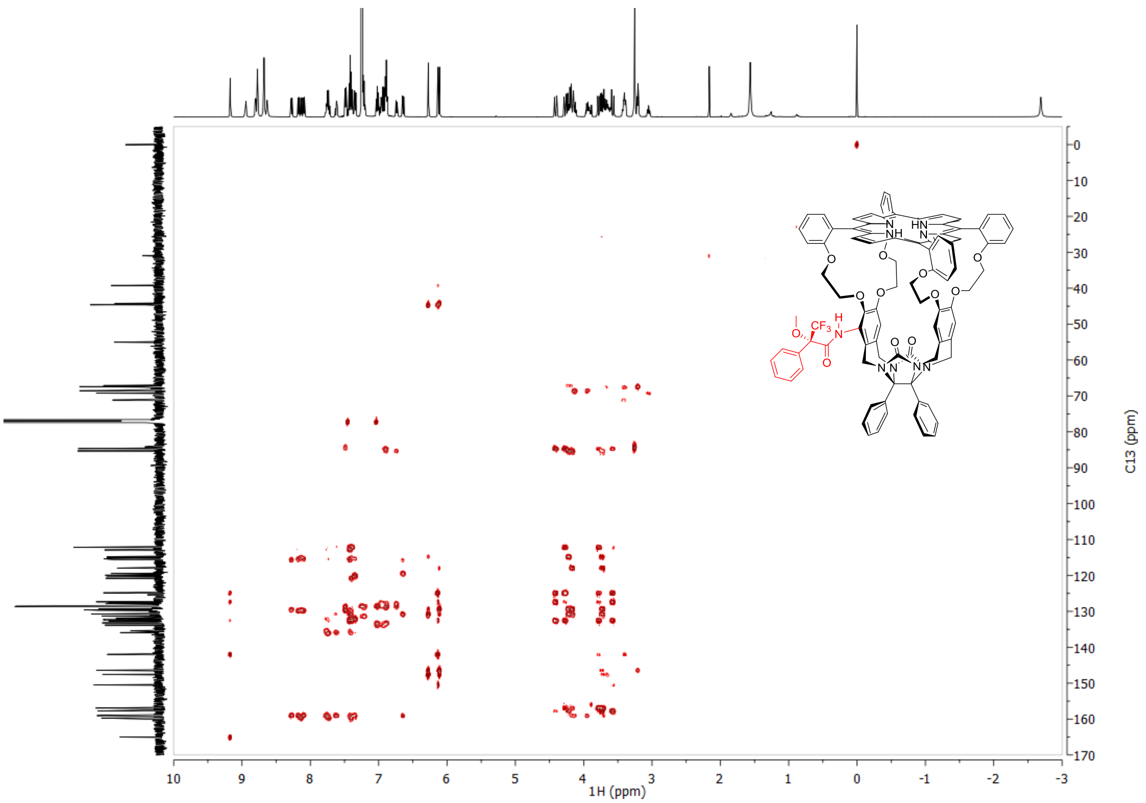

Figure S5. ^{1}H-^{13}C HMBC NMR spectrum (500 MHz, 298 K) of (*R*,*S*$_p$*)-**H$_2$4** in $CDCl_3$.

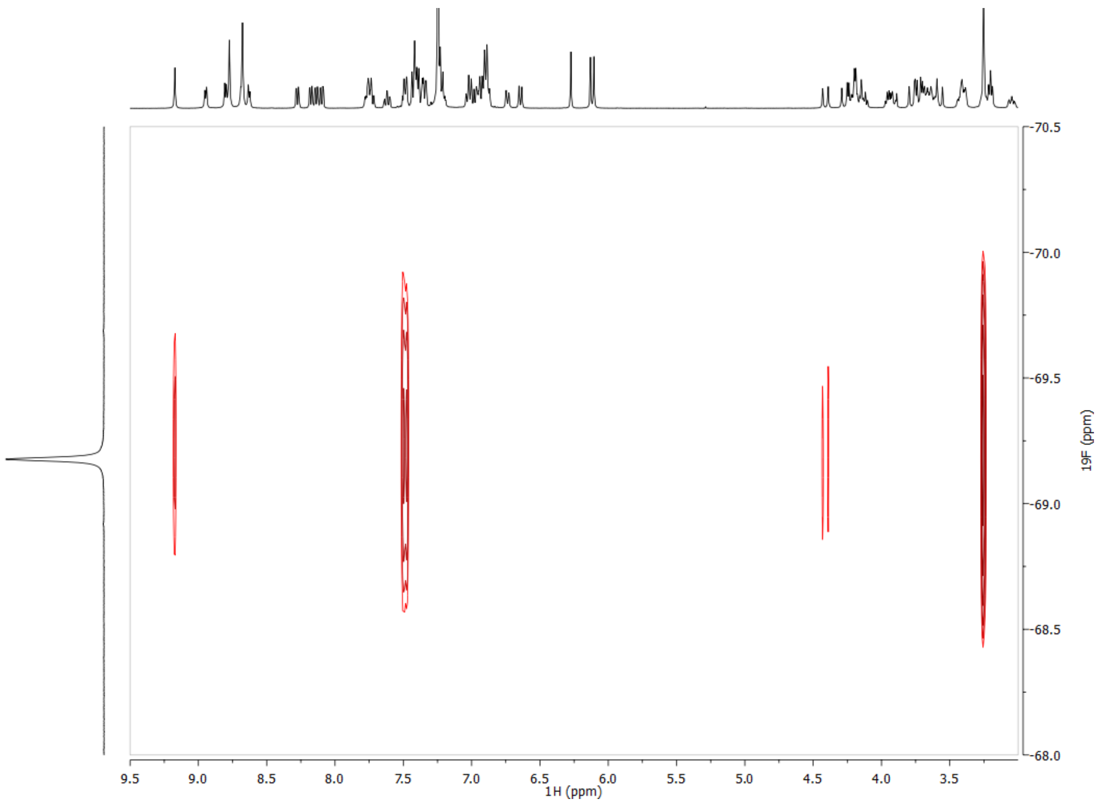

Figure S6. ^{1}H-^{19}F HOESY NMR spectrum (500 MHz, 298 K) of (*R,S*$_p$*)-**H$_2$4** in $CDCl_3$.

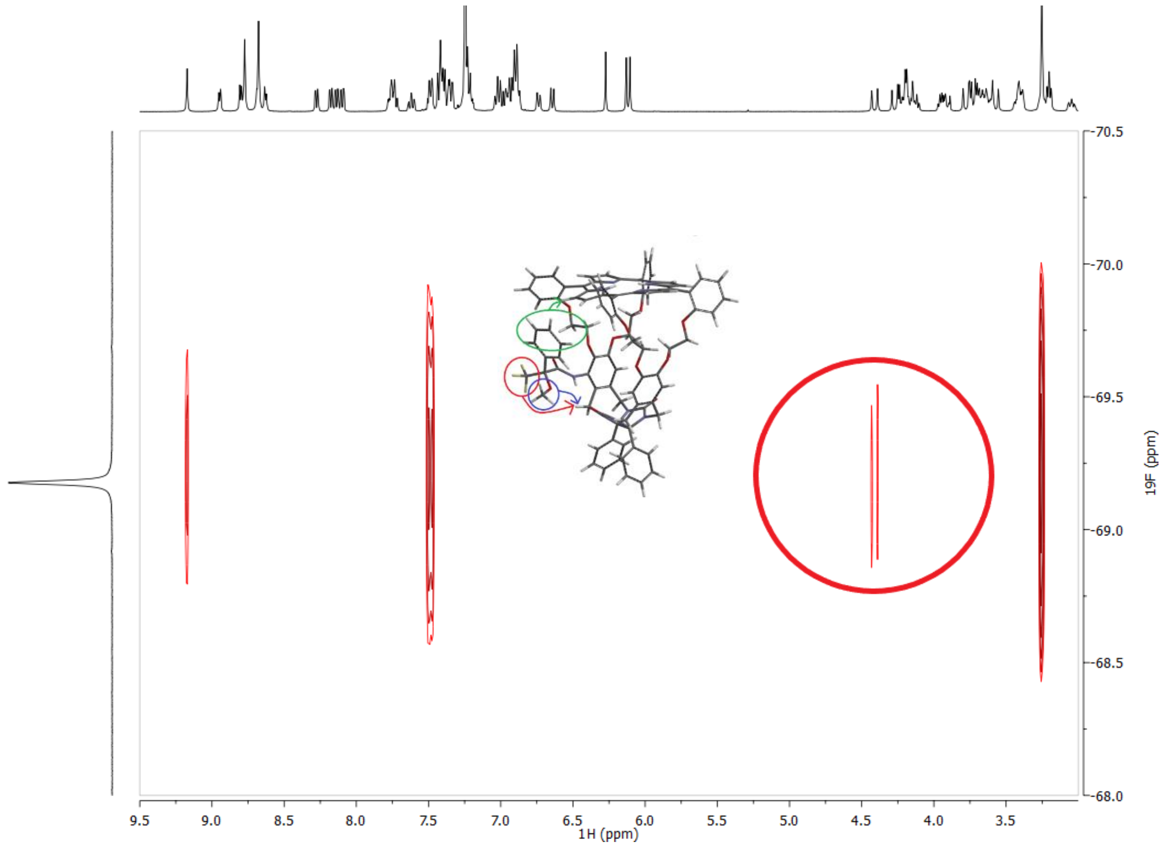

Figure S7. ^{1}H-^{19}F HOESY NMR spectrum (500 MHz, 298 K) of (*R*,S_p*)-$\mathbf{H_24}$ in $CDCl_3$. (Key nOe contacts are encircled).

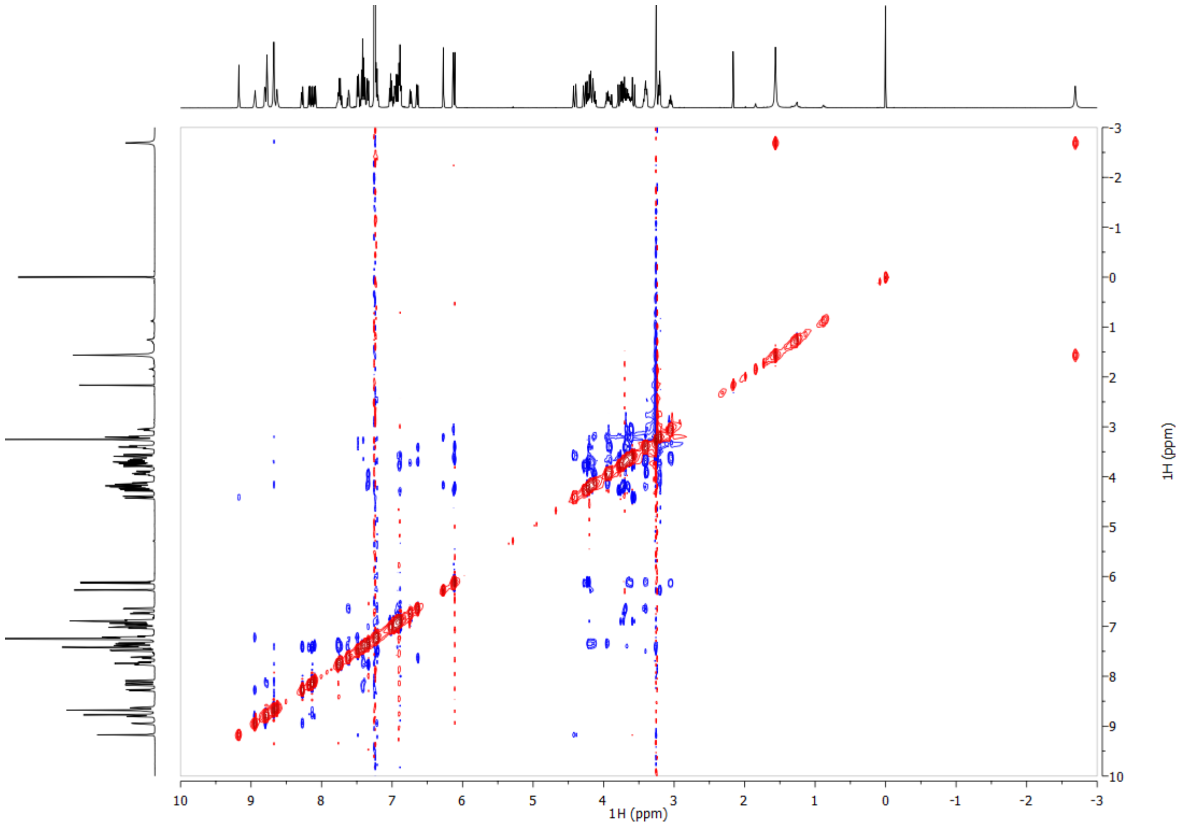

Figure S8. ^{1}H-^{1}H ROESY NMR spectrum (500 MHz, 298 K) of (*R*,*S*$_p$*)-**H$_2$4** in $CDCl_3$.

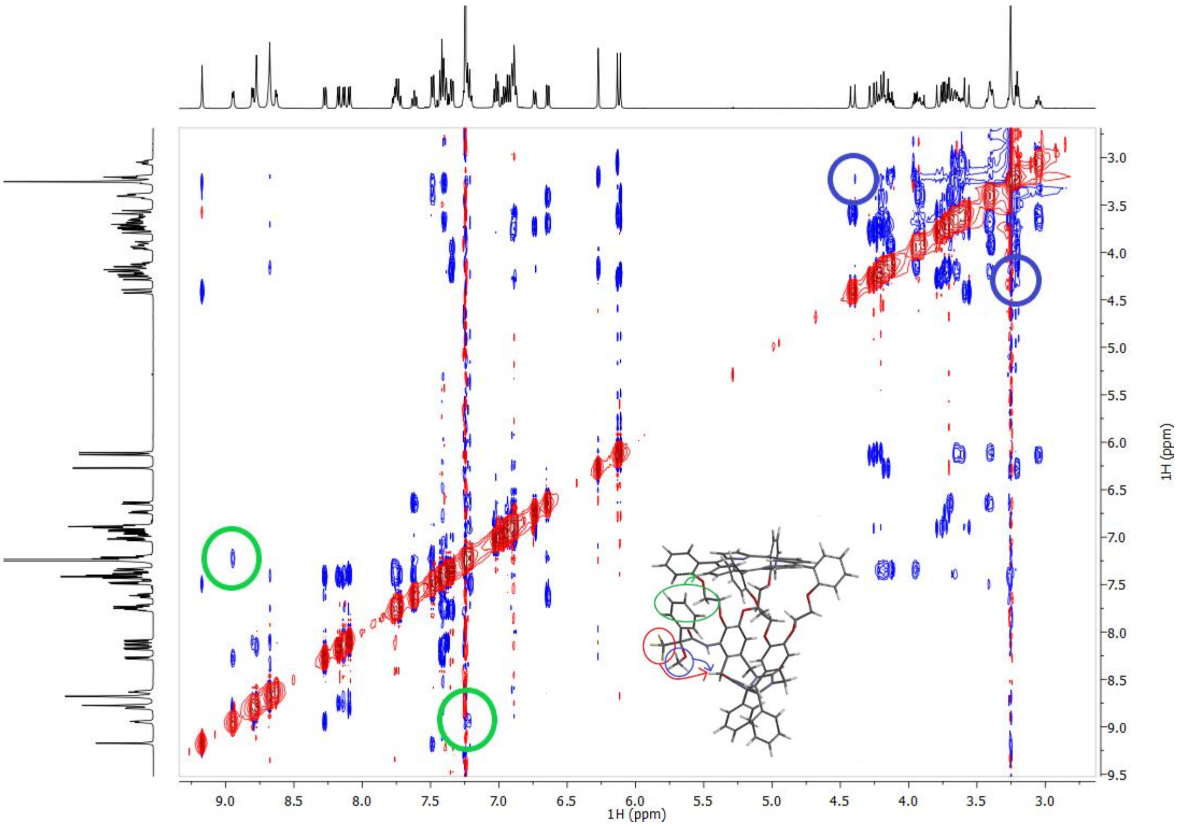

Figure S9. ^{1}H-^{1}H ROESY NMR spectrum (500 MHz, 298 K) of (*R,S$_p$**)-**H$_2$4** in $CDCl_3$. (Key nOe contacts encircled).

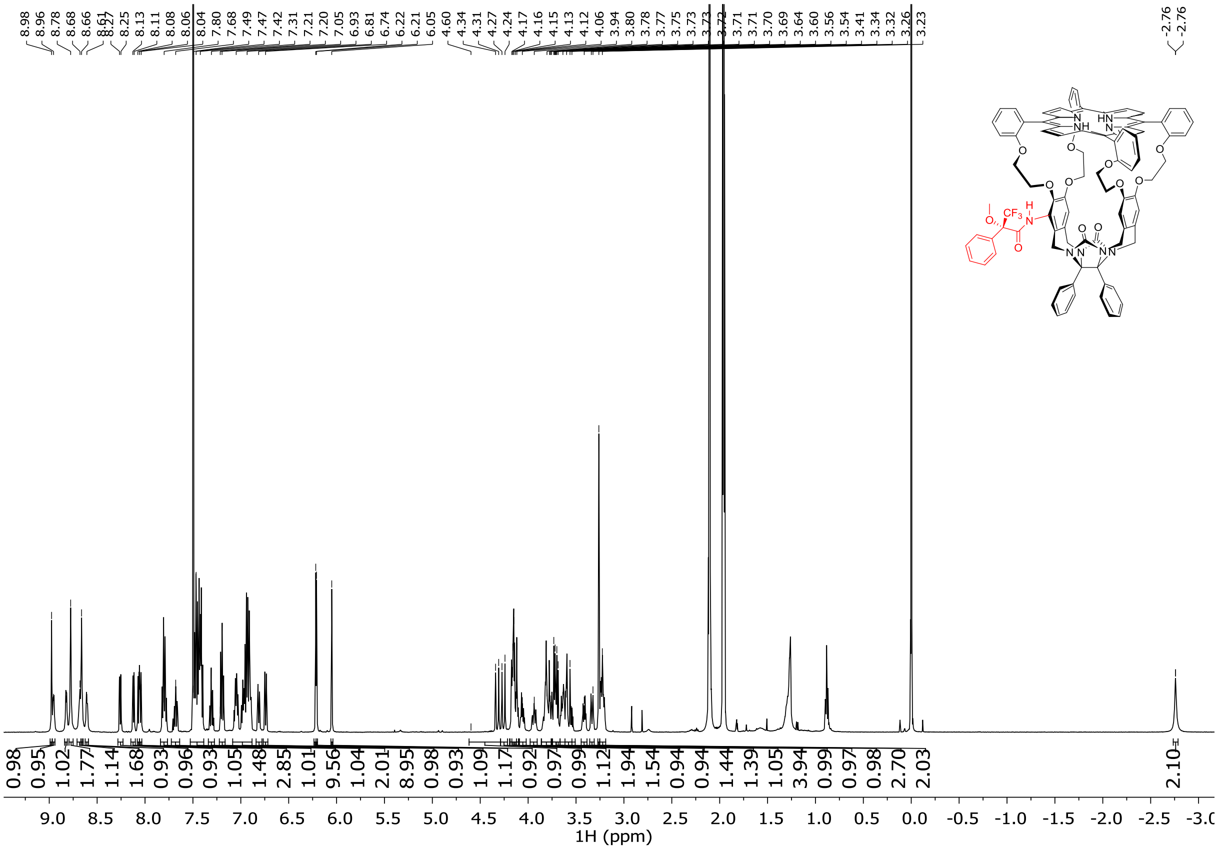

Figure S10. ^{1}H NMR spectrum (500 MHz, 298 K) of the (*R,S**)-**H$_2$4** in $CDCl_3$: D_3CCN, (1:1 v/v).

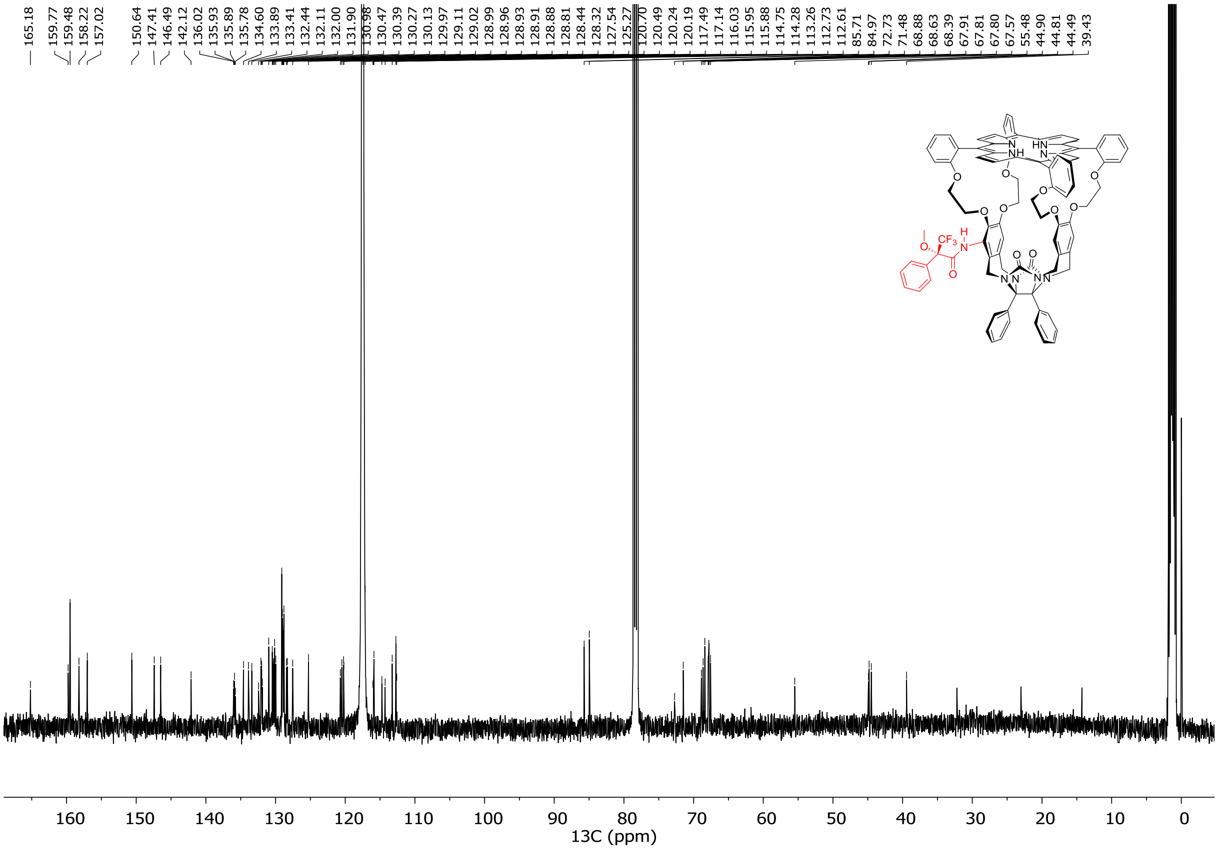

Figure S11. $^{13}C\{^{1}H\}$ NMR spectrum (126 MHz, 298 K) of (*R,S**)-$\mathbf{H_24}$ in $CDCl_3$: D_3CCN, (1:1 v/v).

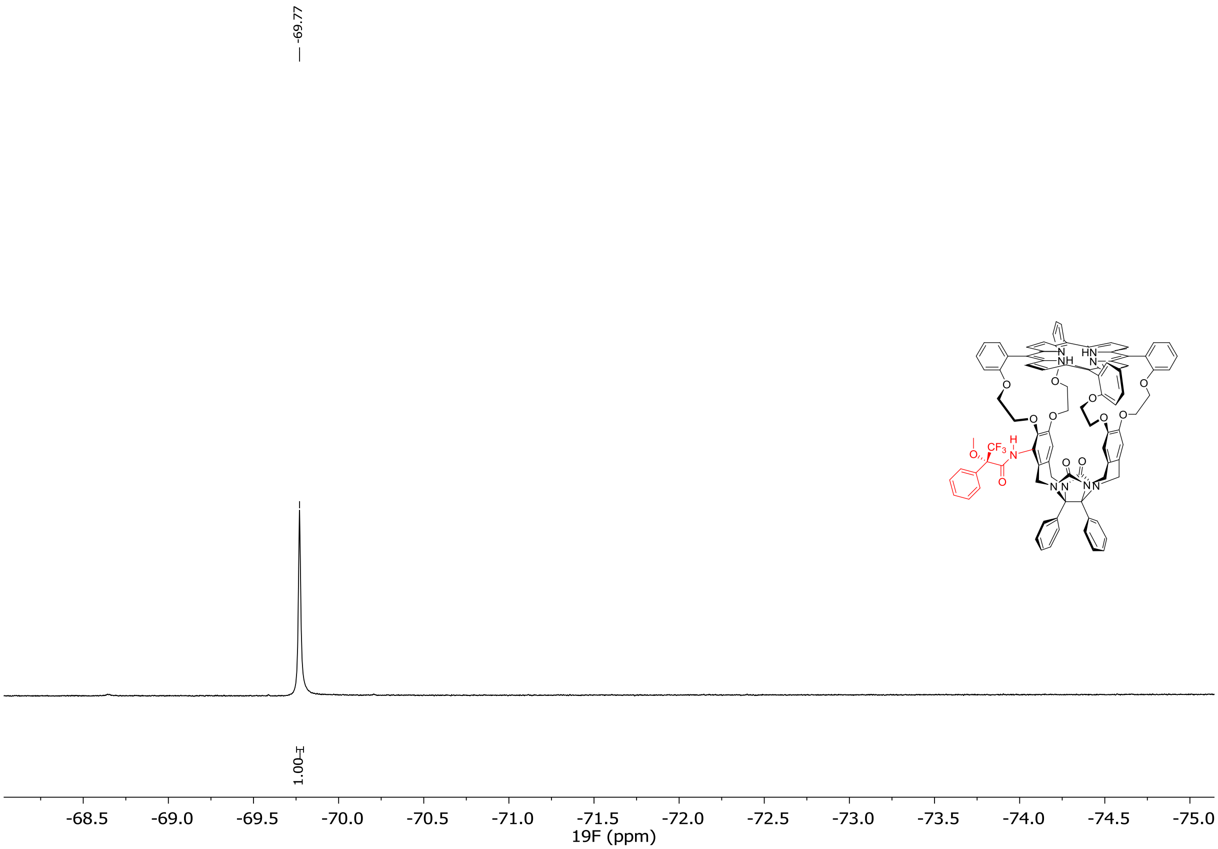

Figure S12. ^{19}F NMR spectrum (471 MHz, 298 K) of (*R,S**)-$\mathbf{H_24}$ in $CDCl_3$: D_3CCN, (1:1 v/v).

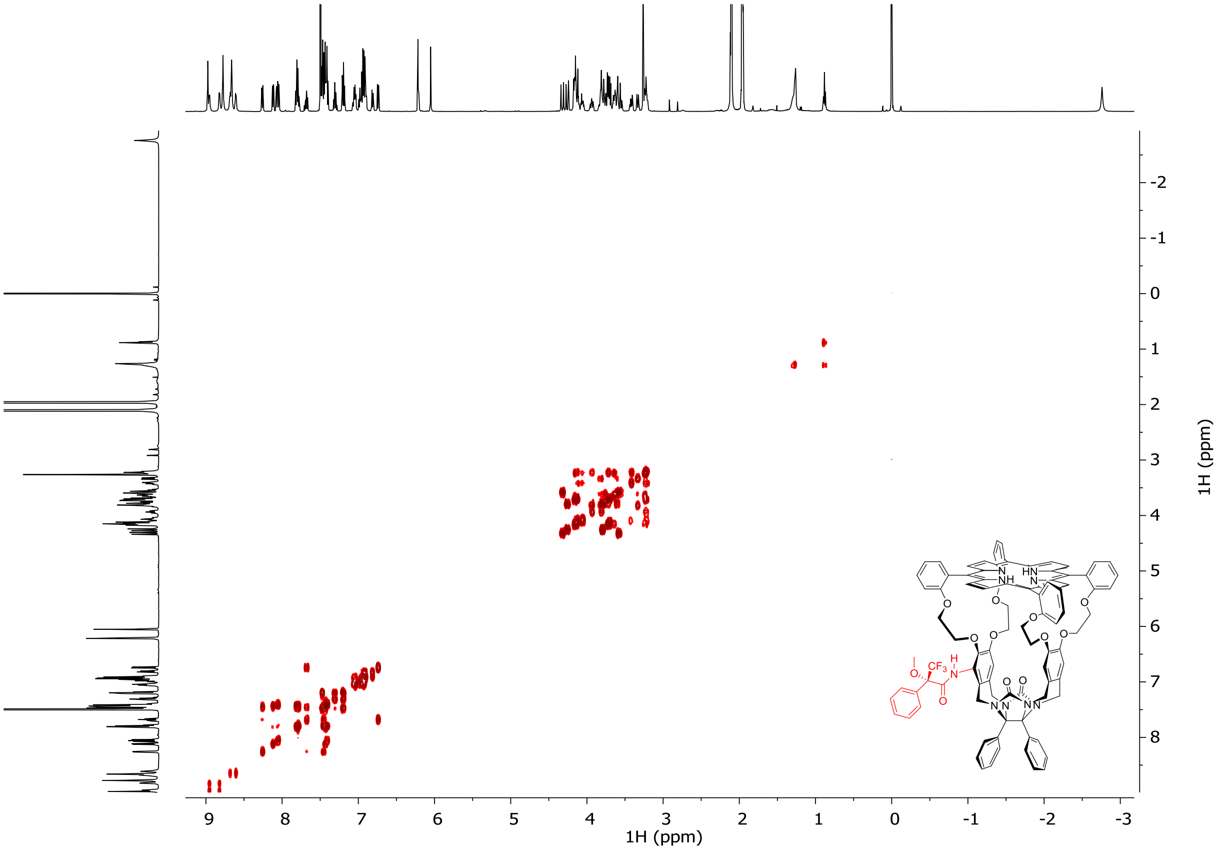

Figure S13. ^{1}H-^{1}H DQF-COSY NMR spectrum (500 MHz, 298 K) of (*R*,*S**)-**$H_2$4** in $CDCl_3$: D_3CCN, (1:1 v/v).

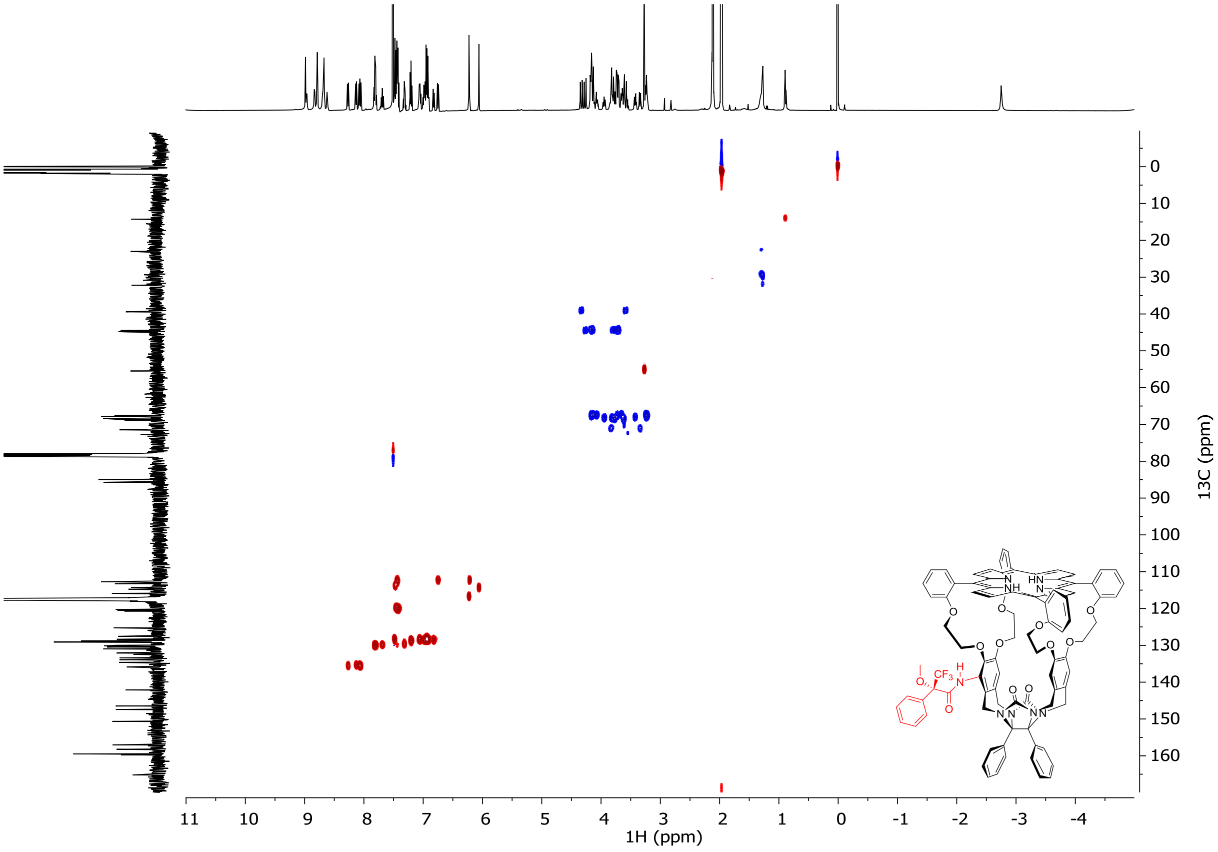

Figure S14. ^{1}H-^{13}C edited HSQC NMR spectrum (500 MHz, 298 K) of (*R*,*S**)-**$H_2$4** in $CDCl_3$: D_3CCN, (1:1 v/v). CH_2 groups are indicated in blue and CH/CH_3 groups in red.

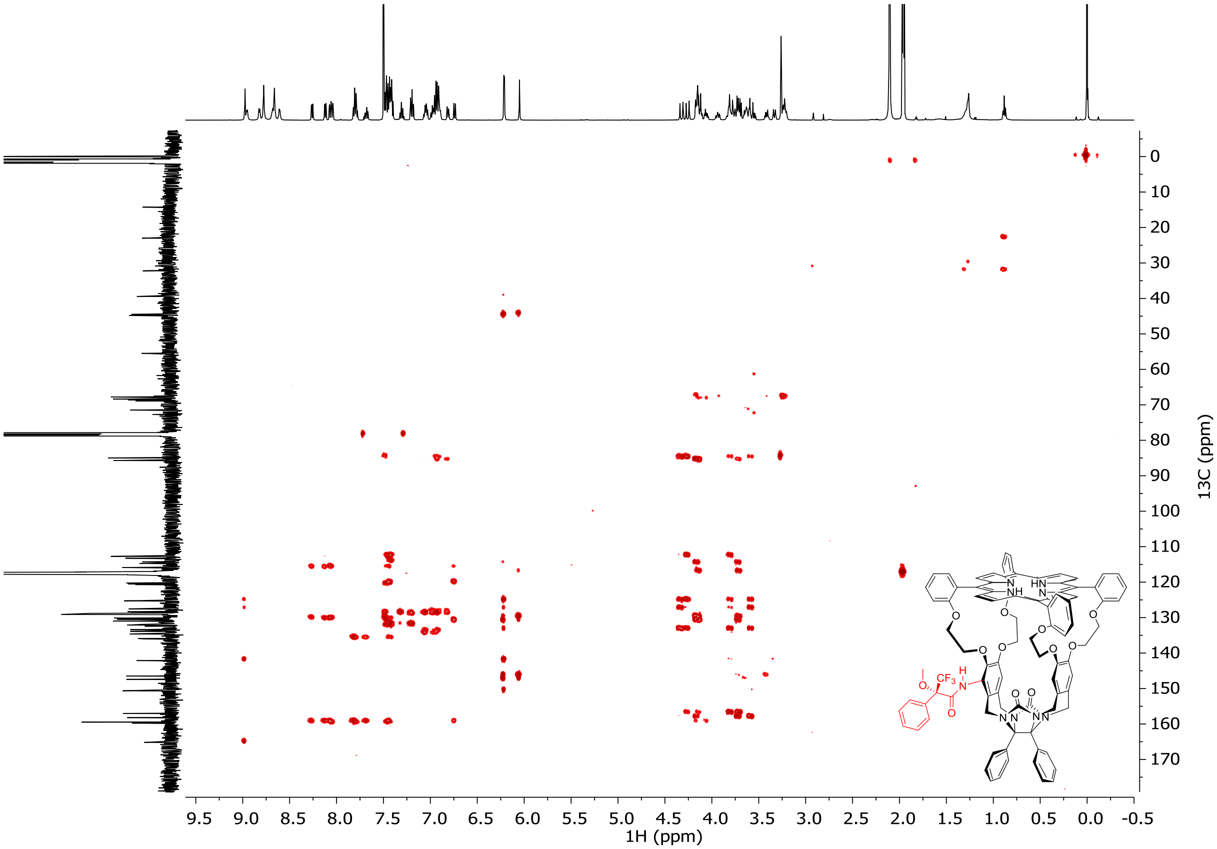

Figure S15. ^{1}H-^{13}C HMBC NMR spectrum (500 MHz, 298 K) of (*R,S**)-**$H_2$4** in $CDCl_3$: D_3CCN, (1:1 v/v).

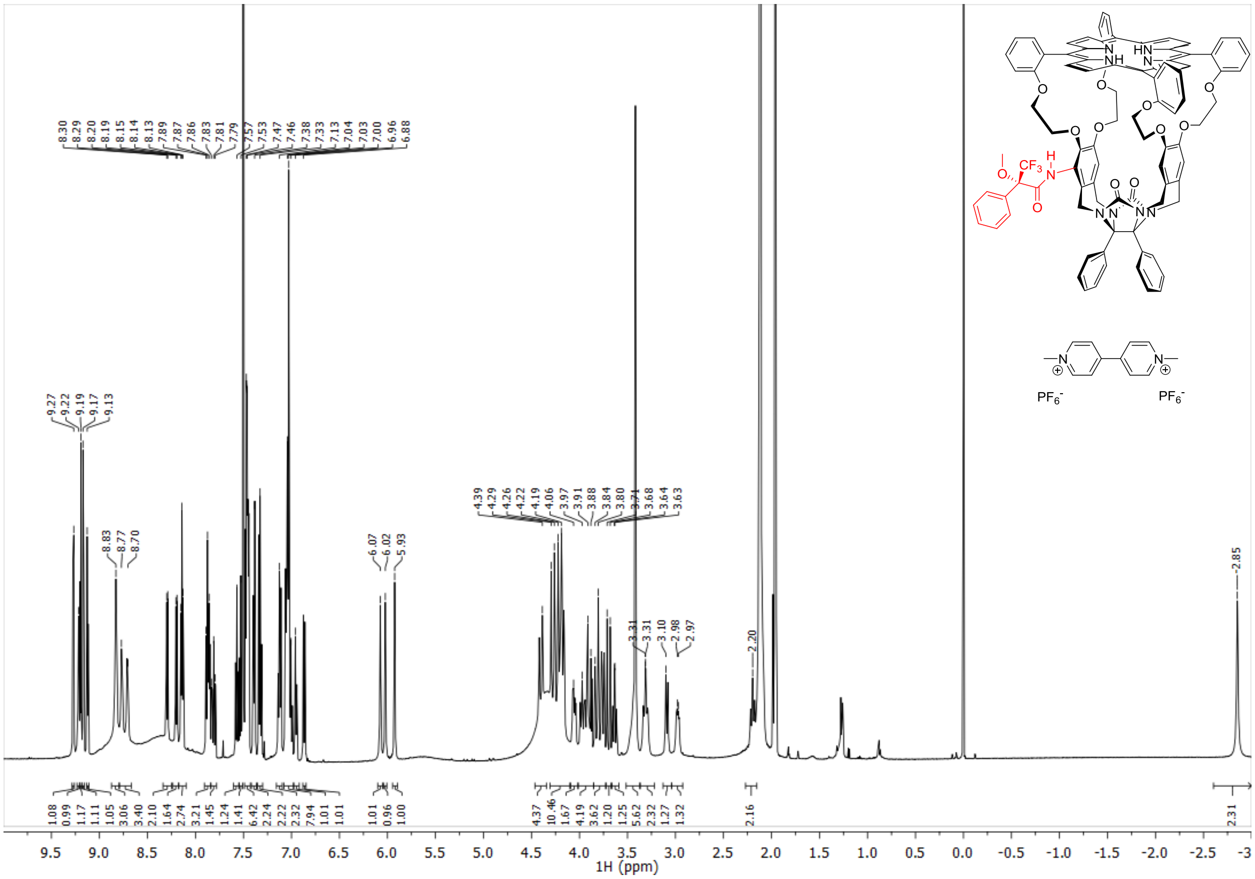

Figure S16. ^{1}H NMR spectrum (500 MHz, 298 K) of the (*R,S**)-**$H_2$4** complex with **V1** in $CDCl_3$: D_3CCN, (1:1 v/v).

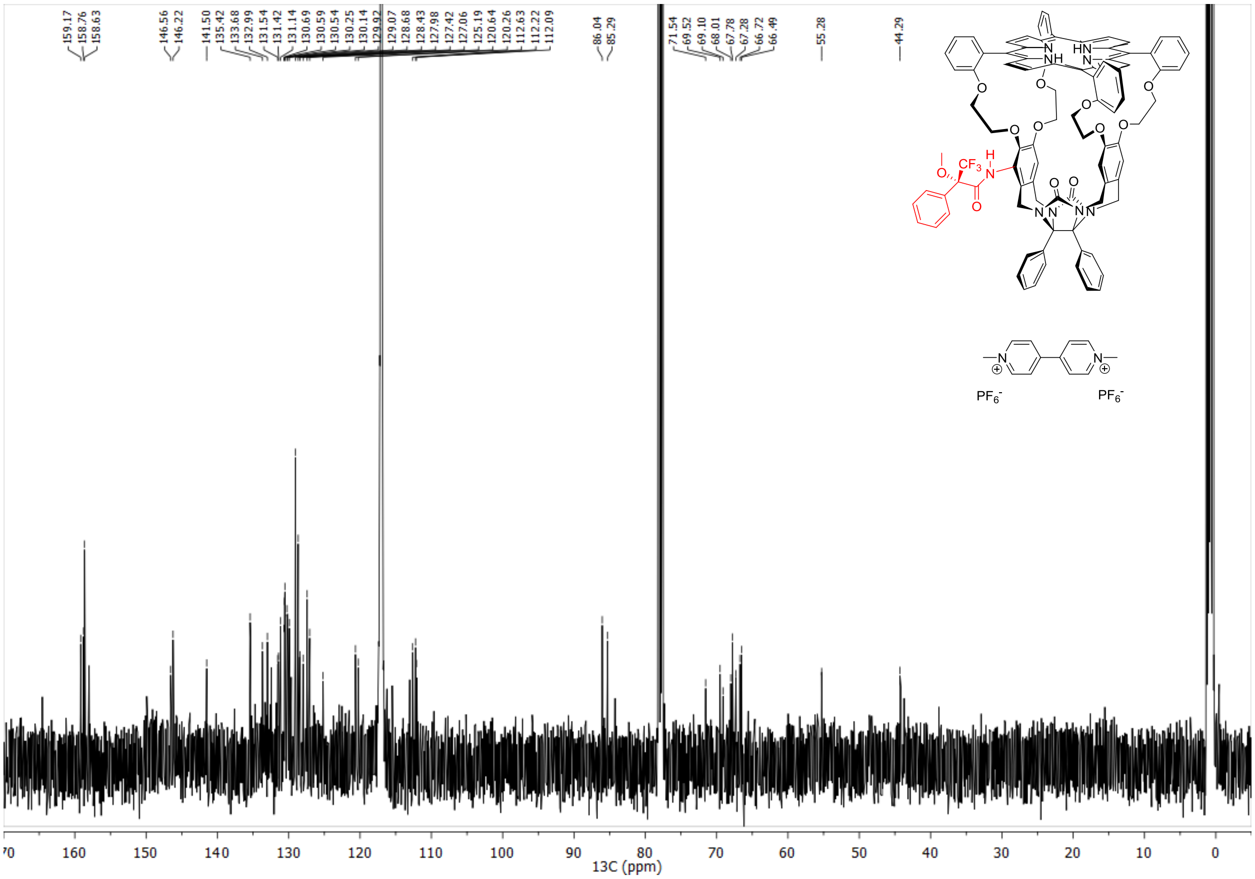

Figure S17. $^{13}C\{^{1}H\}$ NMR spectrum (126 MHz, 298 K) of the (*R,S**)-**$H_2$4** complex with **V1** in $CDCl_3$: D_3CCN, (1:1 v/v).

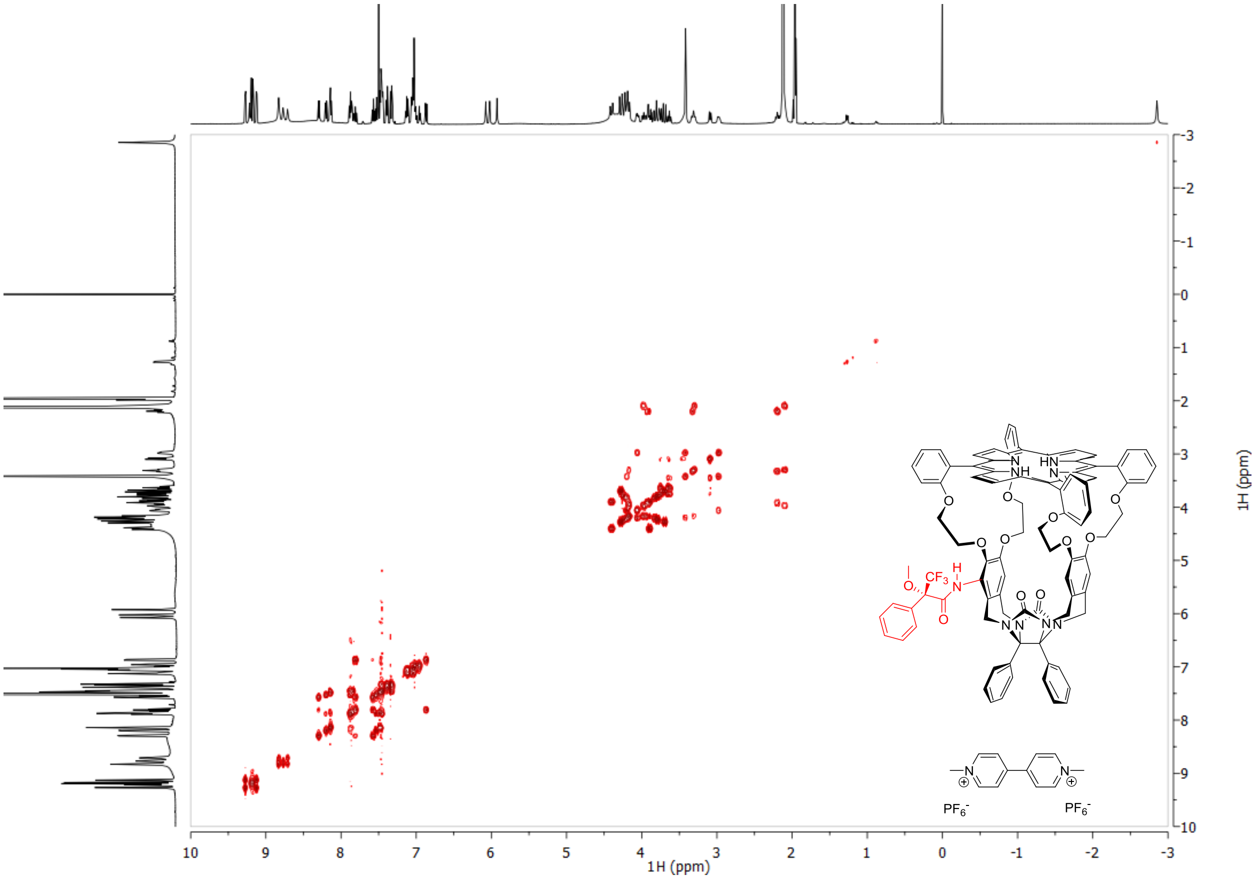

Figure S18. ^{1}H-^{1}H DQF-COSY NMR spectrum (500 MHz, 298 K) of the (*R,S**)-**$H_2$4** complex with **V1** in $CDCl_3$: D_3CCN, (1:1 v/v).

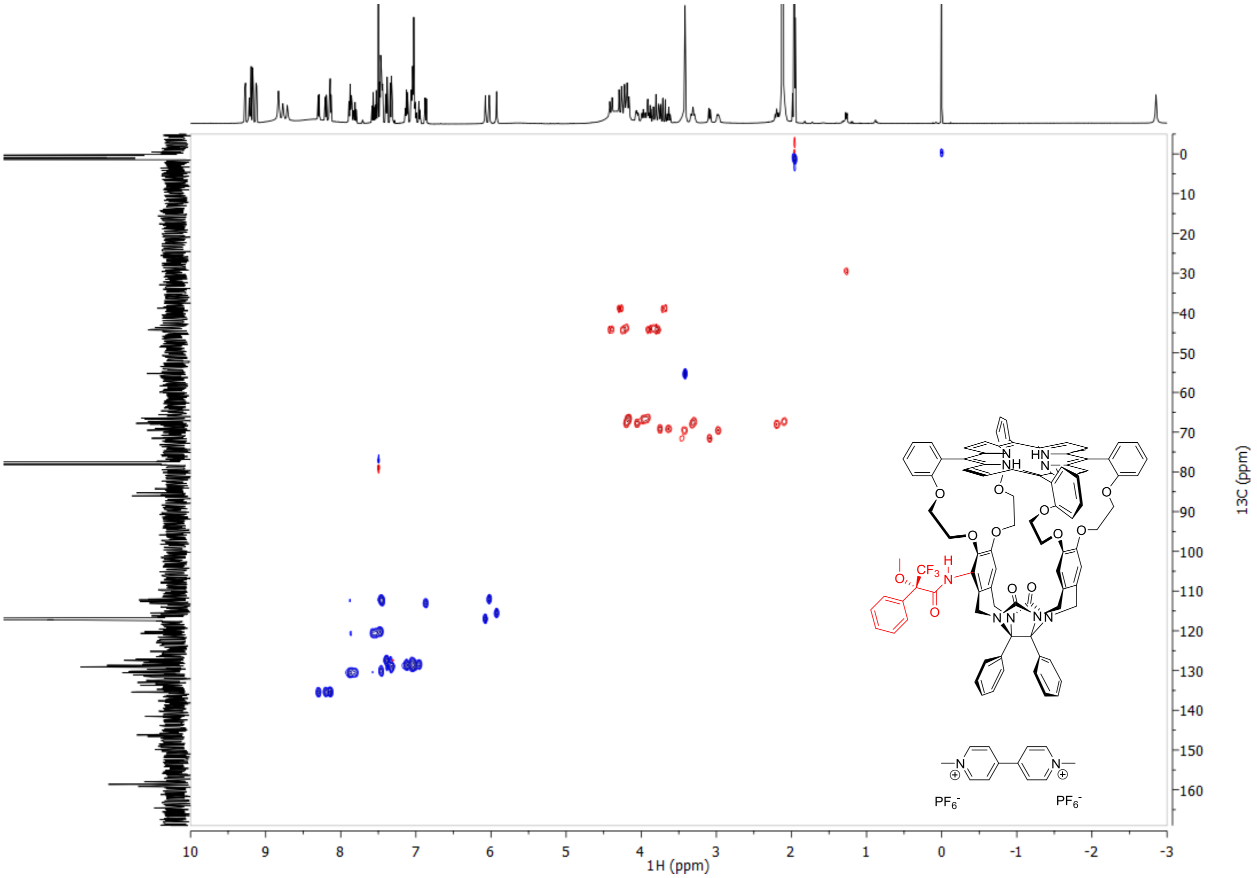

Figure S19. ^{1}H-^{13}C HSQC edited NMR spectrum (500 MHz, 298 K) of the (*R,S**)-$\mathbf{H_2 4}$ complex with **V1** in $CDCl_3$: D_3CCN, (1:1 v/v). CH_2 groups are indicated in blue and CH/CH_3 groups in red.

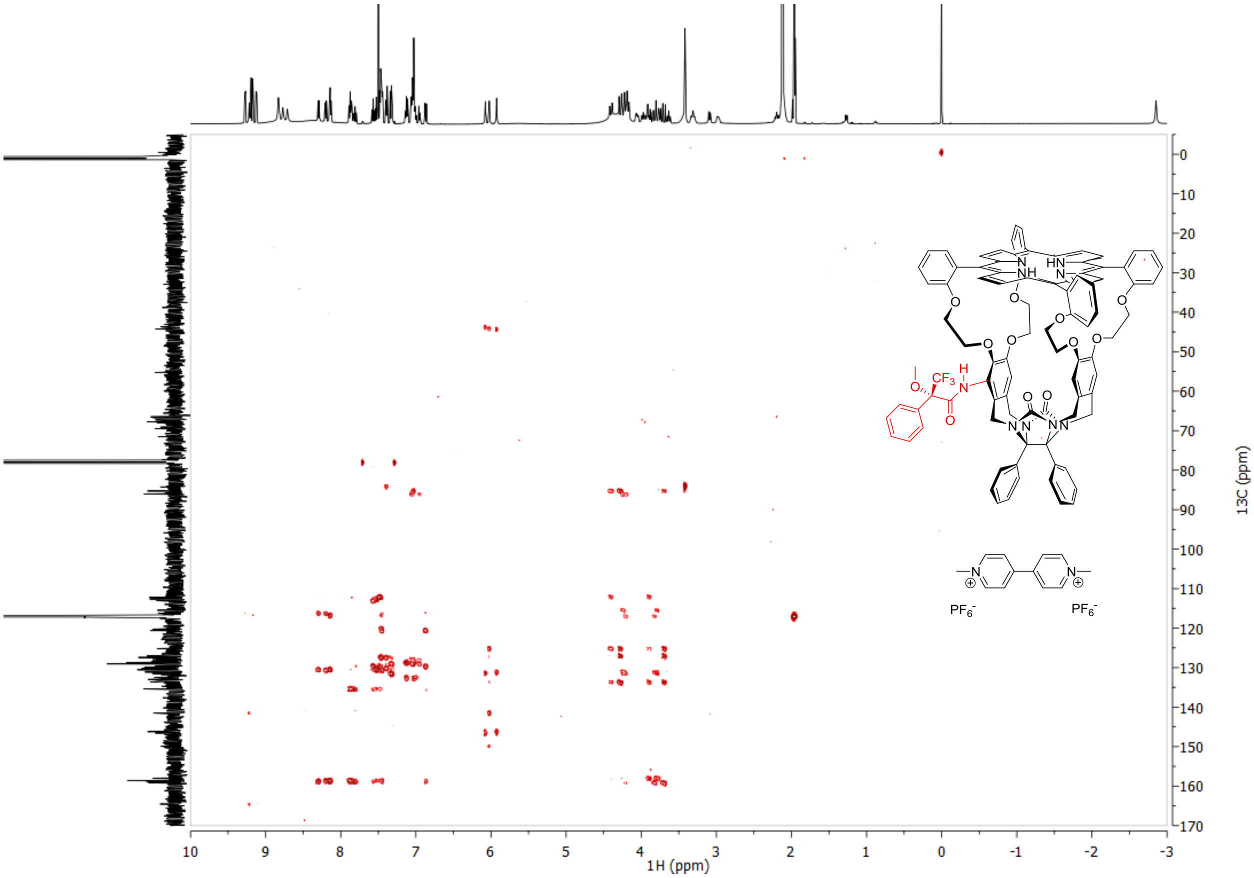

Figure S20. ^{1}H-^{13}C HMBC NMR spectrum (500 MHz, 298 K) of the (*R,S**)-$\mathbf{H_24}$ complex with **V1** in $CDCl_3$: D_3CCN, (1:1 v/v).

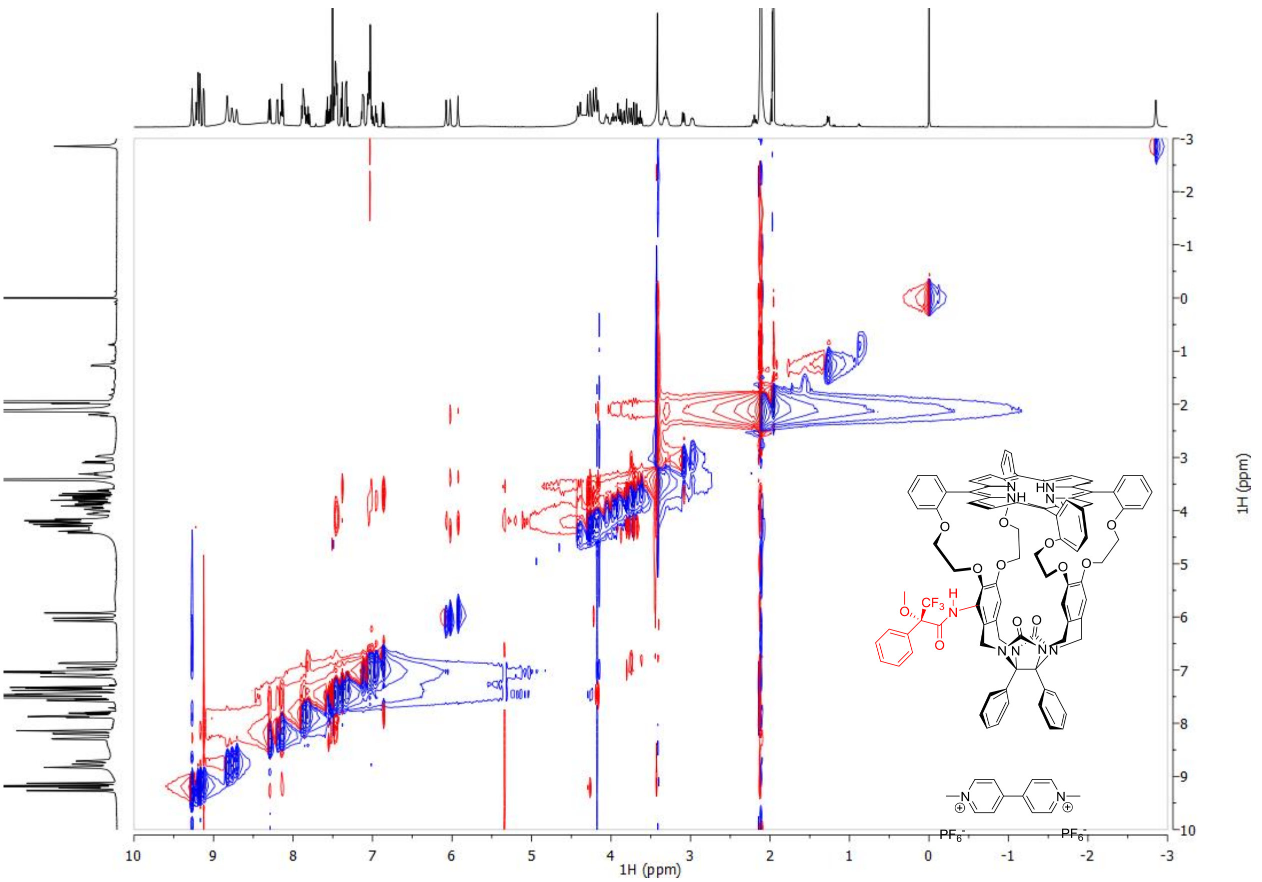

Figure S21. ^1H-^1H ROESY NMR spectrum (500 MHz, 298 K) of the (*R,S**)-**$H_2$4** complex with **V1** in $CDCl_3$: D_3CCN, (1:1 v/v).

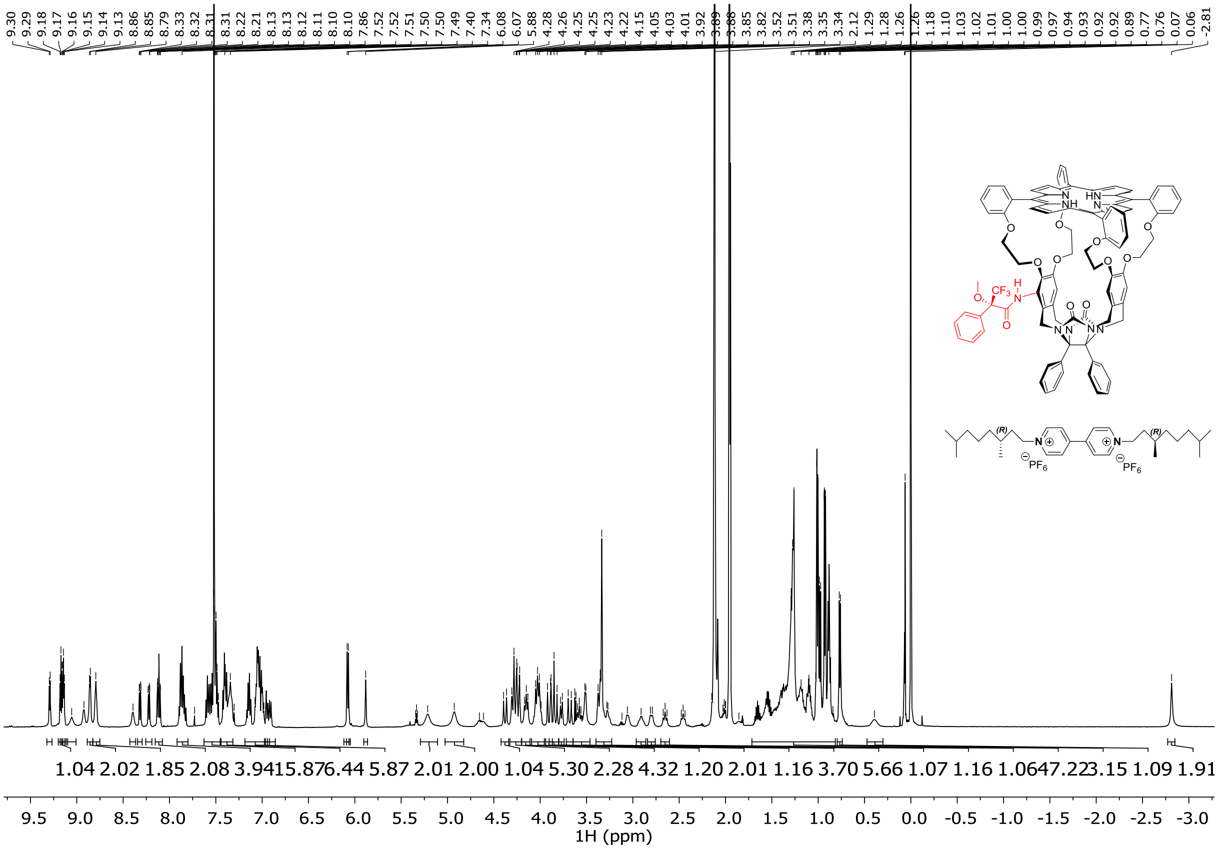

Figure S22. ^{1}H NMR spectrum (500 MHz, 298 K) of the (*R,S**)-**$H_2$4** complex with ***(R,R)*-V2** in $CDCl_3$: D_3CCN, (1:1 v/v).

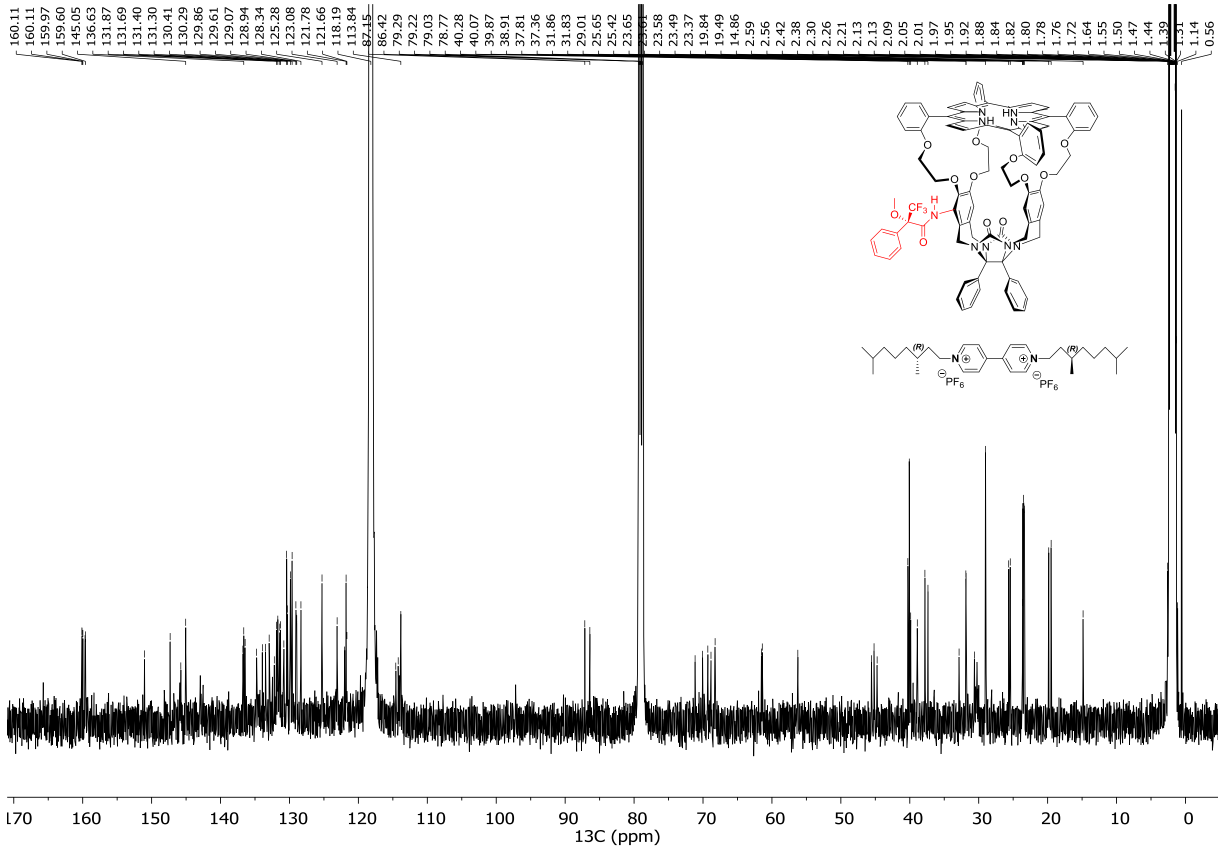

Figure S23. $^{13}C\{^1H\}$ NMR spectrum (126 MHz, 298 K) of (*R,S**)-**H$_2$4** complex with **(*R*,*R*)-V2** in $CDCl_3$: D_3CCN, (1:1 v/v).

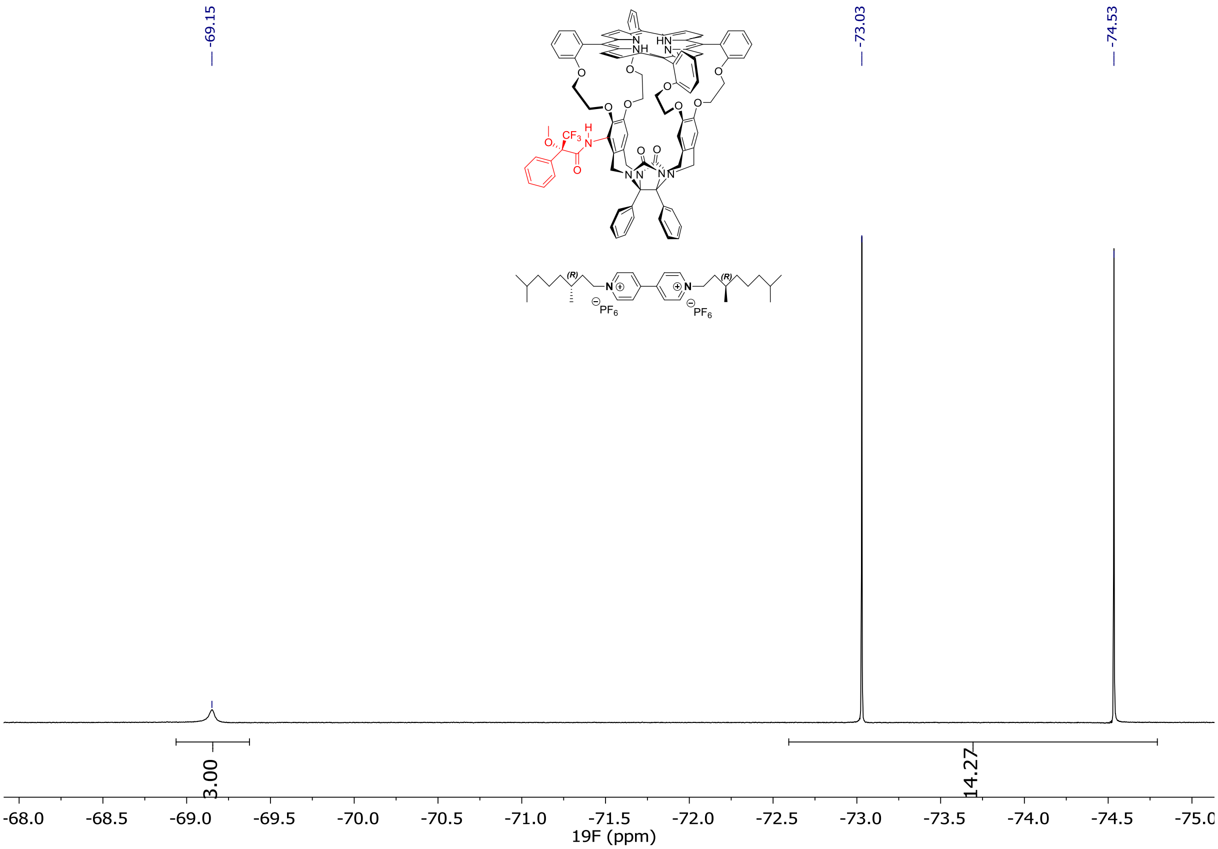

Figure S24. ^{19}F NMR spectrum (471 MHz, 298 K) of (*R,S**)-**$H_2$4** complex with **(*R*,*R*)-V2** in $CDCl_3$: D_3CCN, (1:1 v/v).

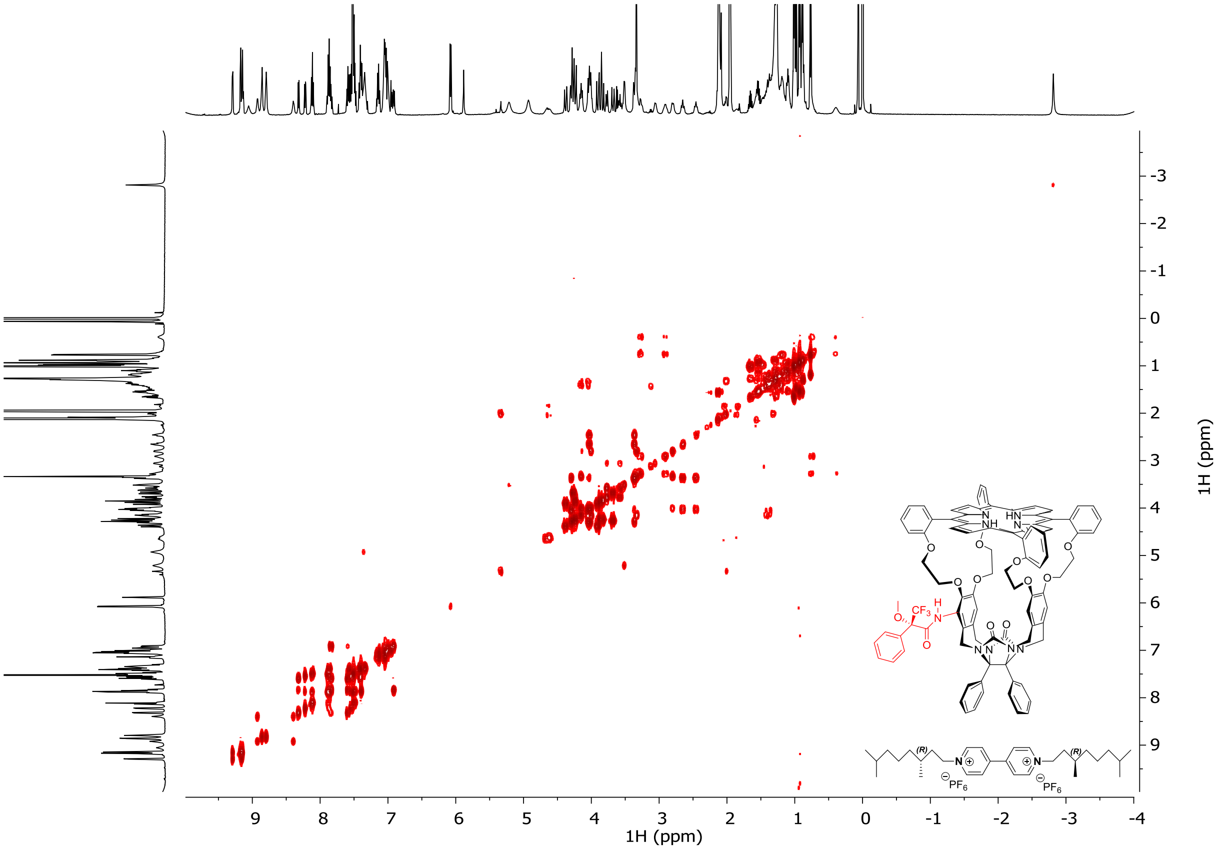

Figure S25. ^{1}H-^{1}H DQF-COSY NMR spectrum (500 MHz, 298 K) of (*R*,*S**)-$\mathbf{H_24}$ complex with **(*R*,*R*)-V2** in $CDCl_3$: D_3CCN, (1:1 v/v).

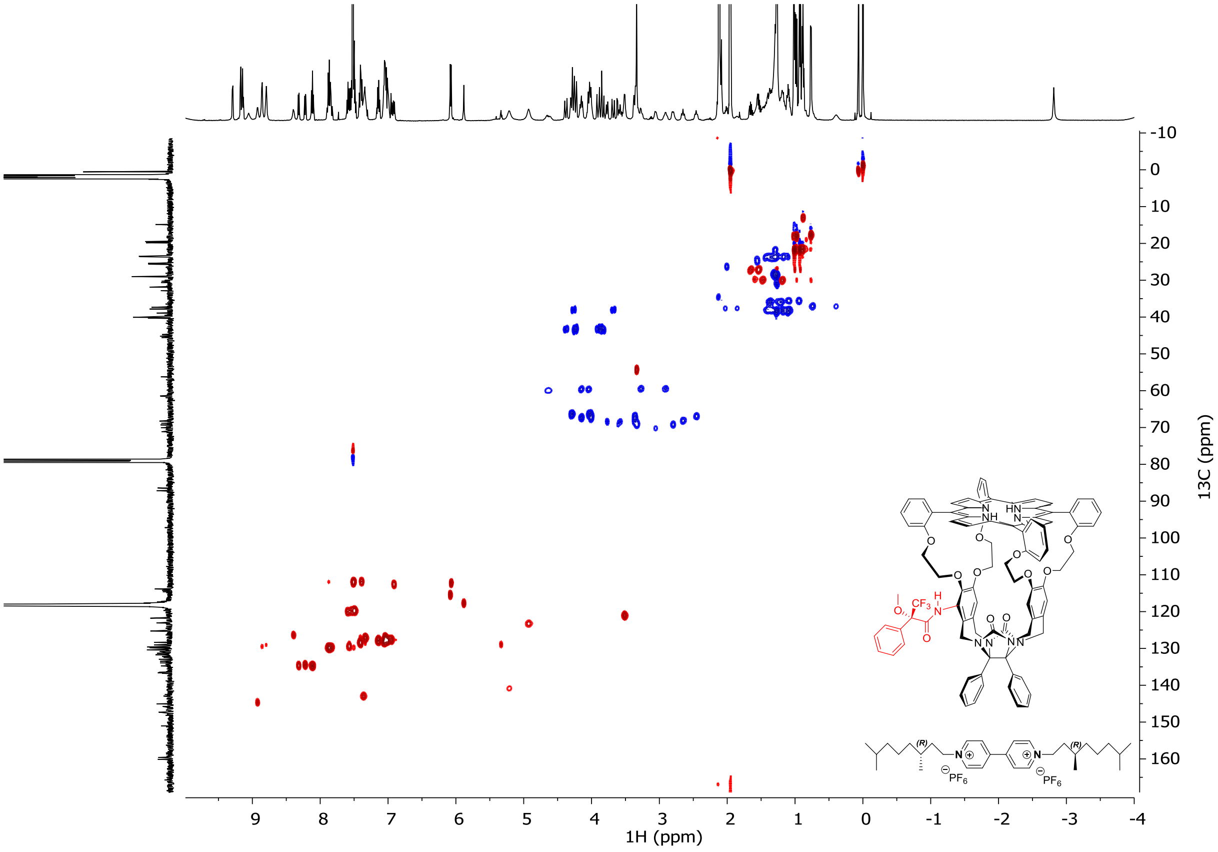

Figure S26. ^{1}H-^{13}C edited HSQC NMR spectrum (500 MHz, 298 K) of (*R*,*S**)-**$H_2$4** complex with **(*R*,*R*)-V2** in $CDCl_3$: D_3CCN, (1:1 v/v). CH_2 groups are indicated in blue and CH/CH_3 groups in red.

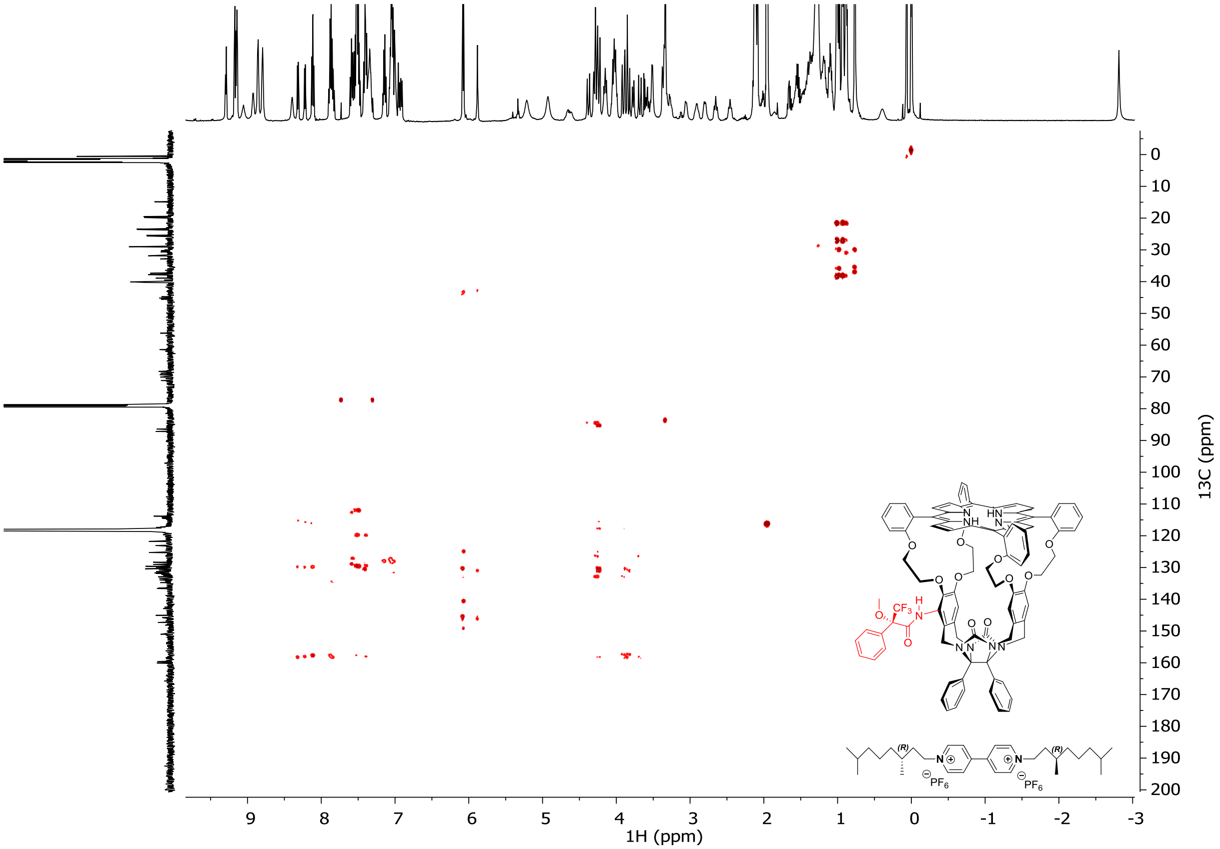

Figure S27. ^{1}H-^{13}C HMBC NMR spectrum (500 MHz, 298 K) of (*R,S**)-**$H_2$4** complex with **(*R*,*R*)-V2** in $CDCl_3$: D_3CCN, (1:1 v/v).

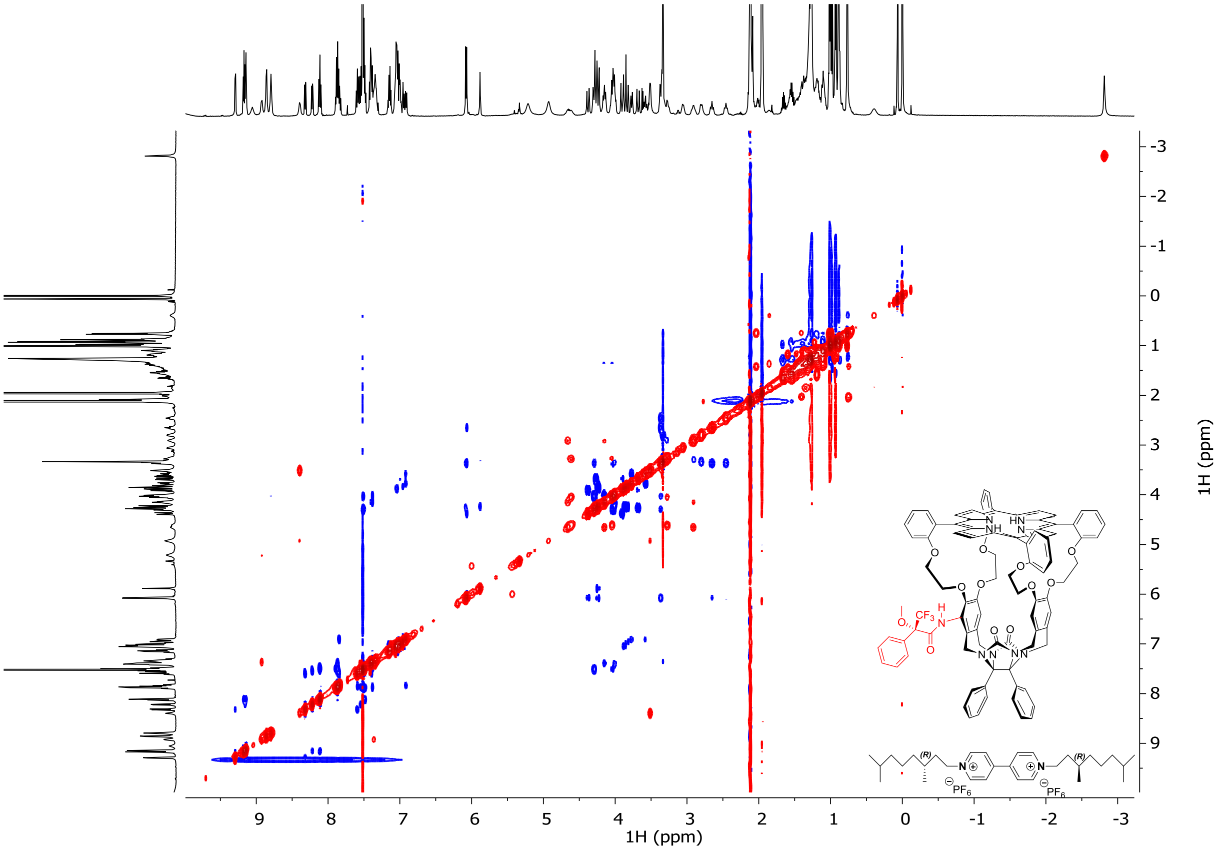

Figure S28. ^{1}H-^{1}H ROESY NMR spectrum (500 MHz, 298 K) of (*R*,*S**)-**$H_2$4** complex with **(*R*,*R*)-V2** in $CDCl_3$: D_3CCN, (1:1 v/v).

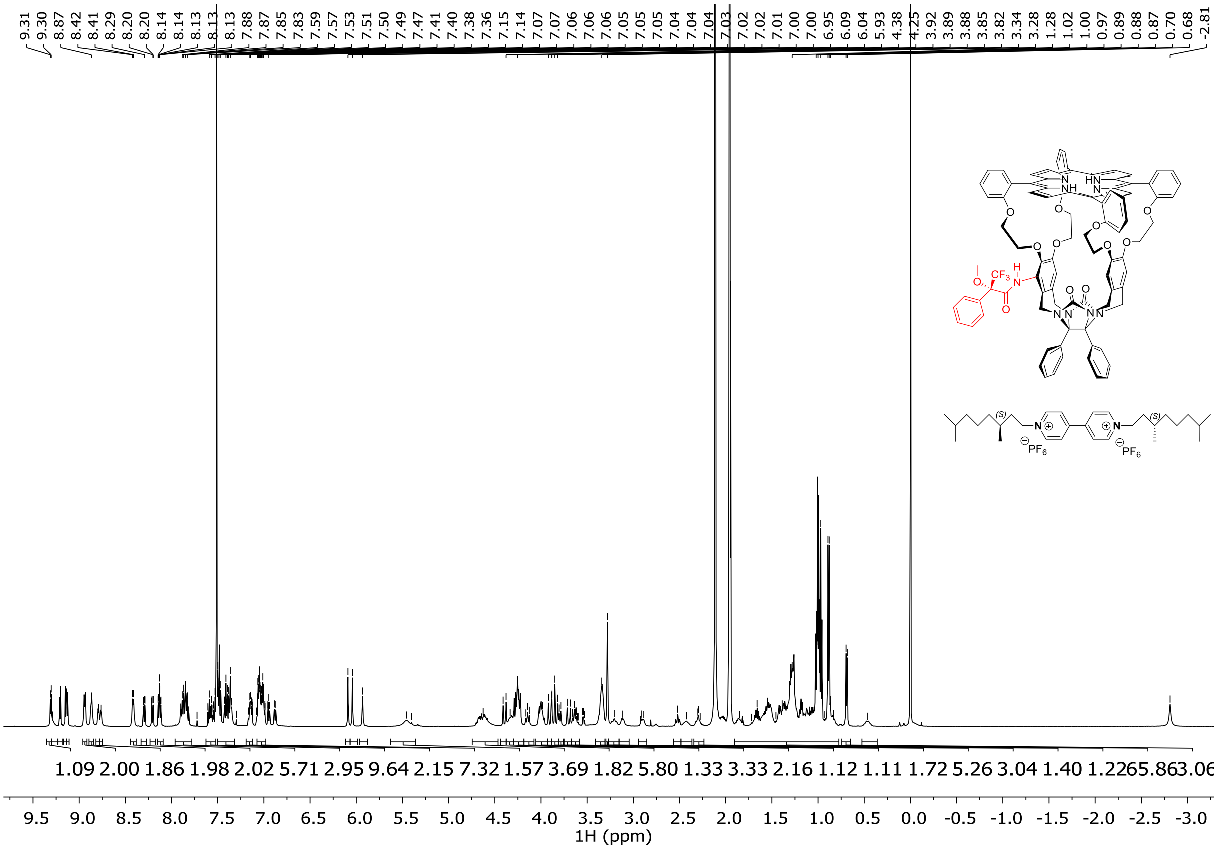

Figure S29. ^{1}H NMR spectrum (500 MHz, 298 K) of the (*R,S**)-$\mathbf{H_24}$ complex with ***(S,S)*-V2** in $CDCl_3$: D_3CCN, (1:1 v/v).

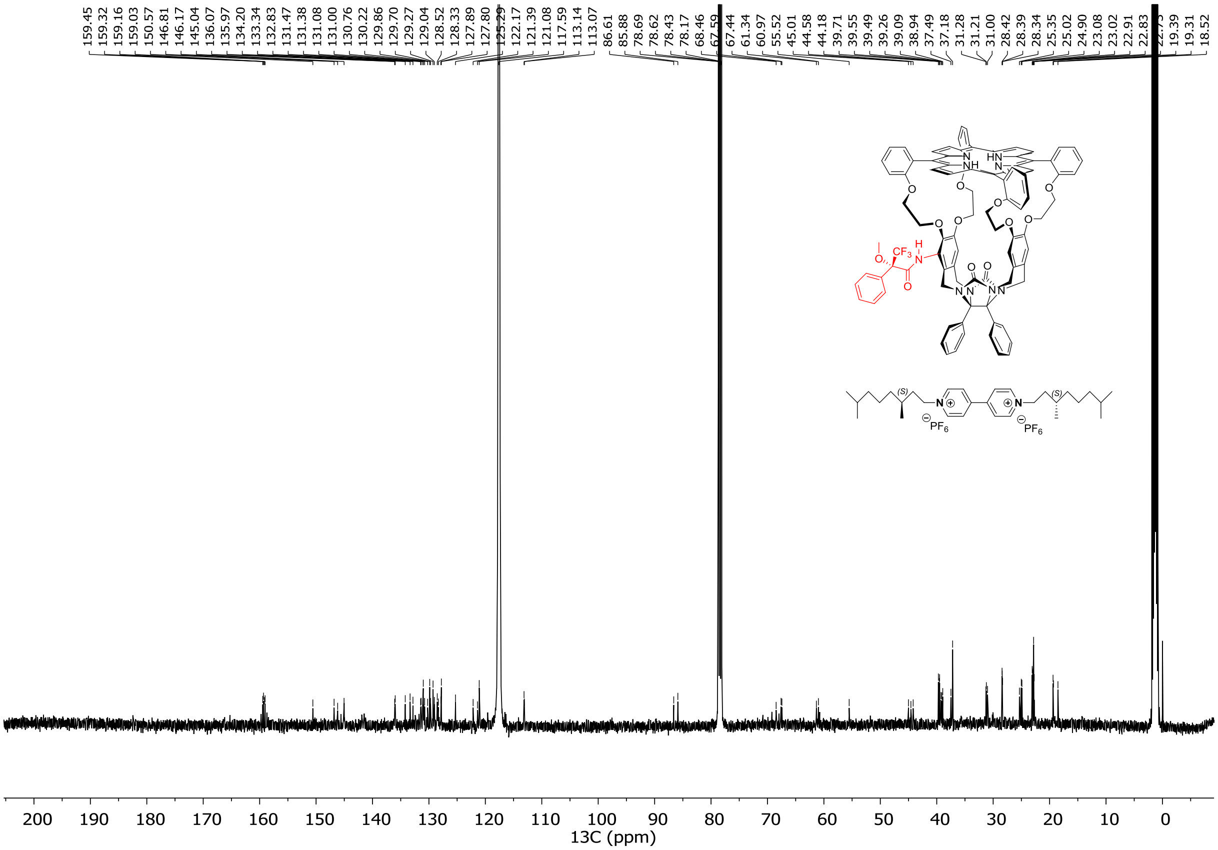

Figure S30. $^{13}C\{^1H\}$ NMR spectrum (126 MHz, 298 K) of (*R,S**)-**$H_2$4** complex with ***(S,S)*-V2** in $CDCl_3$: D_3CCN, (1:1 v/v).

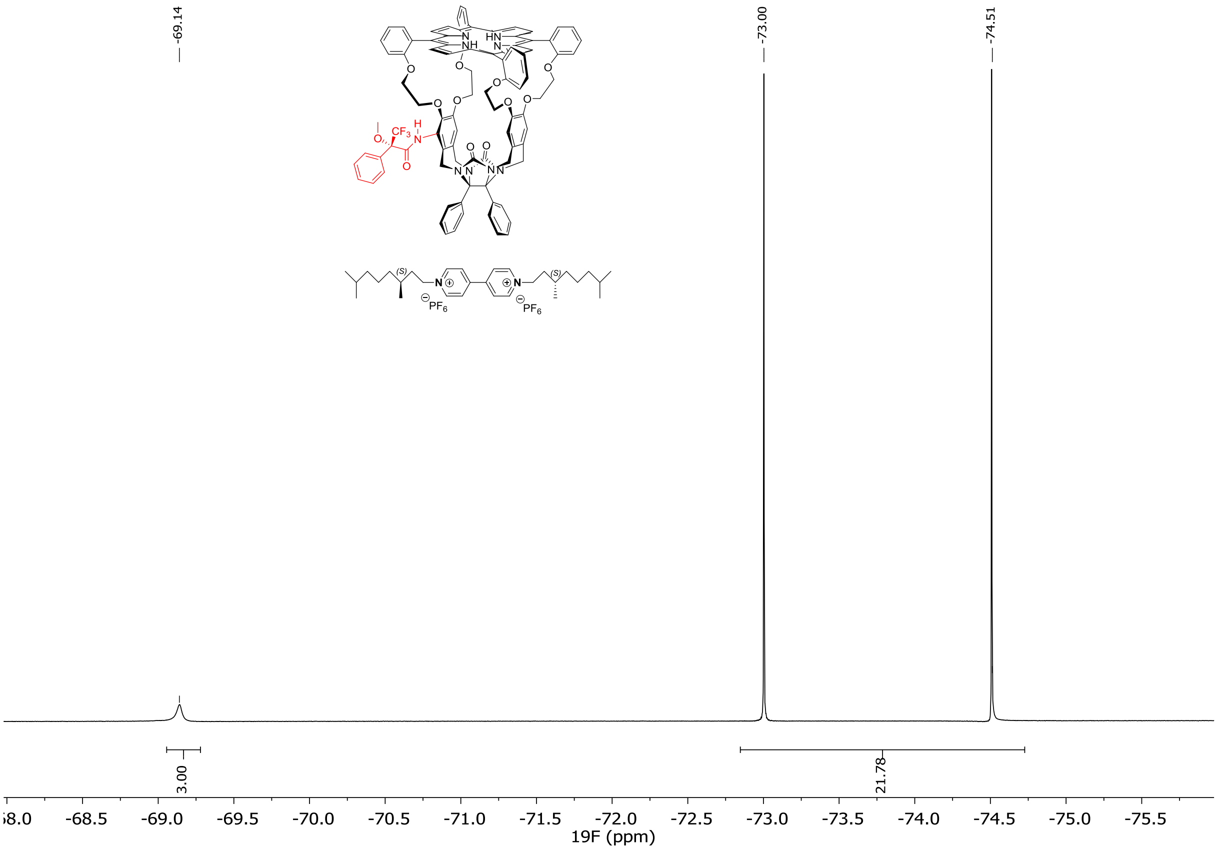

Figure S31. ^{19}F NMR spectrum (471 MHz, 298 K) of (*R,S**)-**$H_2$4** complex with **(*R*,*R*)-V2** in $CDCl_3$: D_3CCN, (1:1 v/v).

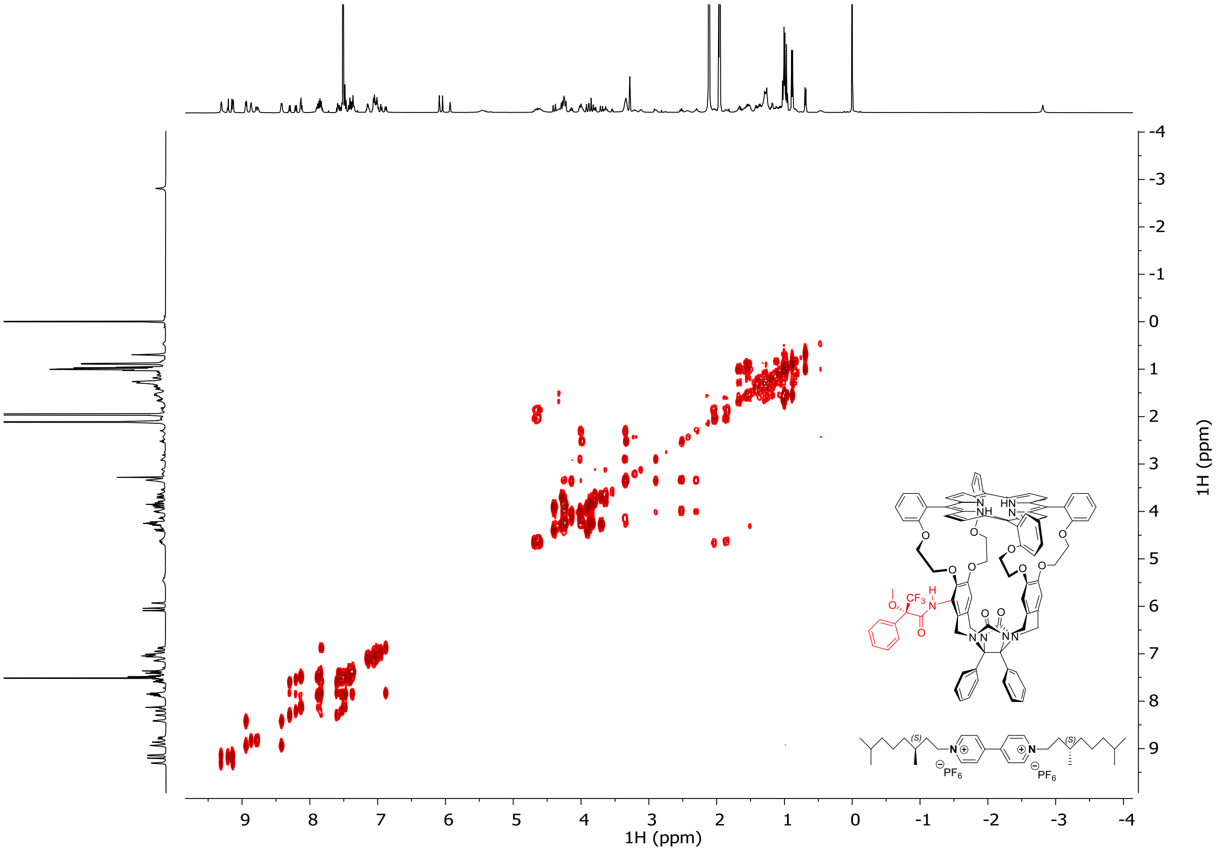

Figure S32. ^{1}H-^{1}H DQF-COSY NMR spectrum (500 MHz, 298 K) of (*R*,*S**)-**H_24** complex with **(*S*,*S*)-V2** in $CDCl_3$: D_3CCN, (1:1 v/v).

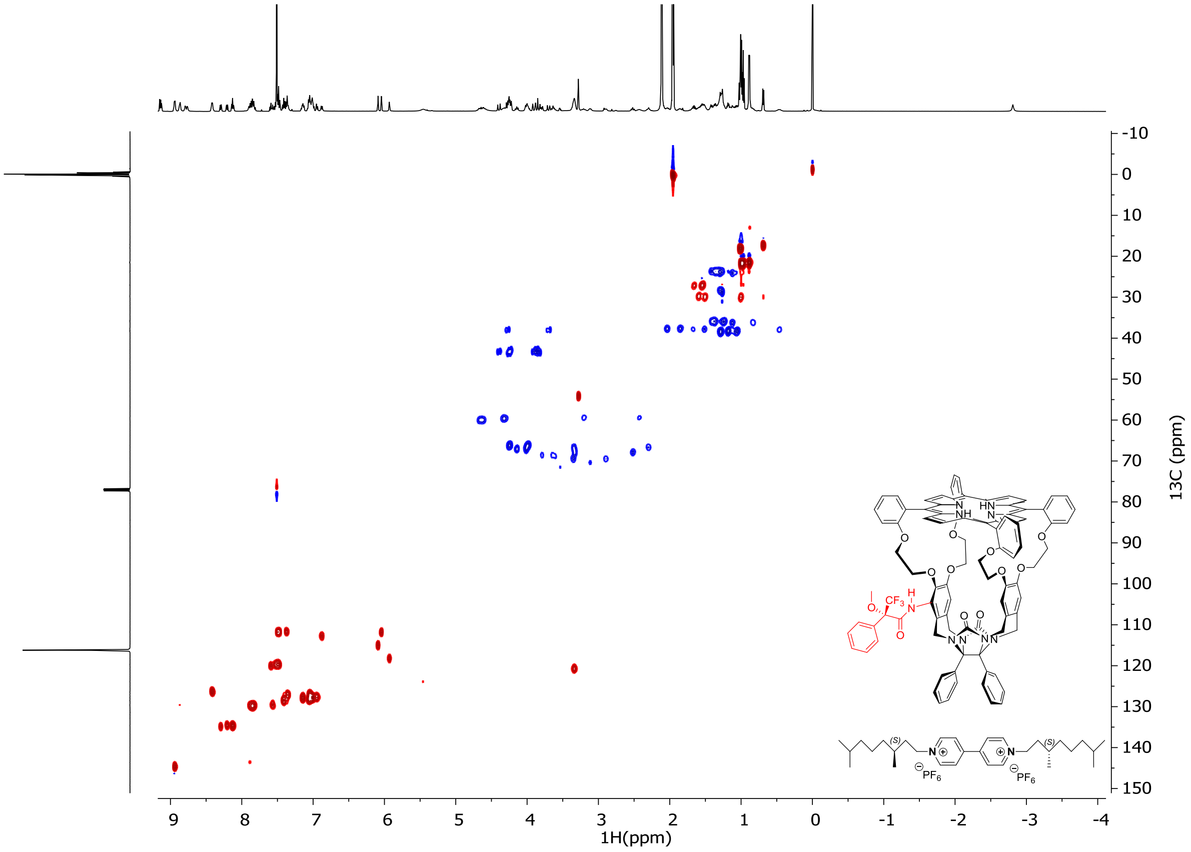

Figure S33. ^1H-^{13}C edited HSQC NMR spectrum (500 MHz, 298 K) of (*R*,*S**)-**$H_2$4** complex with **(*S*,*S*)-V2** in $CDCl_3$: D_3CCN, (1:1 v/v). CH_2 groups are indicated in blue and CH/CH_3 groups in red.

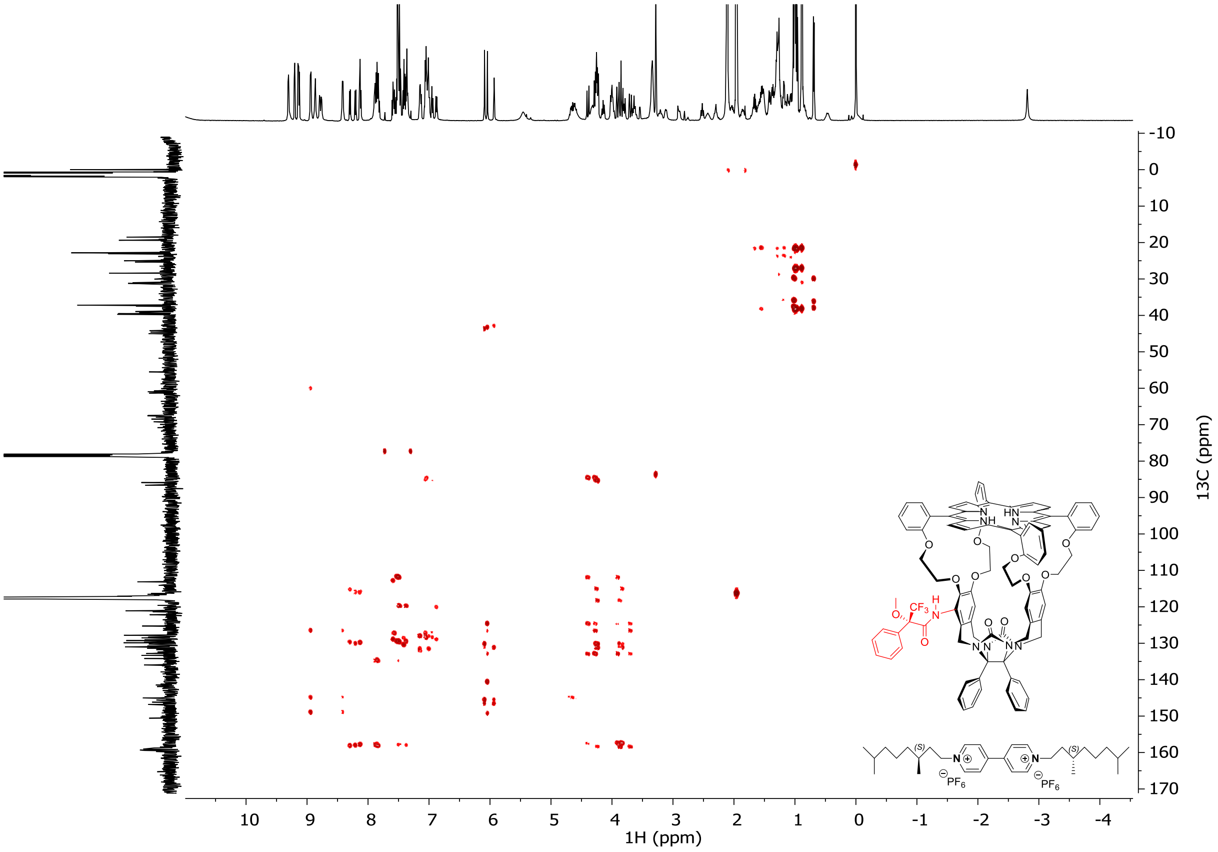

Figure S34. ^{1}H-^{13}C HMBC NMR spectrum (500 MHz, 298 K) of (*R,S**)-**$H_2$4** complex with **(*S*,*S*)-V2** in $CDCl_3$: D_3CCN, (1:1 v/v).

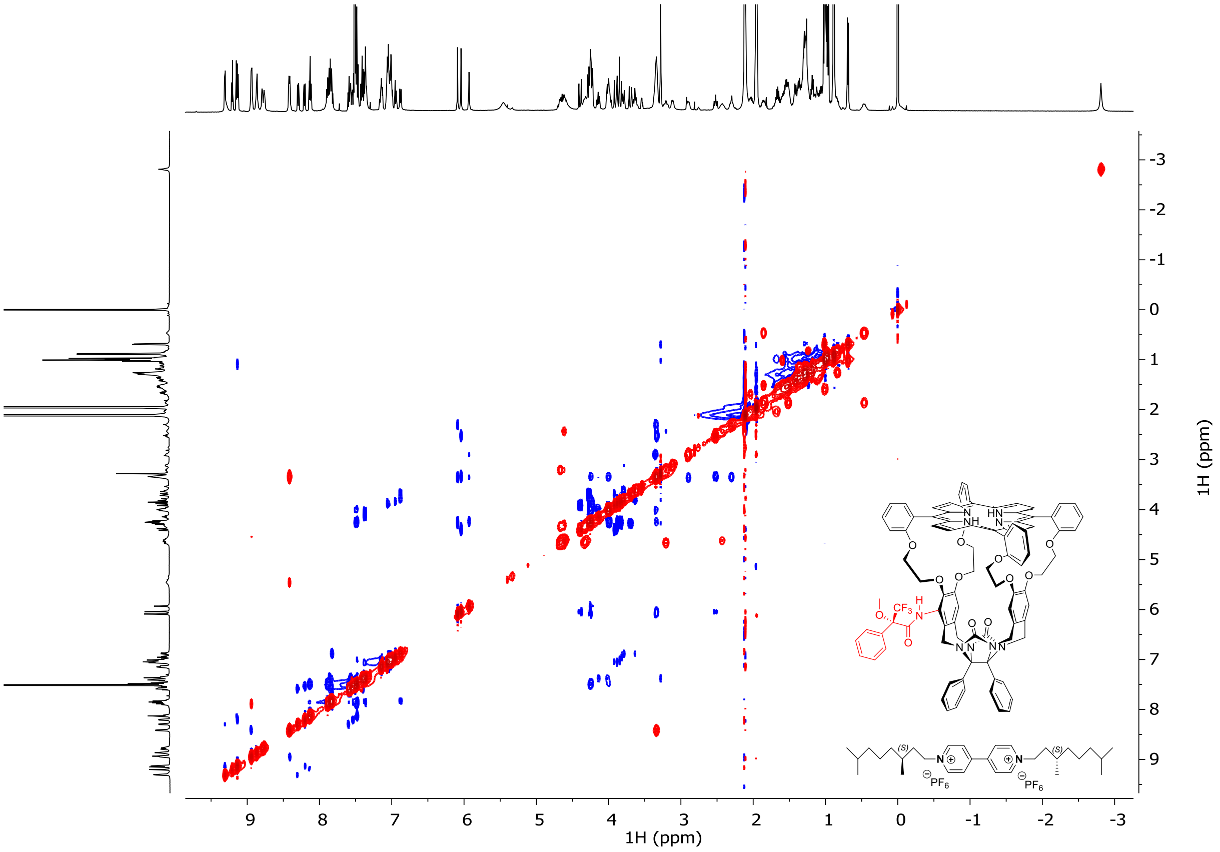

Figure S35. ^{1}H-^{1}H ROESY NMR spectrum (500 MHz, 298 K) of (*R,S**)-**$H_2$4** complex with ***(S,S)*-V2** in $CDCl_3$: D_3CCN, (1:1 v/v).

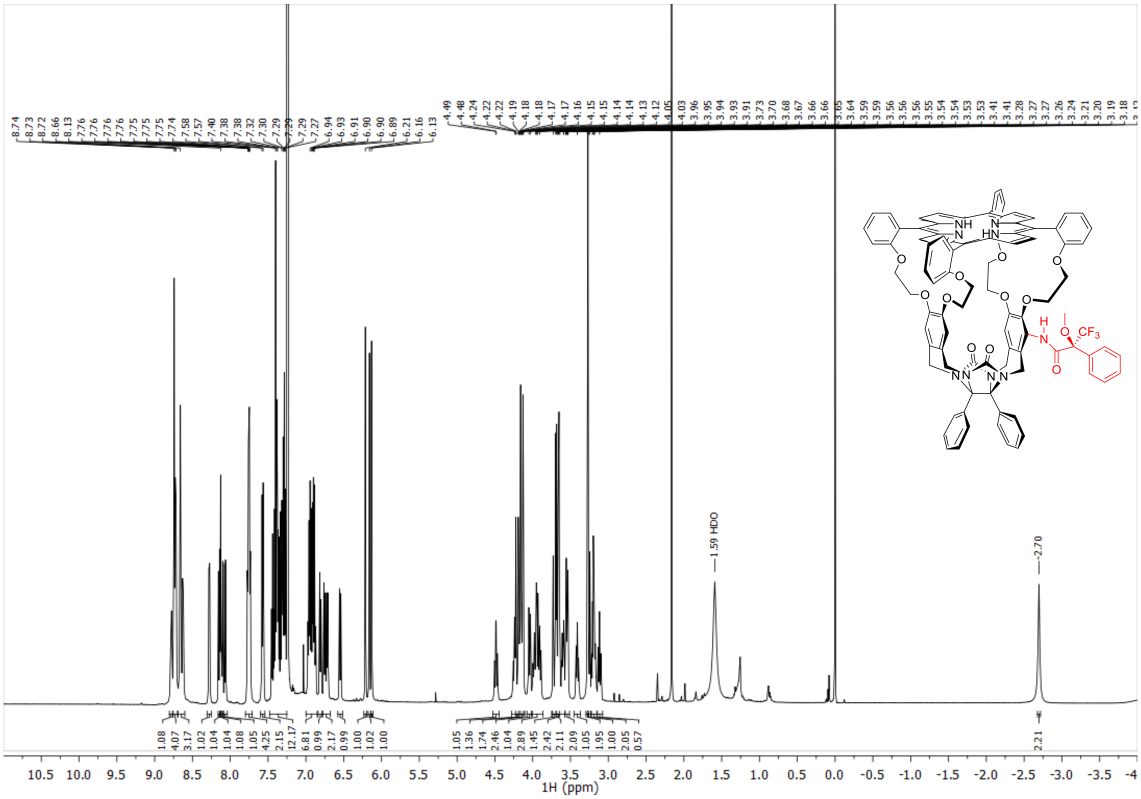

Figure S36. ^{1}H NMR spectrum (500 MHz, 298 K) of (*R*,*R*$_p$*)-**H$_2$5** in $CDCl_3$.

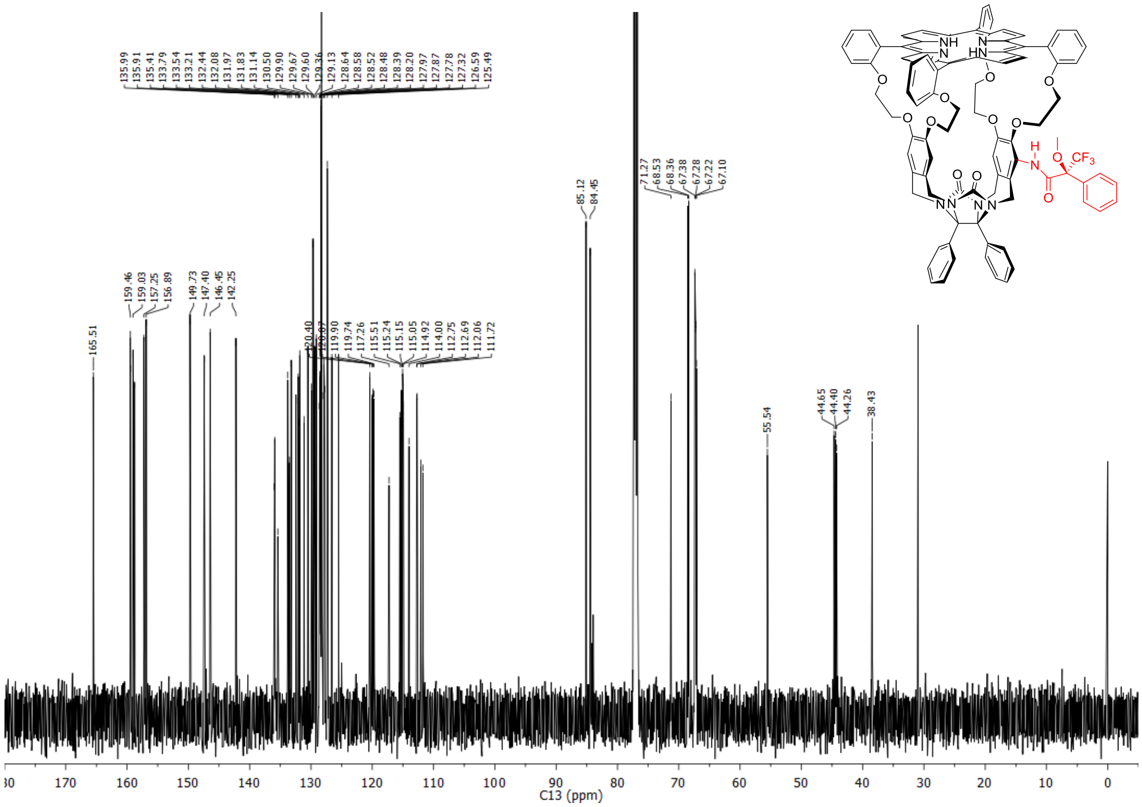

Figure S37. $^{13}C\{^{1}H\}$ NMR spectrum (126 MHz, 298 K) of (*R*,*R*$_p$*)-**H$_2$5** in $CDCl_3$.

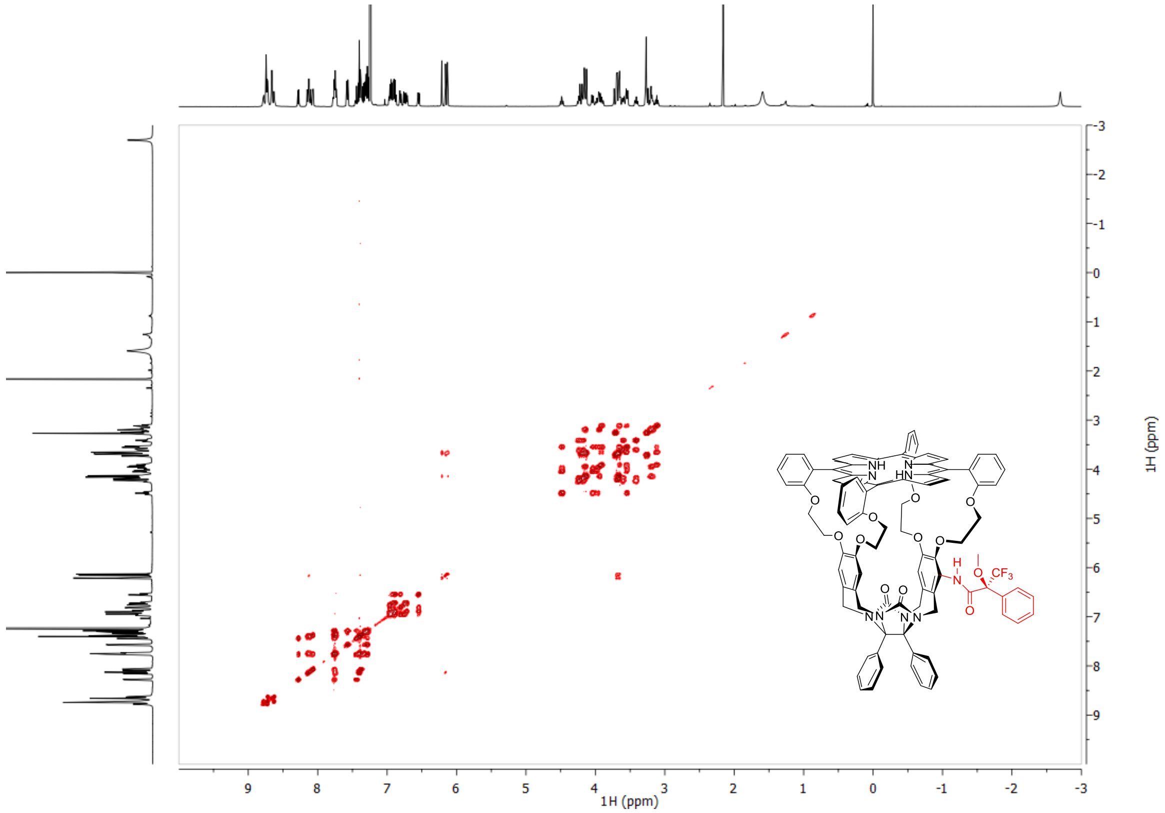

Figure S38. ^{1}H-^{1}H DQF-COSY NMR spectrum (500 MHz, 298 K) of (*R*,R_p*)-**$H_2$5** in $CDCl_3$.

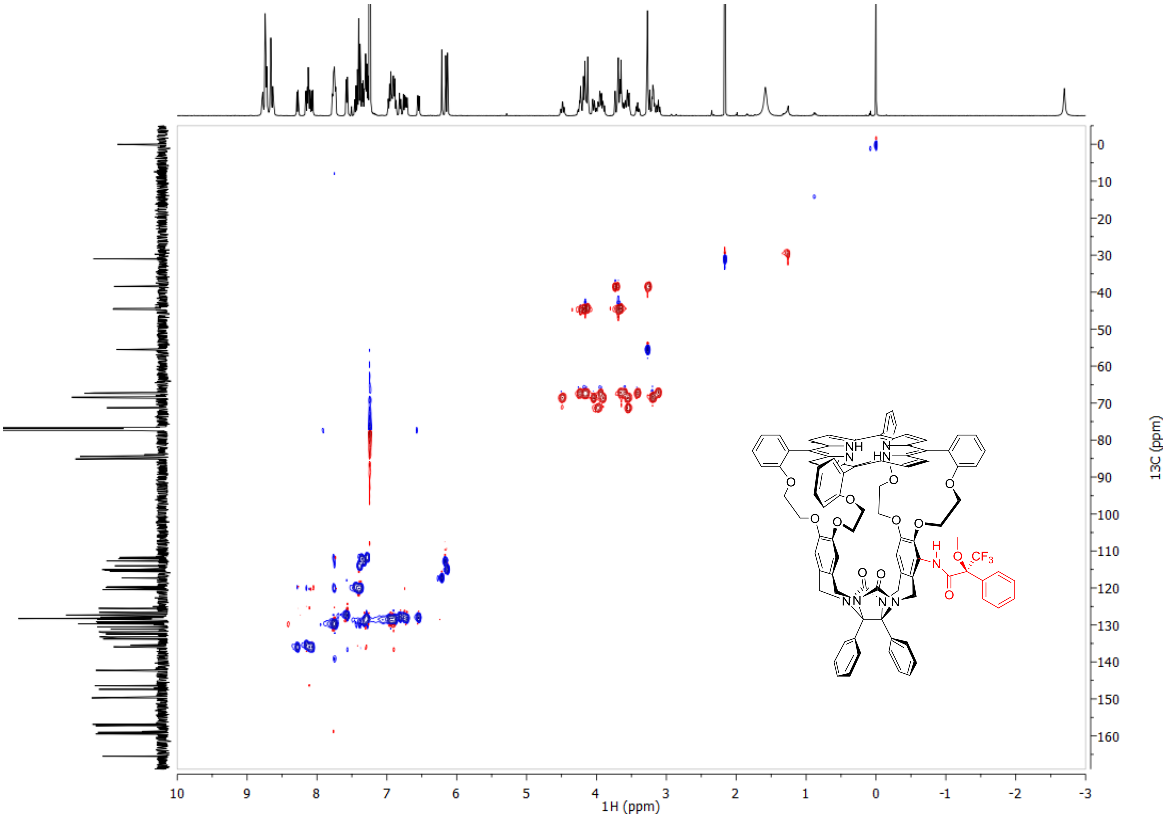

Figure S39. ^{1}H-^{13}C edited HSQC NMR spectrum (500 MHz, 298 K) of (*R*,*R*$_p$*)-**H$_2$5** in $CDCl_3$. CH_2 groups are indicated in blue and CH/CH_3 groups in red.

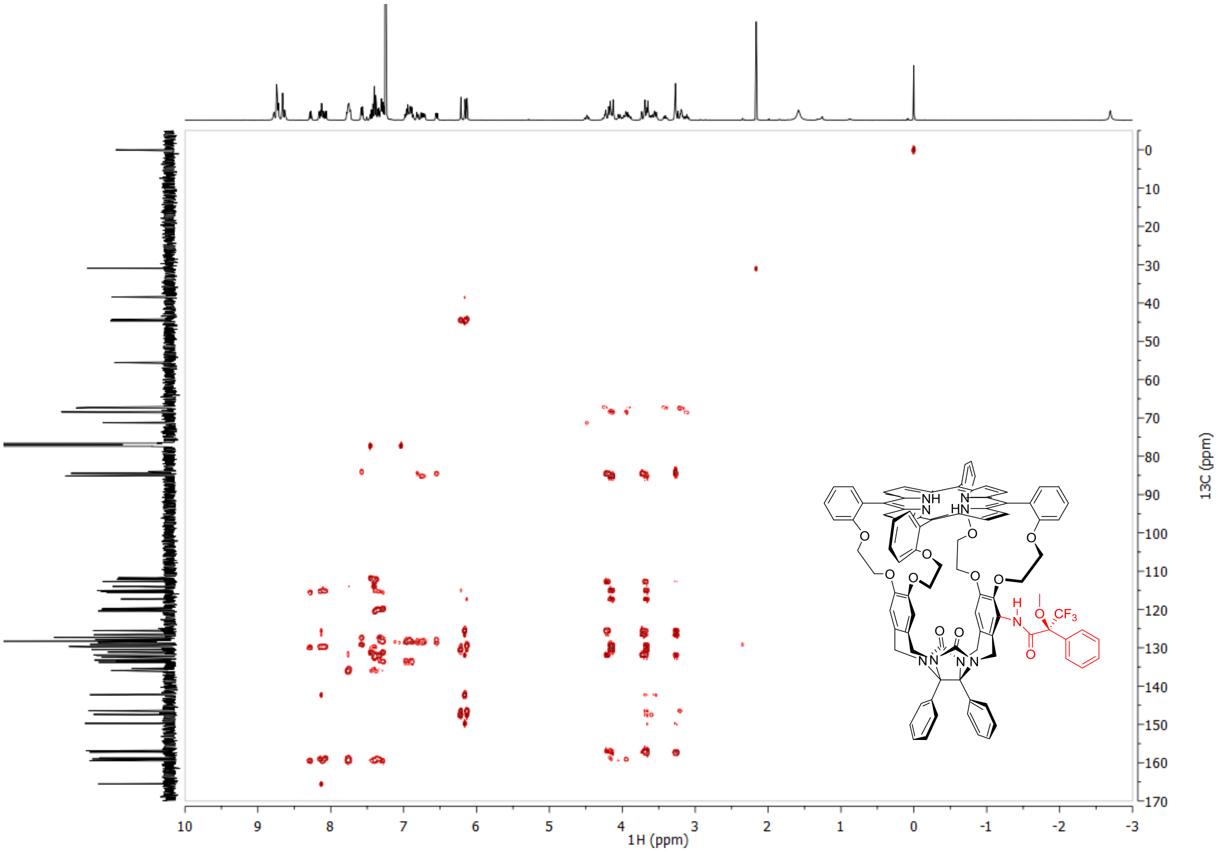

Figure S40. ^{1}H-^{13}C HMBC NMR spectrum (500 MHz, 298 K) of (*R*,*R*$_p$*)-**H$_2$5** in $CDCl_3$.

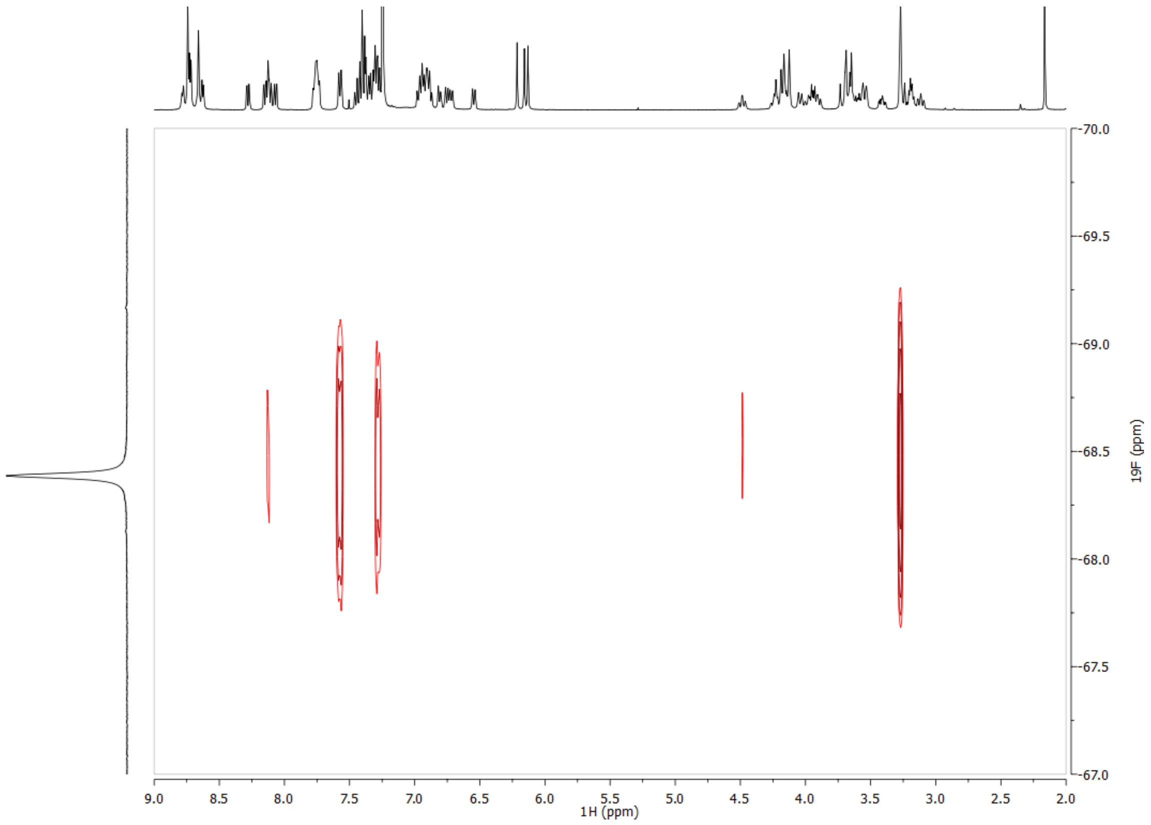

Figure S41. ^{1}H-^{19}F HOESY NMR spectrum (500 MHz, 298 K) of (*R*,*R*$_p$*)-**H$_2$5** in $CDCl_3$.

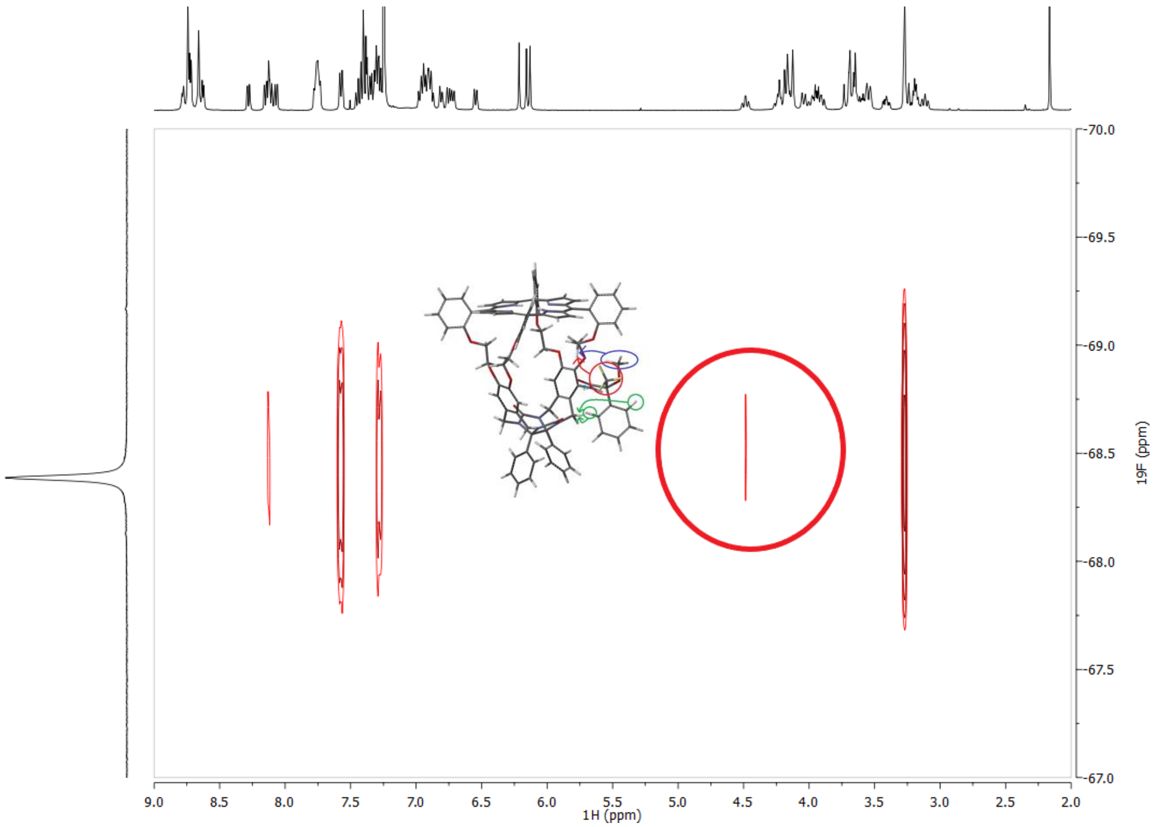

Figure S42. ^{1}H-^{19}F HOESY NMR spectrum (500 MHz, 298 K) of (*R,R$_p$**)-**H$_2$5** in $CDCl_3$.(Key nOe contacts encircled).

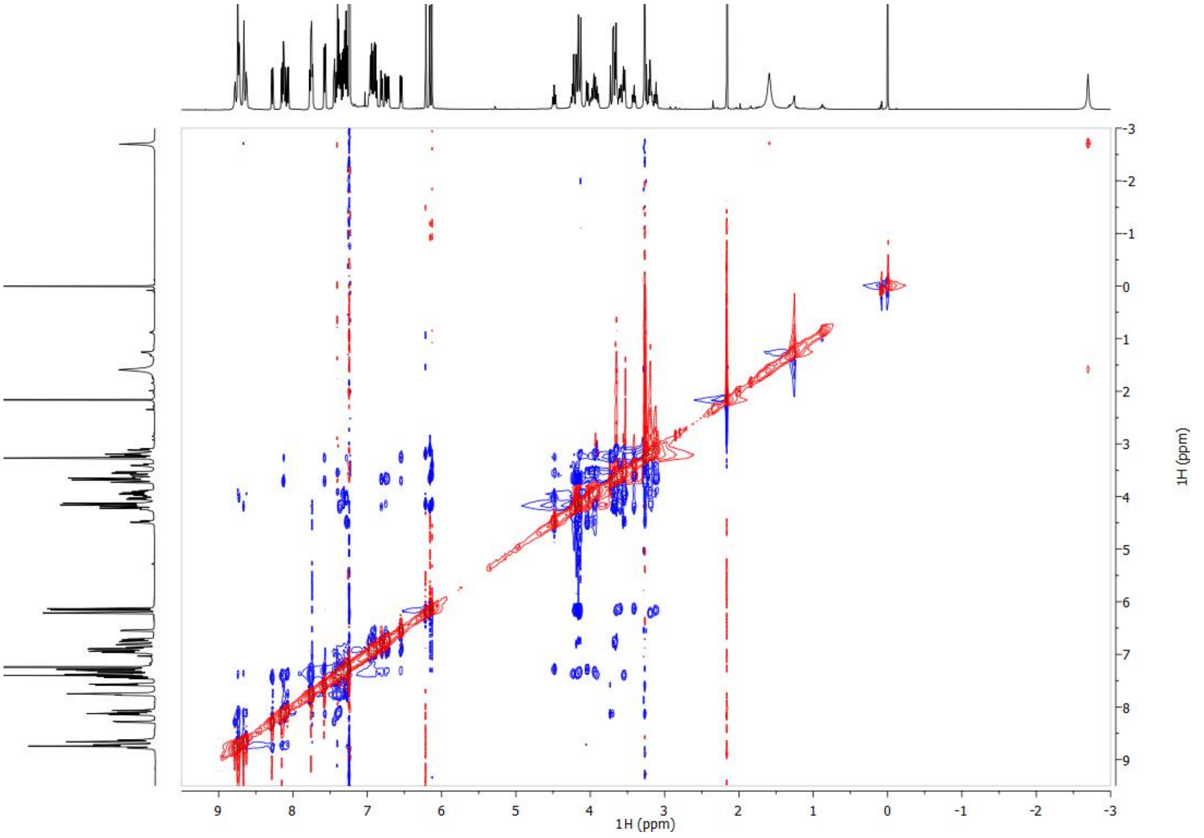

Figure S43. ^{1}H-^{1}H ROESY NMR spectrum (500 MHz, 298 K) of (*R,R*$_p$*)-$\mathbf{H_25}$ in $CDCl_3$.

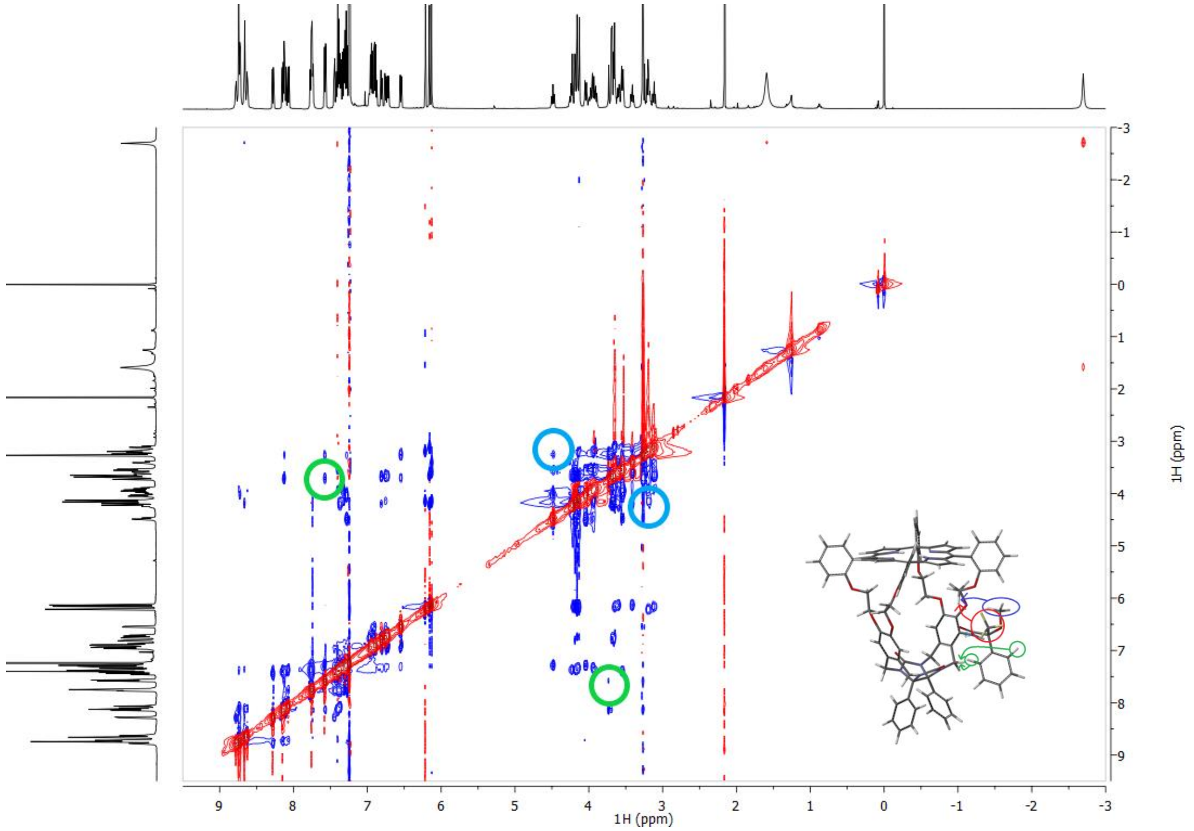

Figure S44. ^{1}H-^{1}H ROESY NMR spectrum (500 MHz, 298 K) of (*R,R$_p$**)-**$H_2$5** in $CDCl_3$.(Key nOe contacts encircled).

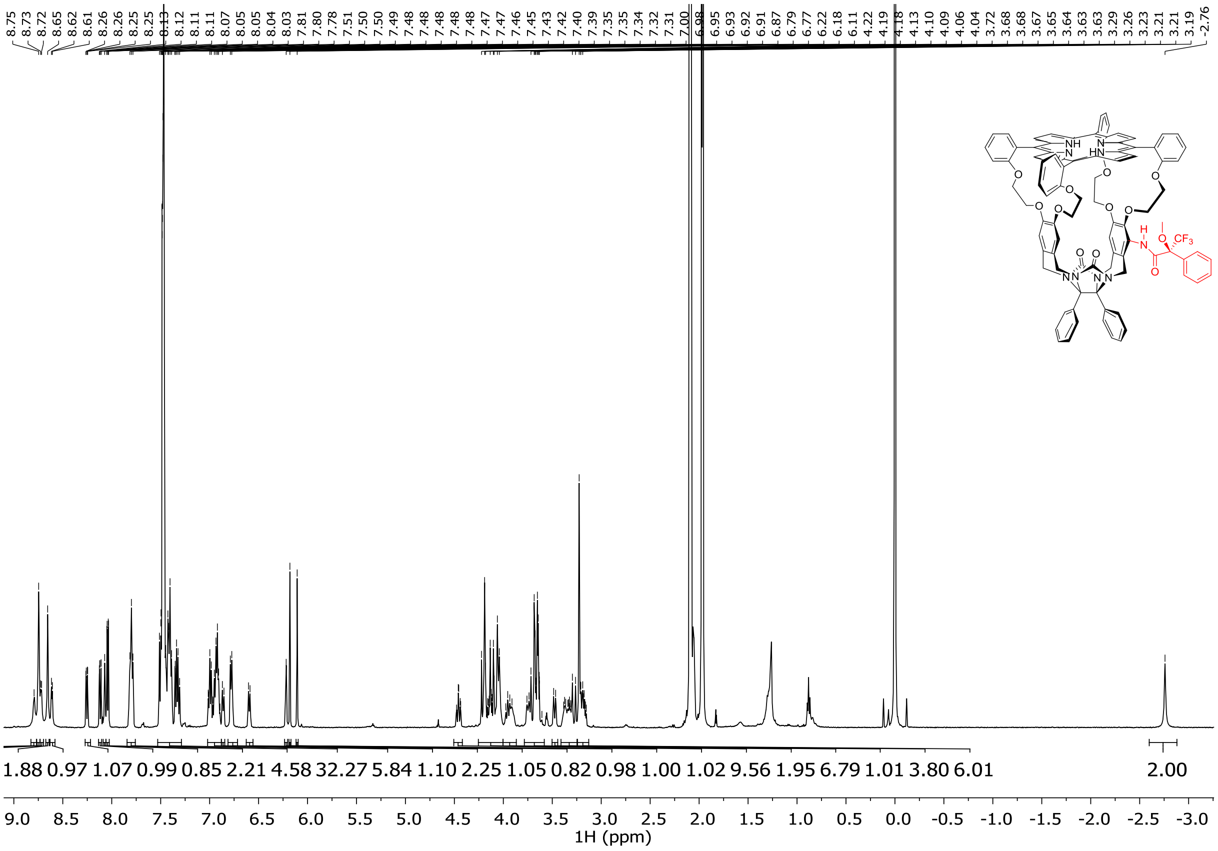

Figure S45. ^{1}H NMR spectrum (500 MHz, 298 K) of (*R,R**)-$\mathbf{H_25}$ in $CDCl_3$: D_3CCN, (1:1 v/v).

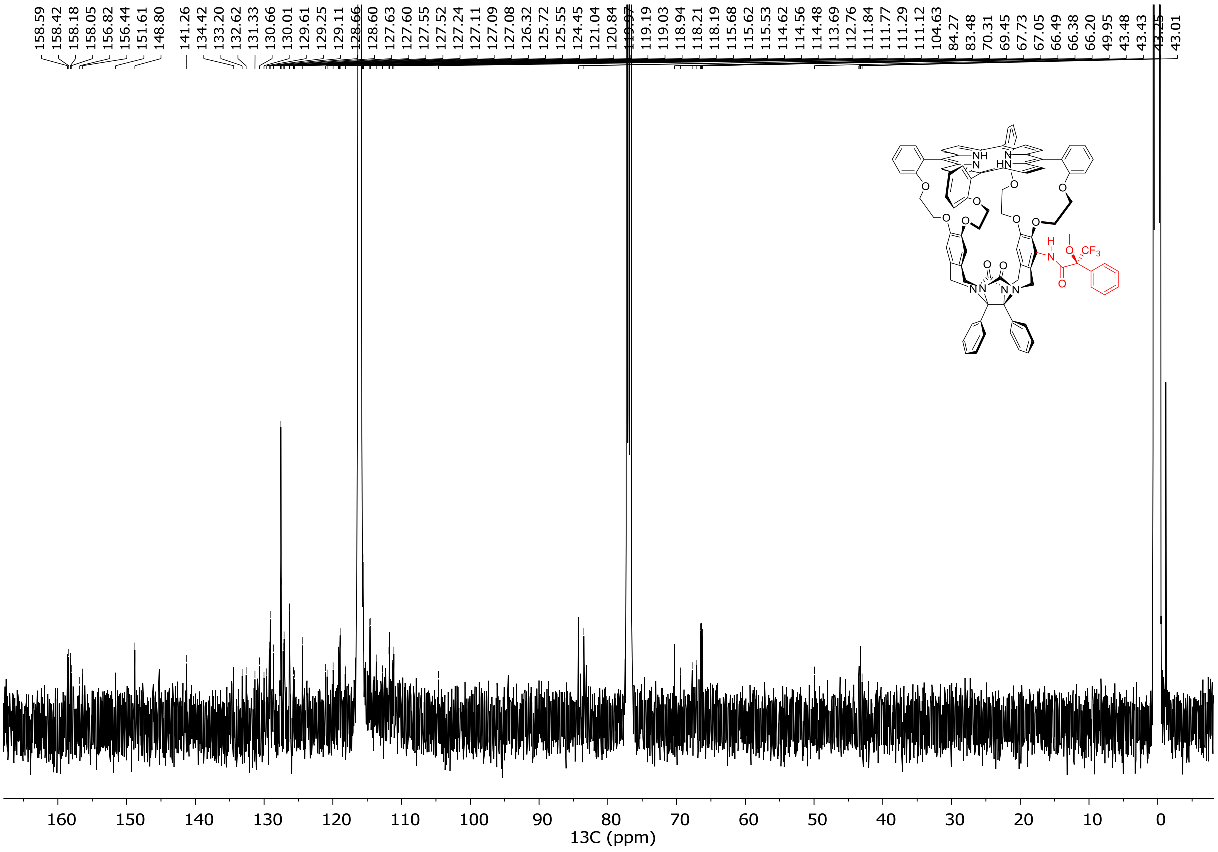

Figure S46. $^{13}C\{^{1}H\}$ NMR spectrum (126 MHz, 298 K) of (*R*,*R**)-$\mathbf{H_25}$ in $CDCl_3$: D_3CCN, (1:1 v/v).

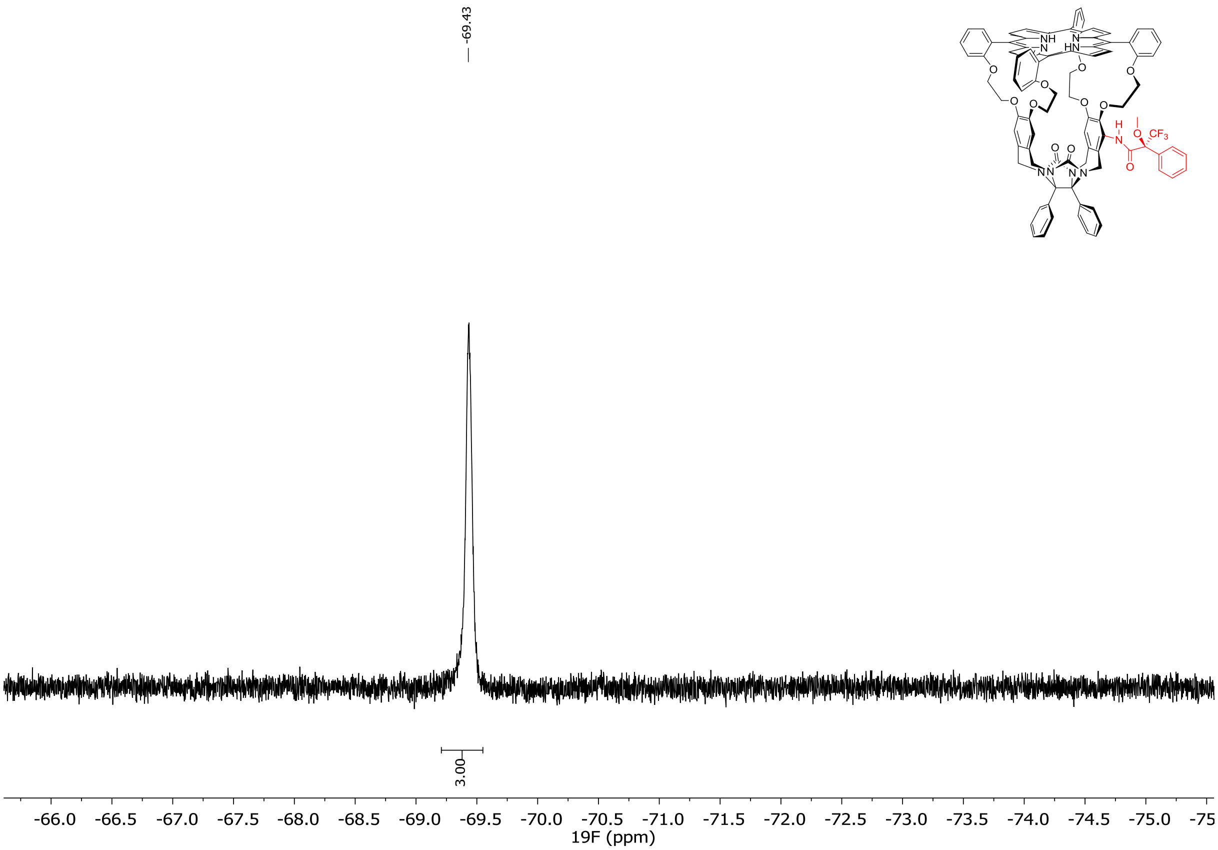

Figure S47. ^{19}F NMR spectrum (471 MHz, 298 K) of (*R,R**)-$\mathbf{H_25}$ $CDCl_3$: D_3CCN, (1:1 v/v).

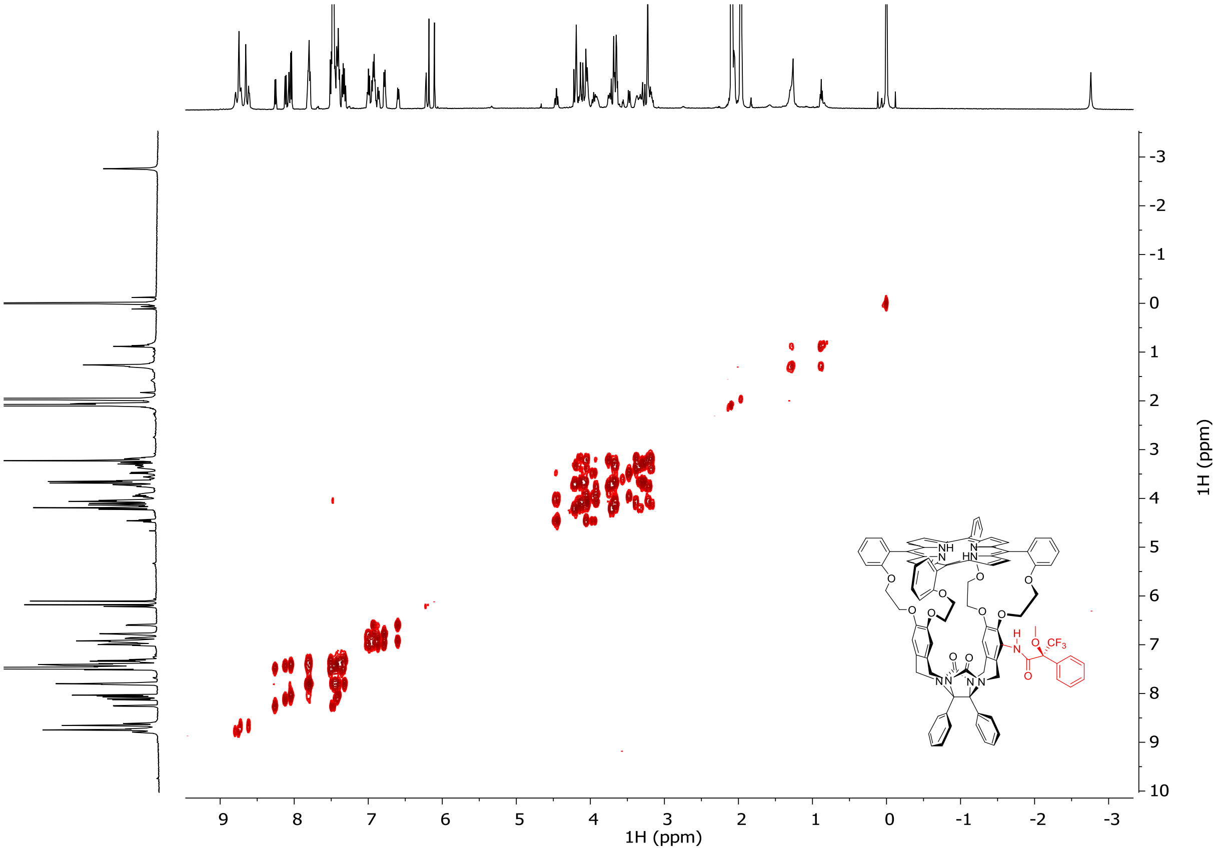

Figure S48. ^{1}H-^{1}H DQF-COSY NMR spectrum (500 MHz, 298 K) of (*R,R**)-**$H_2$5** in $CDCl_3$: D_3CCN, (1:1 v/v).

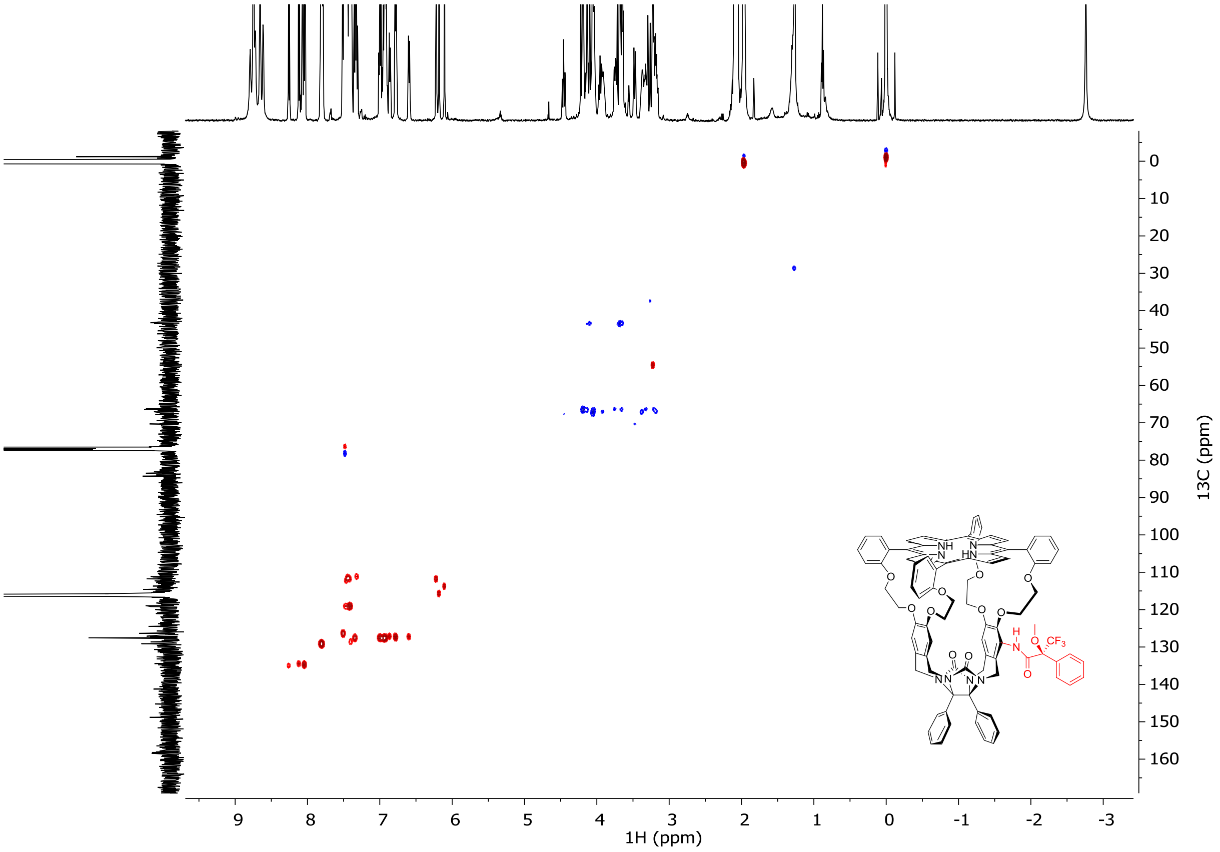

Figure S49. ^{1}H-^{13}C edited HSQC NMR spectrum (500 MHz, 298 K) of (*R,R**)-**$H_2$5** in $CDCl_3$: D_3CCN, (1:1 v/v). CH_2 groups are indicated in blue and CH/CH_3 groups in red.

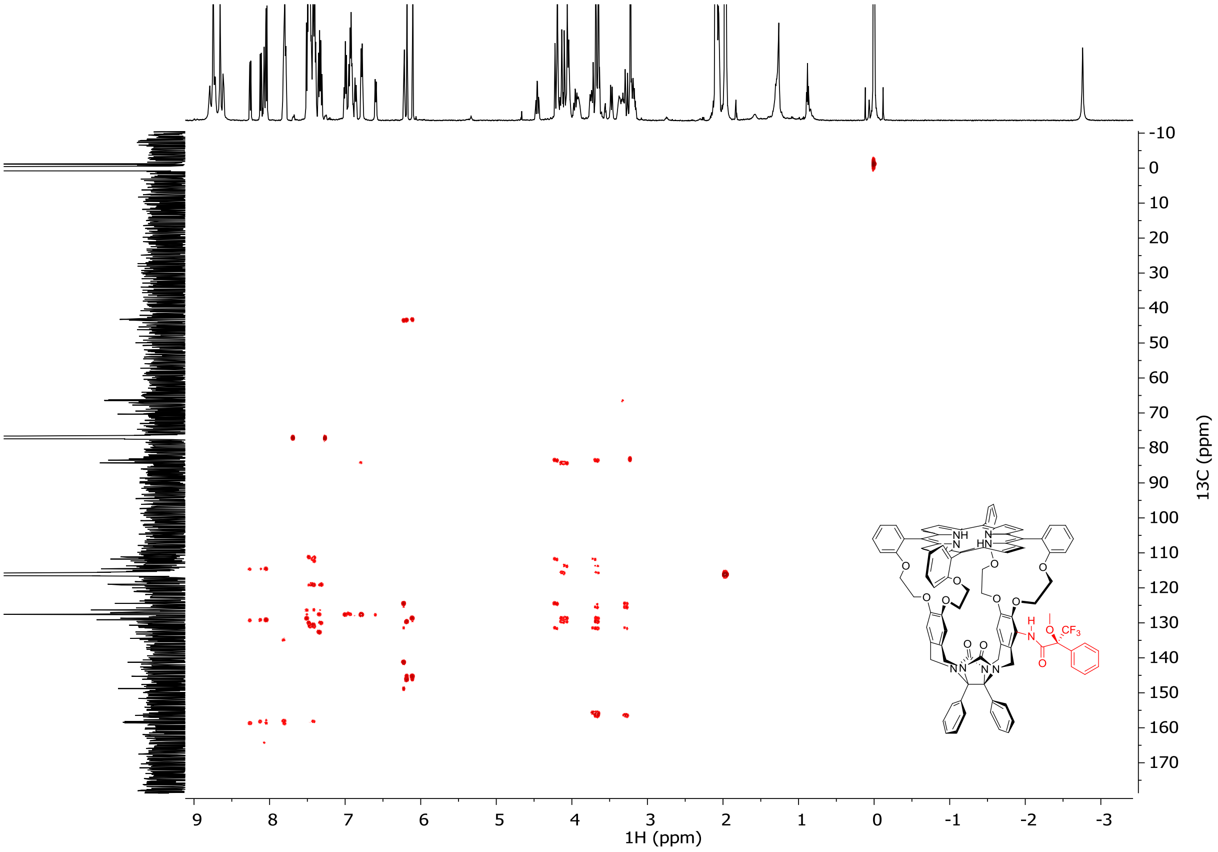

Figure S50. ^{1}H-^{13}C HMBC NMR spectrum (500 MHz, 298 K) of (*R*,*R**)-**$H_2$5** in $CDCl_3$: D_3CCN, (1:1 v/v).

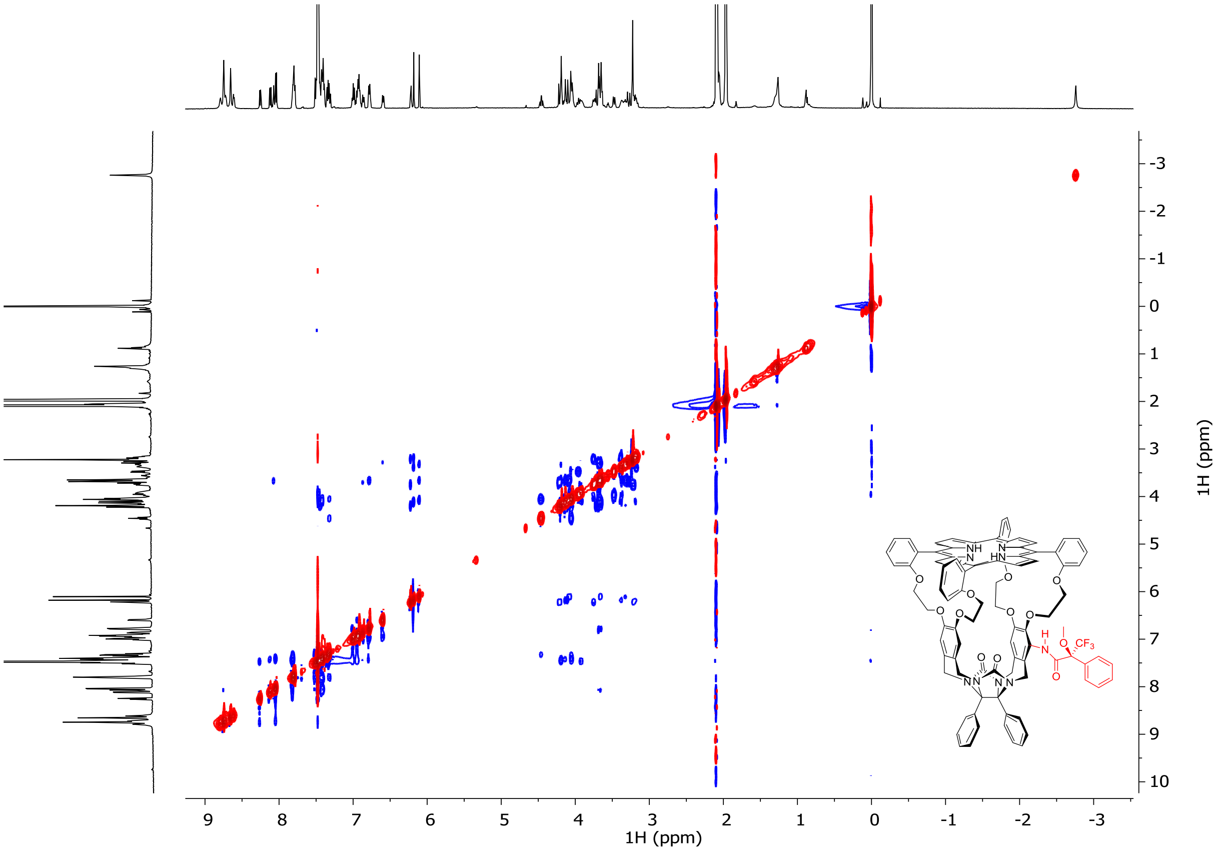

Figure S51. ^{1}H-^{1}H ROESY NMR spectrum (500 MHz, 298 K) of (*R,R**)-$\mathbf{H_25}$ in $CDCl_3$: D_3CCN, (1:1 v/v).

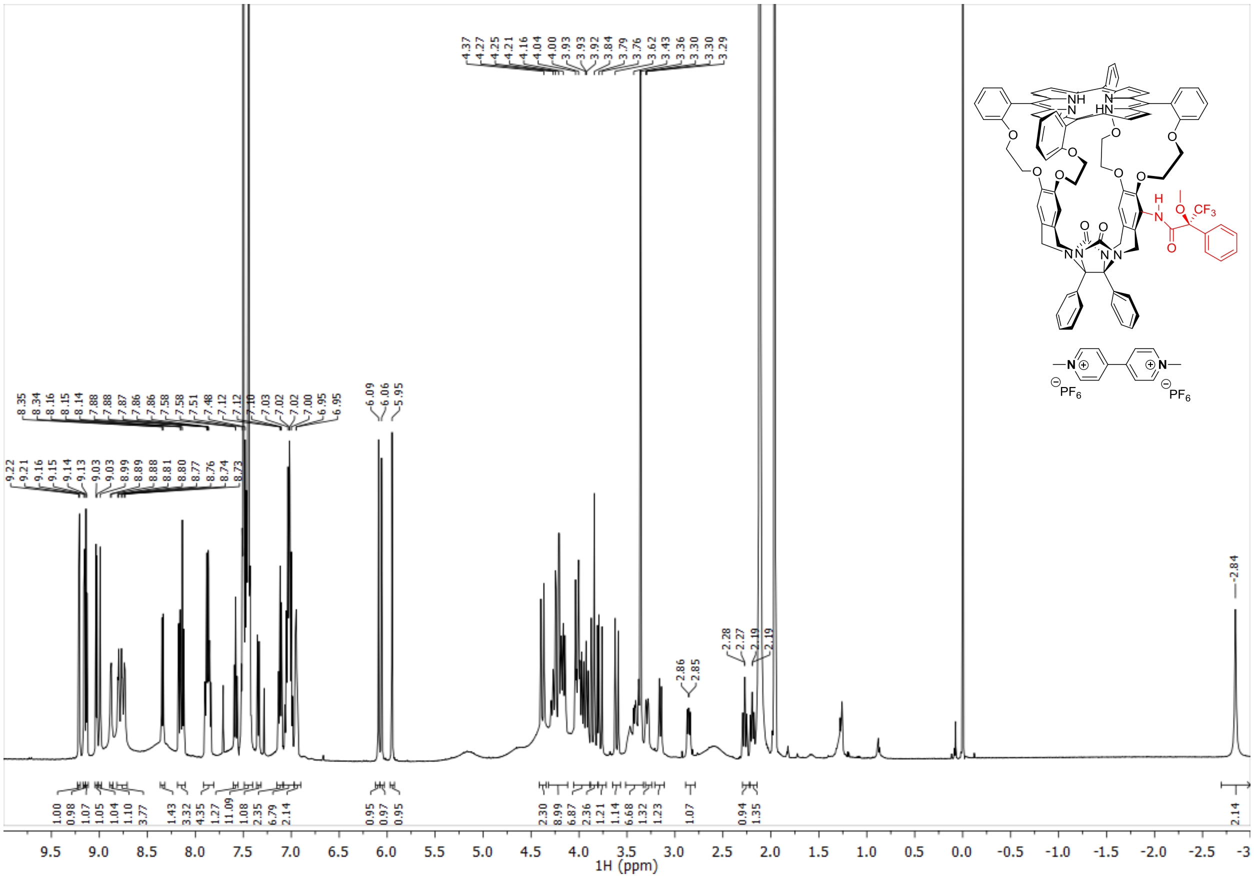

Figure S52. ^{1}H NMR spectrum (500 MHz, 298 K) of the (*R,R**)-**$H_2$5** complex with **V1** in $CDCl_3$: D_3CCN, (1:1 v/v).

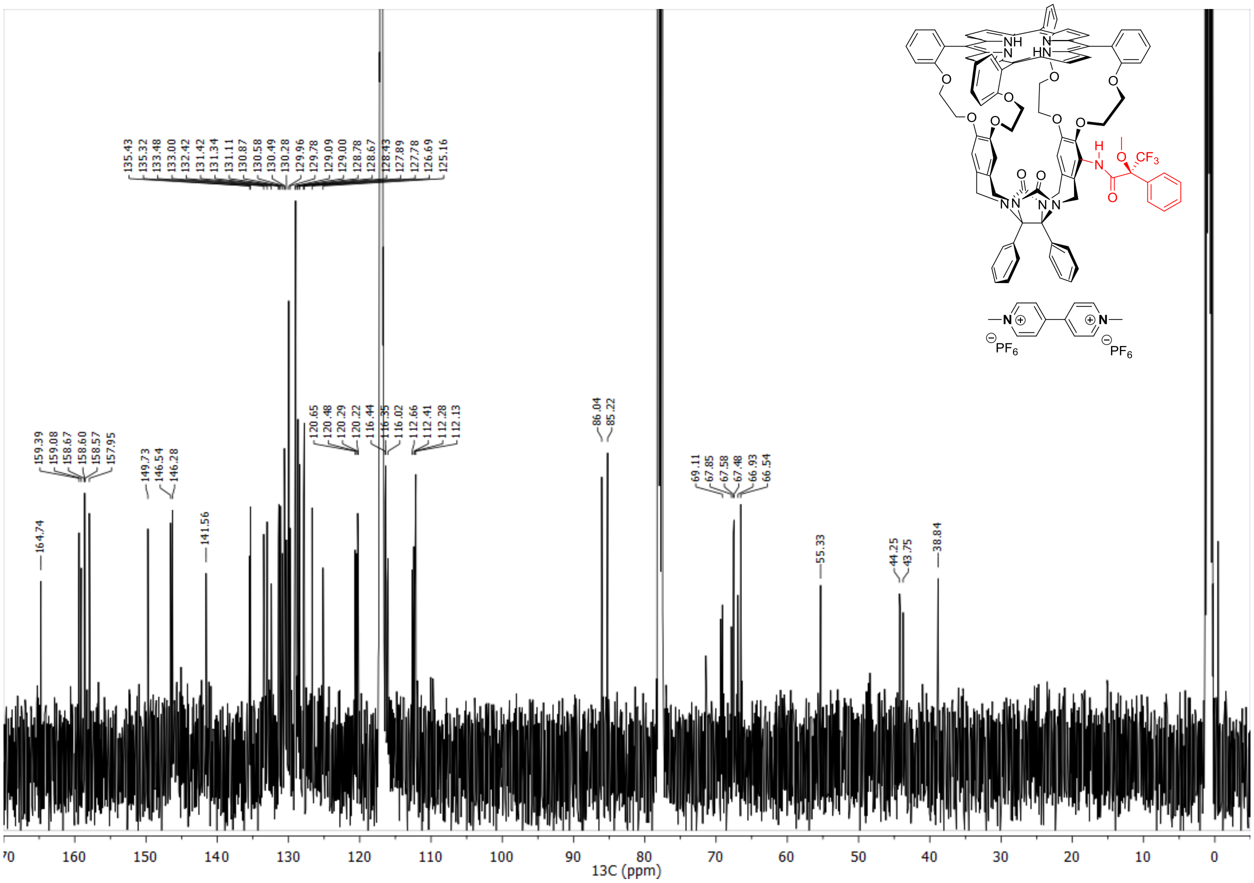

Figure S53. $^{13}C\{^{1}H\}$ NMR spectrum (126 MHz, 298 K) of the (*R,R**)-**$H_2$5** complex with **V1** in $CDCl_3$: D_3CCN, (1:1 v/v).

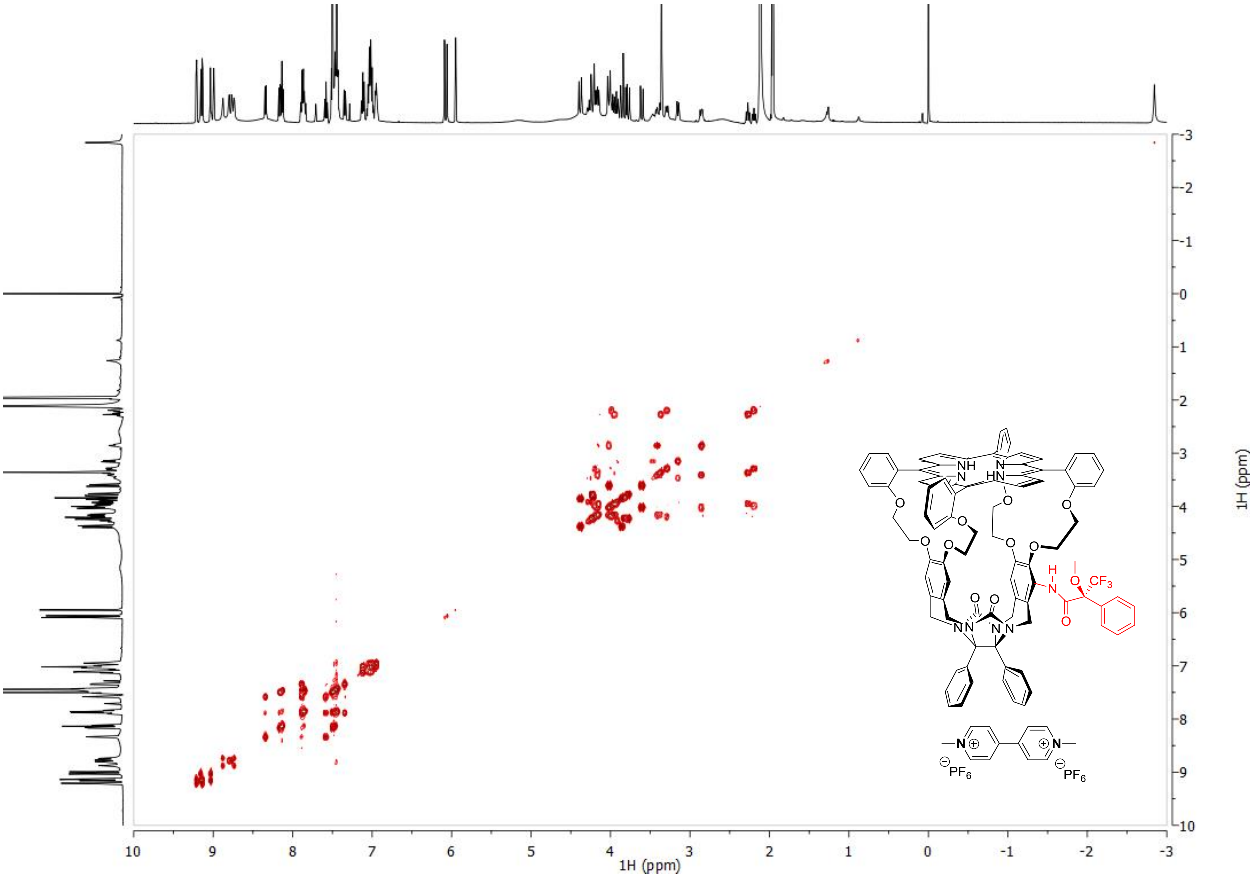

Figure S54. ^{1}H-^{1}H COSY NMR spectrum (500 MHz, 298 K) of the (*R,R**)-**$H_2$5** complex with **V1** in $CDCl_3$: D_3CCN, (1:1 v/v).

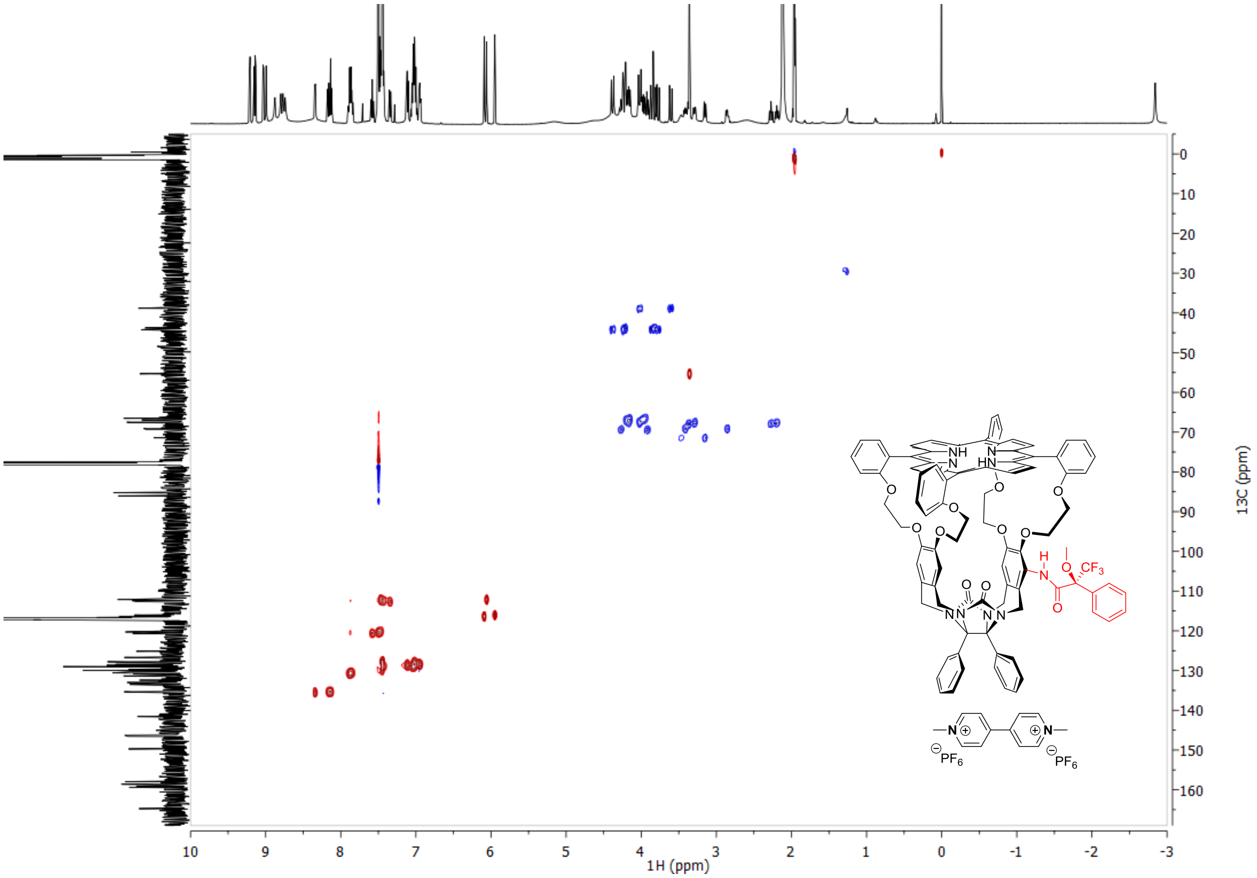

Figure S55. ^{1}H-^{13}C HSQC edited NMR spectrum (500 MHz, 298 K) of the (*R,R**)-**$H_2$5** complex with **V1** in $CDCl_3$: D_3CCN, (1:1 v/v). CH_2 groups are indicated in blue and CH/CH_3 groups in red.

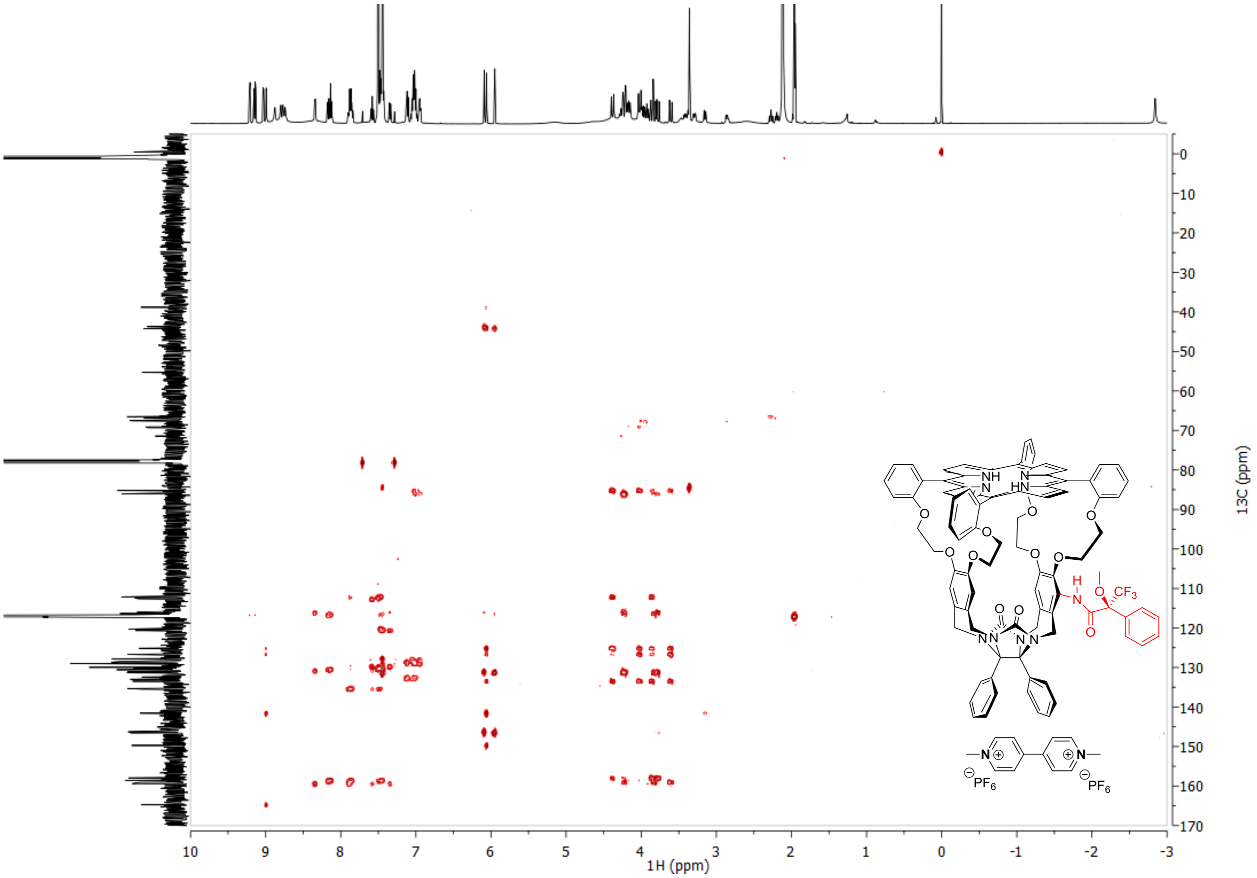

Figure S56. ^{1}H-^{13}C HMBC NMR spectrum (500 MHz, 298 K) of the (*R*,*R**)-**$H_2$5** complex with **V1** in $CDCl_3$: D_3CCN, (1:1 v/v).

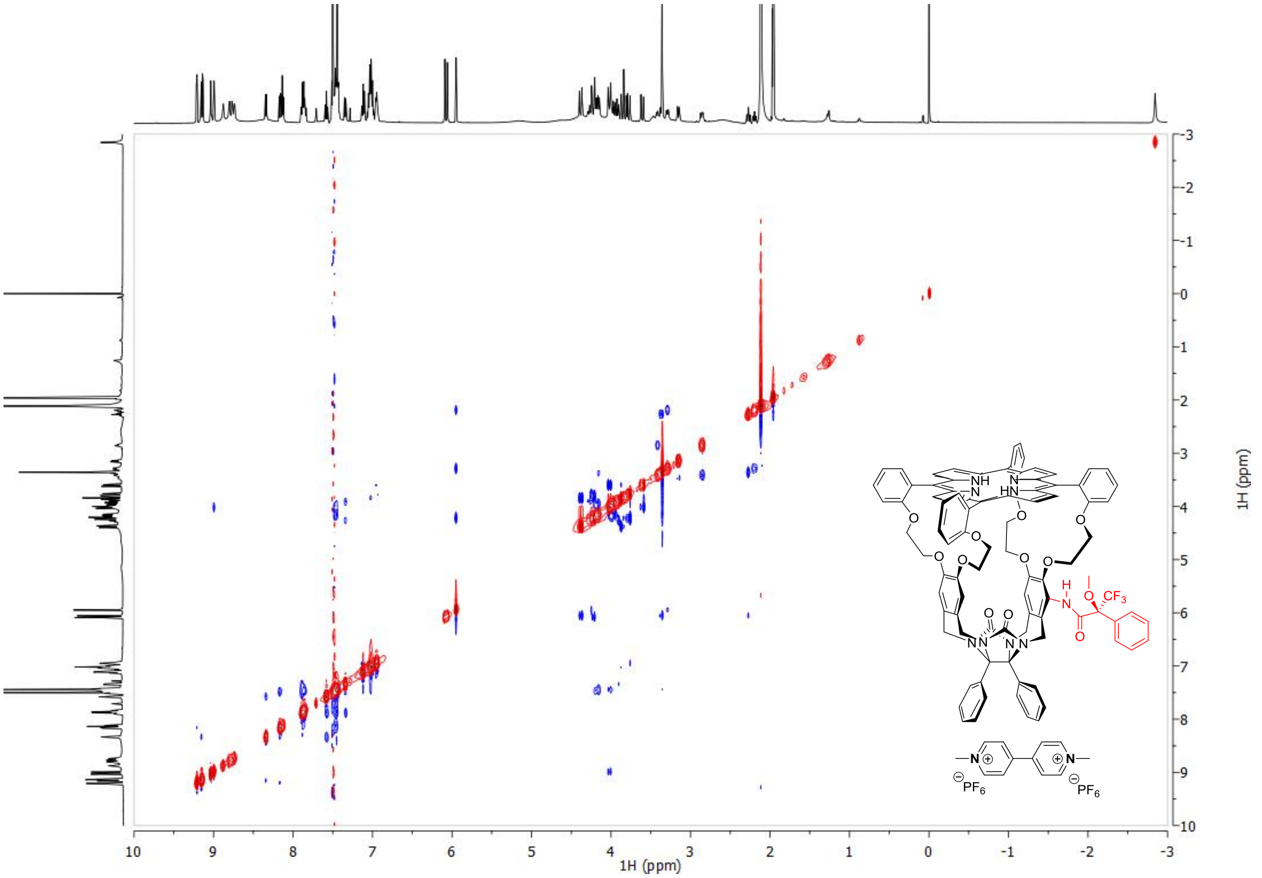

Figure S57. ^{1}H-^{1}H ROESY NMR spectrum (500 MHz, 298 K) of the (*R,R**)-**$H_2$5** complex with **V1** in $CDCl_3$: D_3CCN, (1:1 v/v).

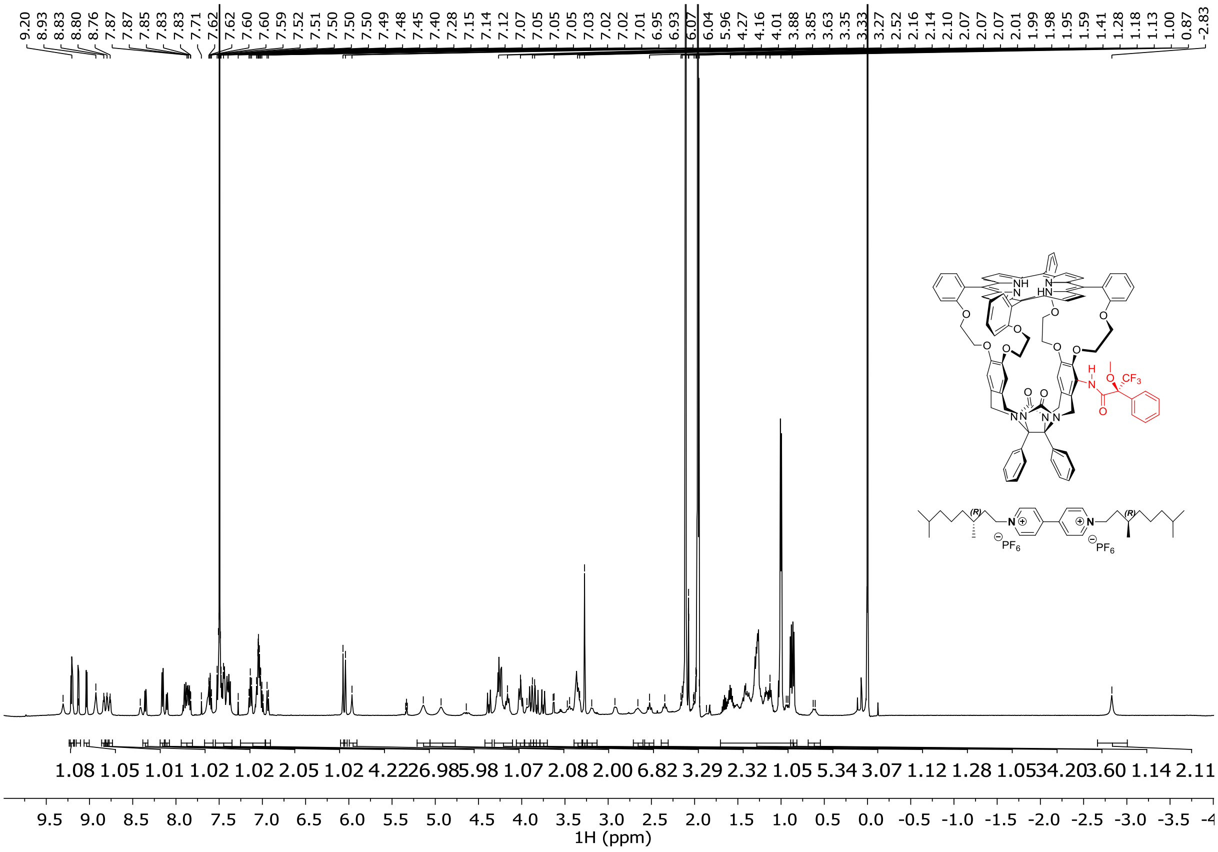

Figure S58. ^{1}H NMR spectrum (500 MHz, 298 K) of (*R*,*R**)-**$H_2$5** complex with ***(R,R)*-V2** in $CDCl_3$: D_3CCN, (1:1 v/v).

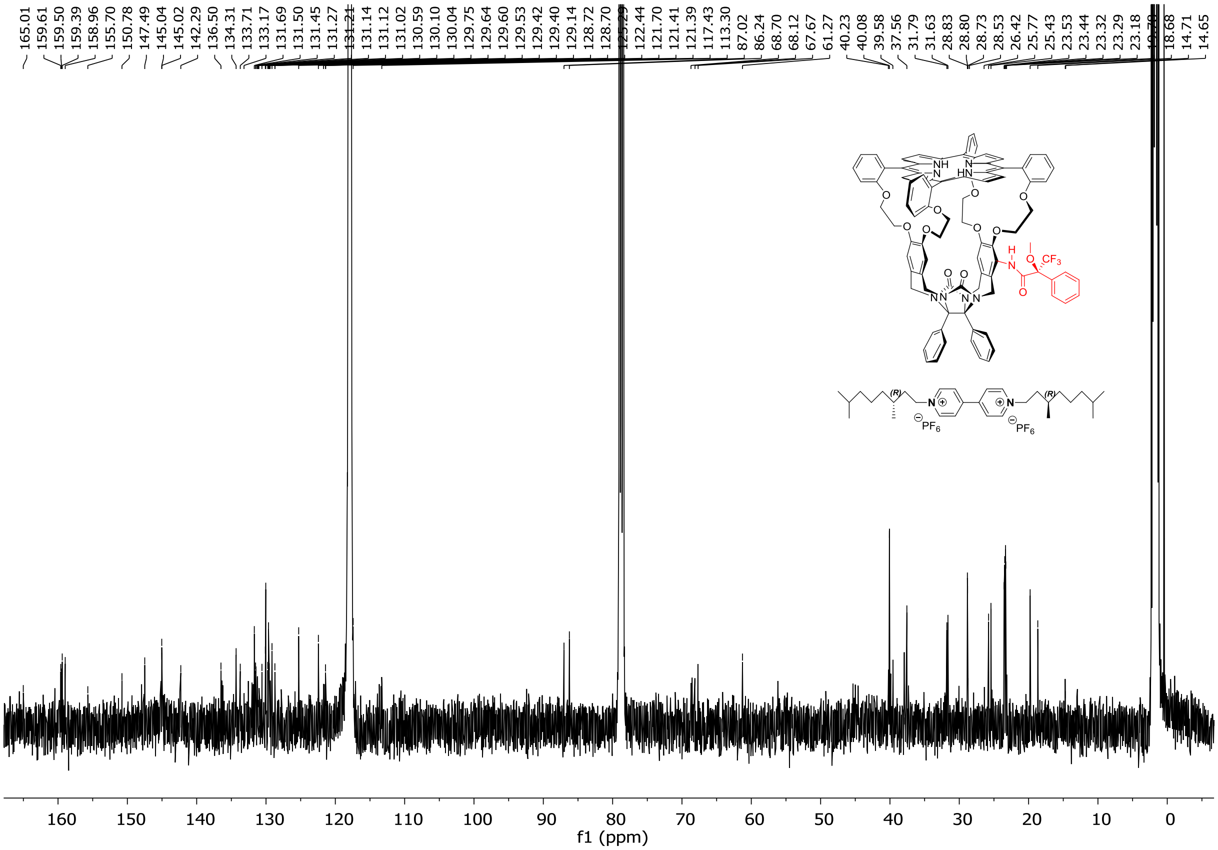

Figure S59. $^{13}C\{^{1}H\}$ NMR spectrum (126 MHz, 298 K) of (*R*,*R**)-**$H_2$5** complex with ***(R,R)*-V2** in $CDCl_3$: D_3CCN, (1:1 v/v).

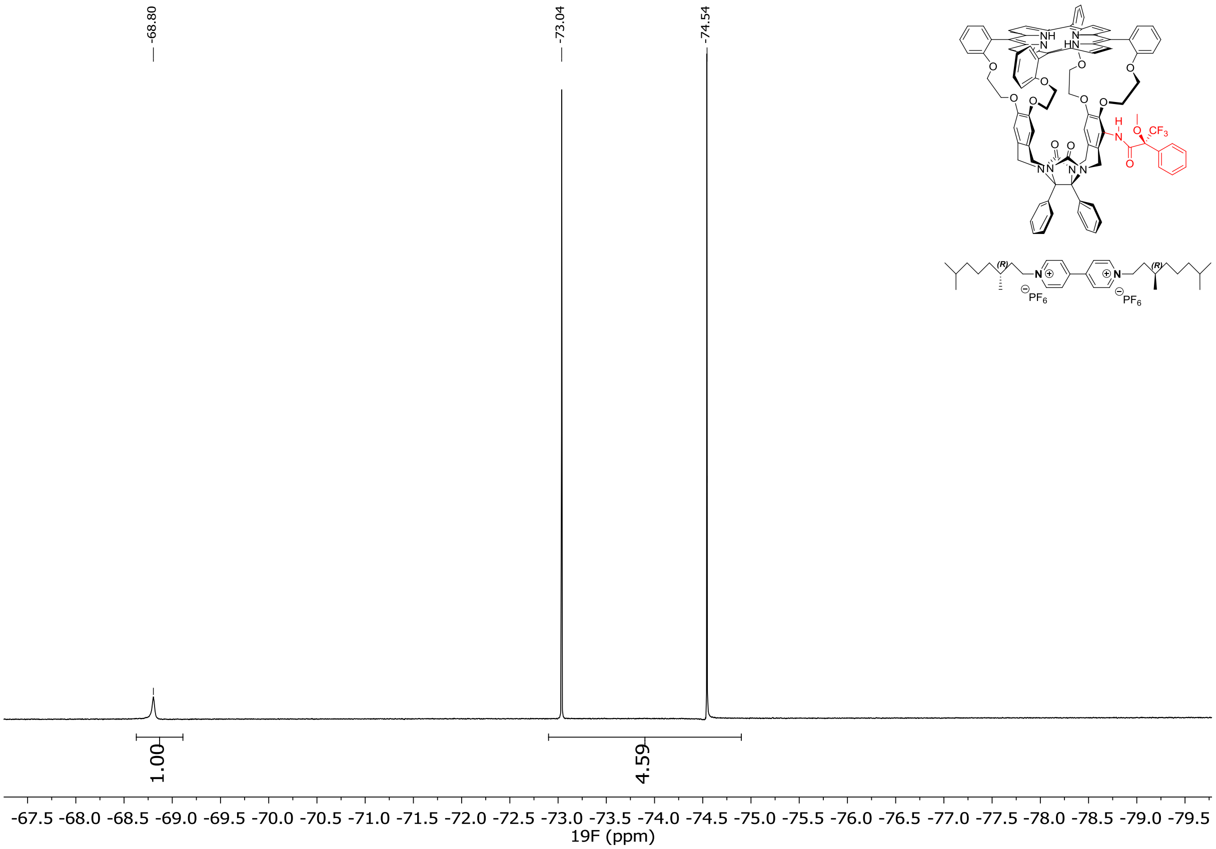

Figure S60. ^{19}F NMR spectrum (471 MHz, 298 K) of (*R,R**)-**$H_2$5** complex with ***(R,R)*-V2** in $CDCl_3$: D_3CCN, (1:1 v/v).

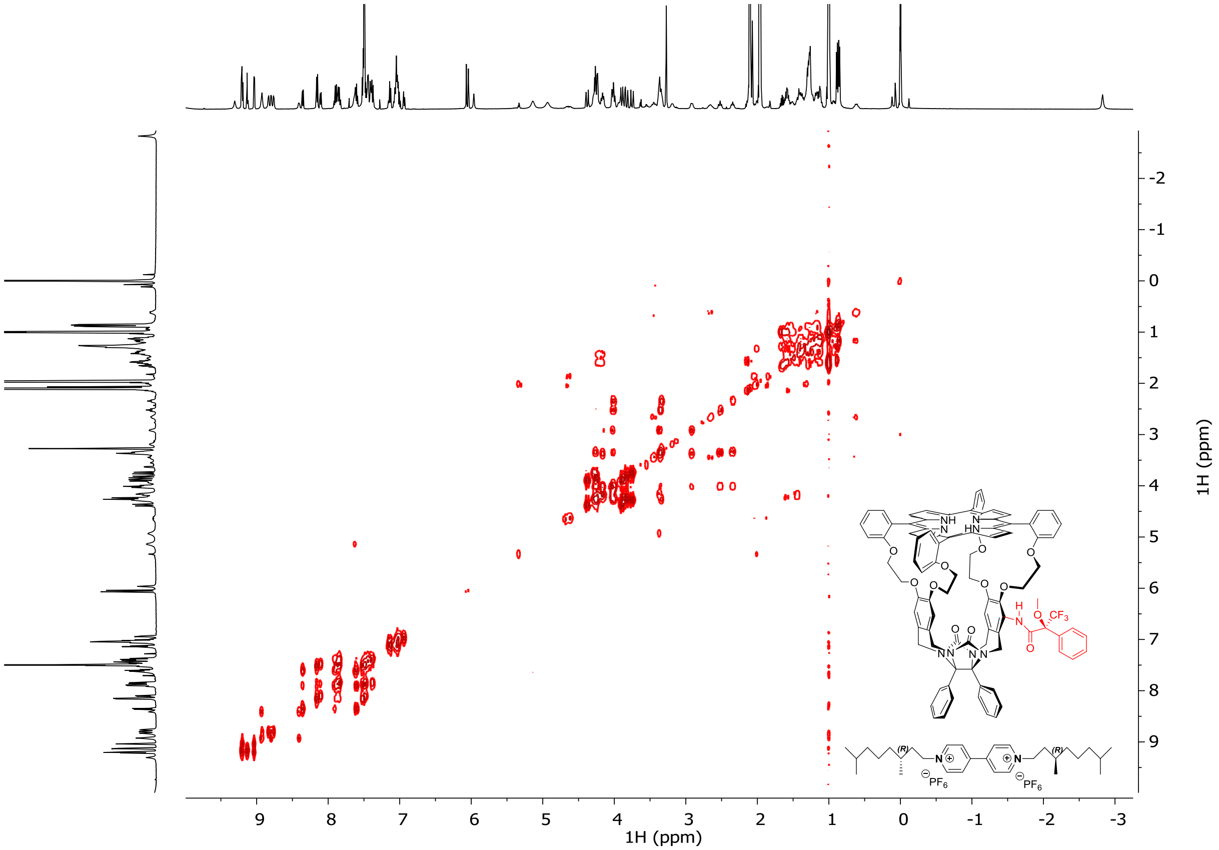

Figure S61. ^{1}H-^{1}H DQF-COSY NMR spectrum (500 MHz, 298 K) of (*R*,*R**)-**$H_2$5** complex with **(*R*,*R*)-V2** in $CDCl_3$: D_3CCN, (1:1 v/v).

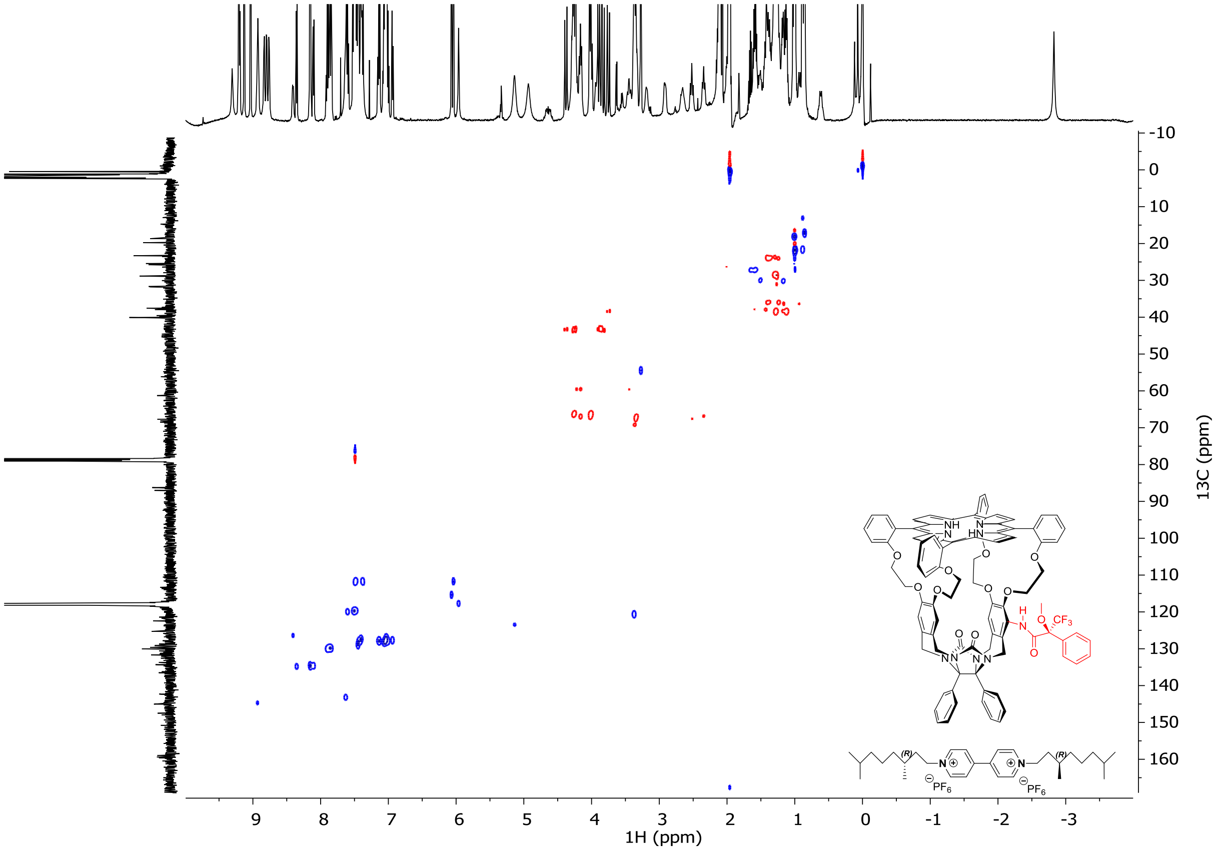

Figure S62. ^{1}H-^{13}C edited HSQC NMR spectrum (500 MHz, 298 K) of (*R*,*R**)-$\mathbf{H_25}$ complex with **(*R*,*R*)-V2** in $CDCl_3$: D_3CCN, (1:1 v/v). CH_2 groups are indicated in blue and CH/CH_3 groups in red.

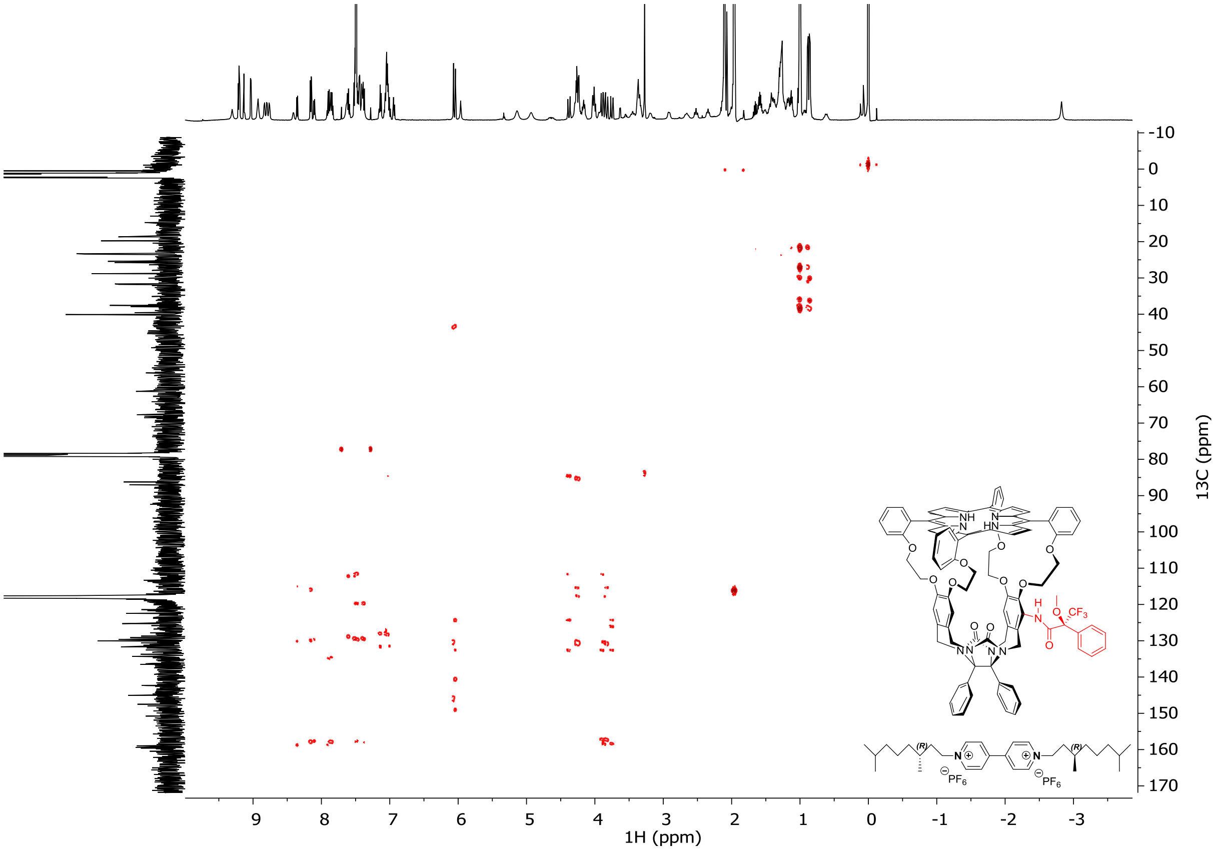

Figure S63. ^{1}H-^{13}C HMBC NMR spectrum (500 MHz, 298 K) of (*R*,*R**)-**$H_2$5** complex with **(*R*,*R*)-V2** in $CDCl_3$: D_3CCN, (1:1 v/v).

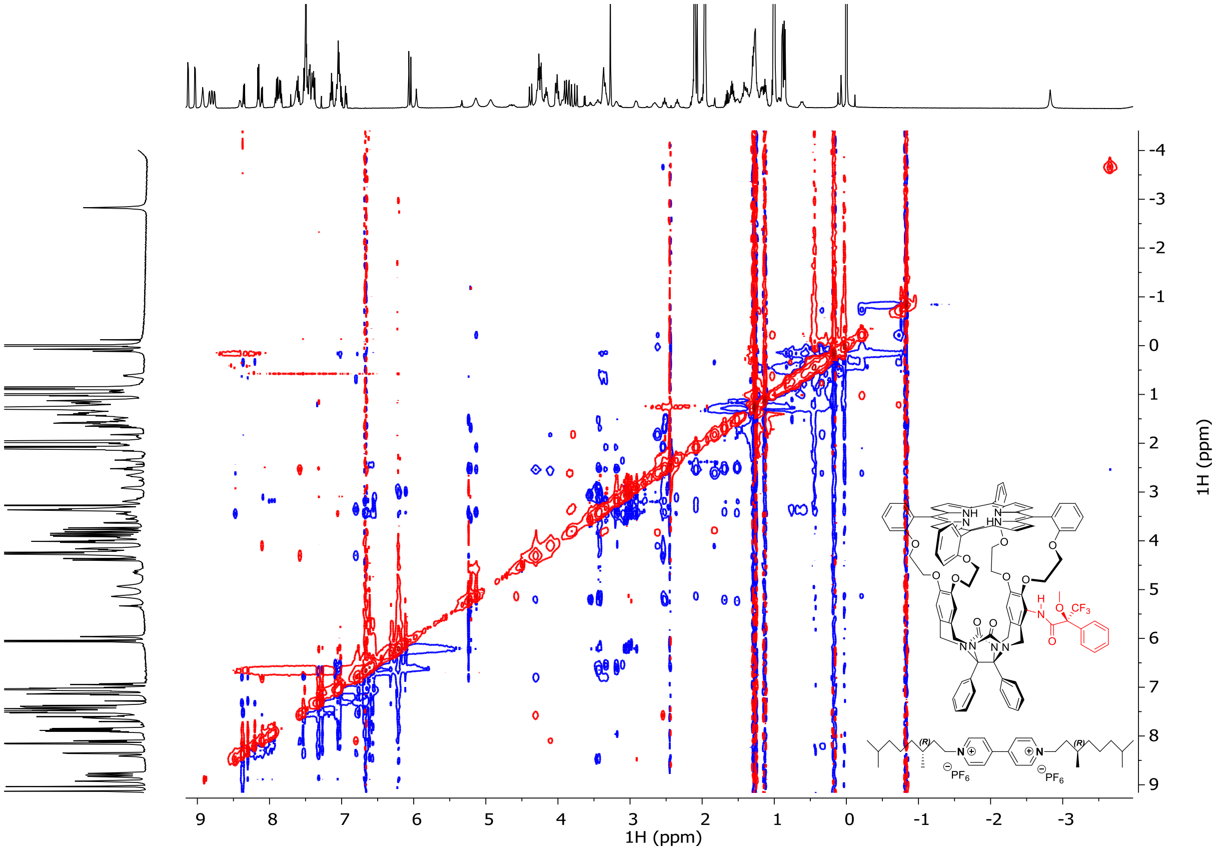

Figure S64. ^{1}H-^{1}H ROESY NMR spectrum (500 MHz, 298 K) of (*R,R**)-**$H_2$5** complex with **(*R*,*R*)-V2** in $CDCl_3$: D_3CCN, (1:1 v/v).

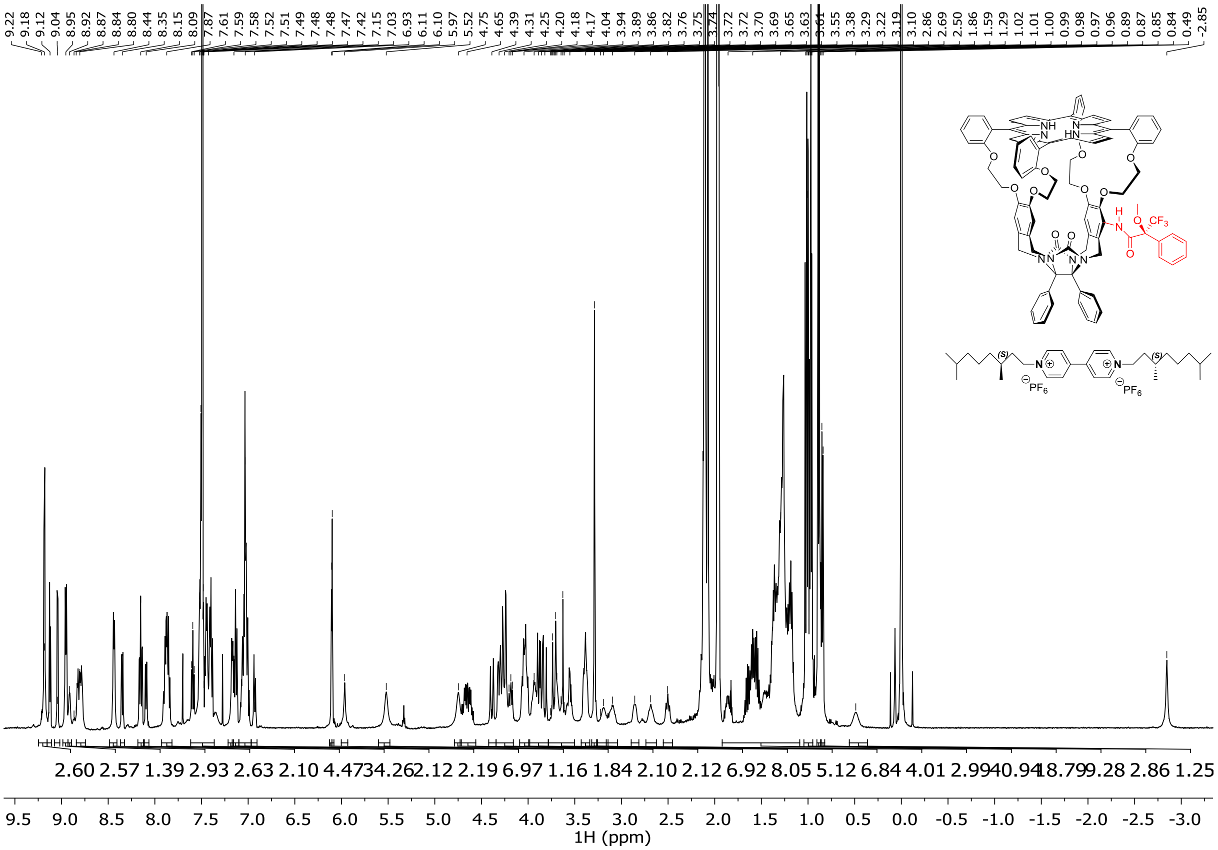

Figure S65. ^{1}H NMR spectrum (500 MHz, 298 K) of (*R,R**)-**$H_2$5** complex with **(*S*,*S*)-V2** in $CDCl_3$: D_3CCN, (1:1 v/v).

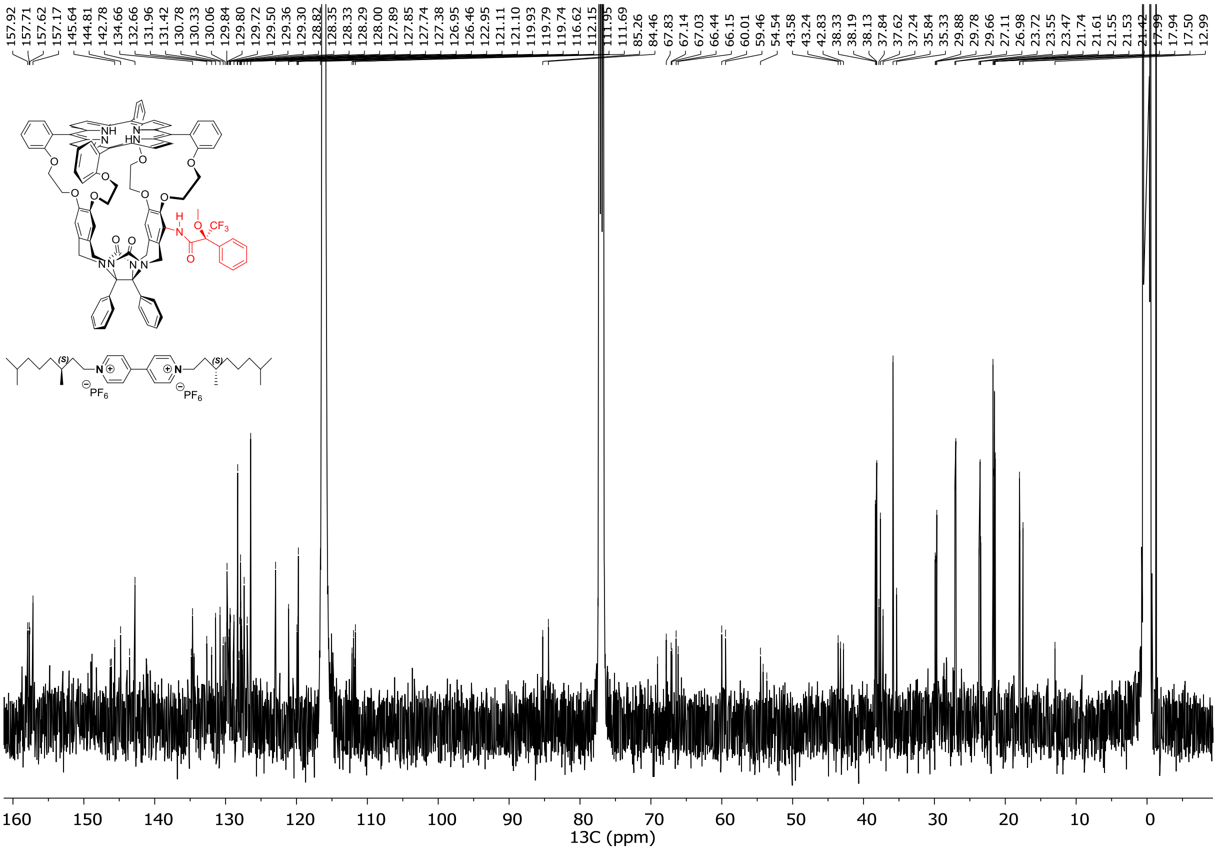

Figure S66. $^{13}C\{^{1}H\}$ NMR spectrum (126 MHz, 298 K) of (*R,R**)-**$H_2$5** complex with **(*S*,*S*)-V2** in $CDCl_3$: D_3CCN, (1:1 v/v).

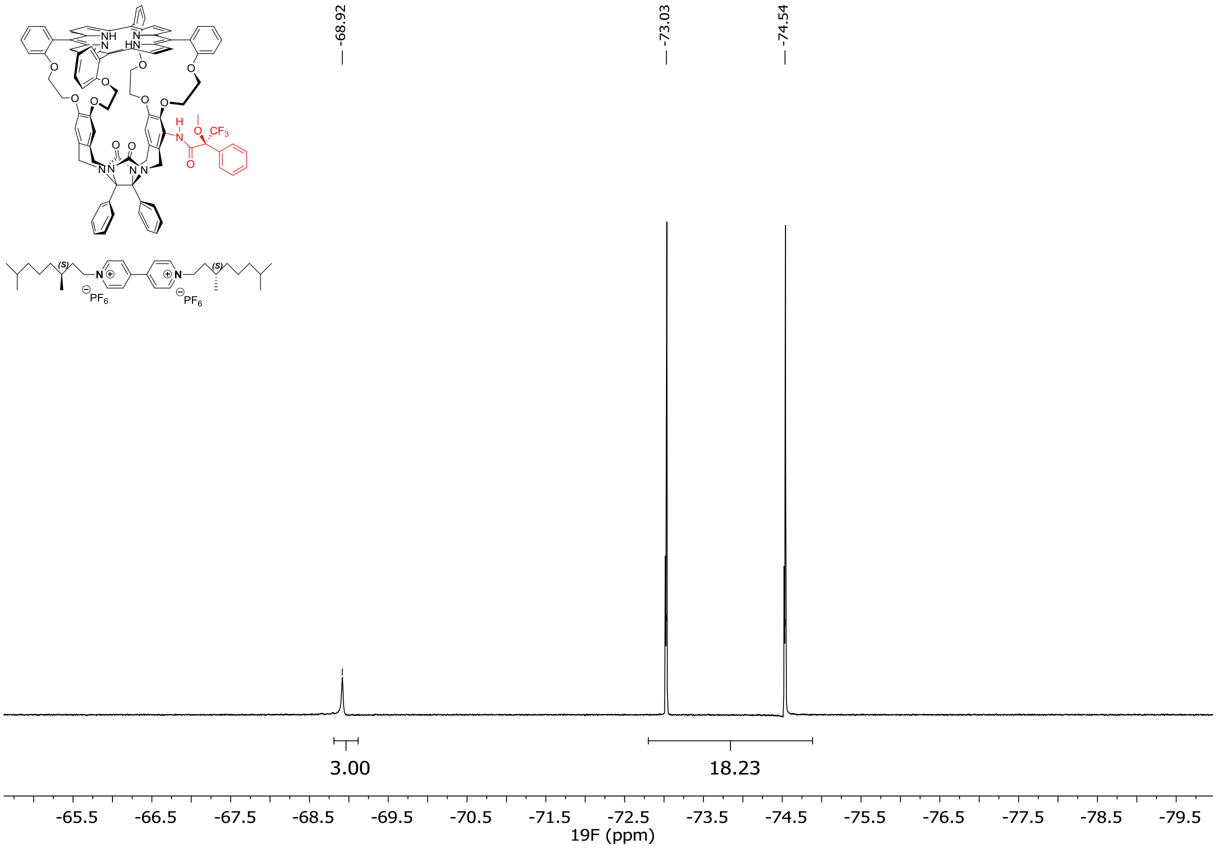

Figure S67. ^{19}F NMR spectrum (471 MHz, 298 K) of (*R*,*R**)-**$H_2$5** complex with **(*S*,*S*)-V2** in $CDCl_3$: D_3CCN, (1:1 v/v).

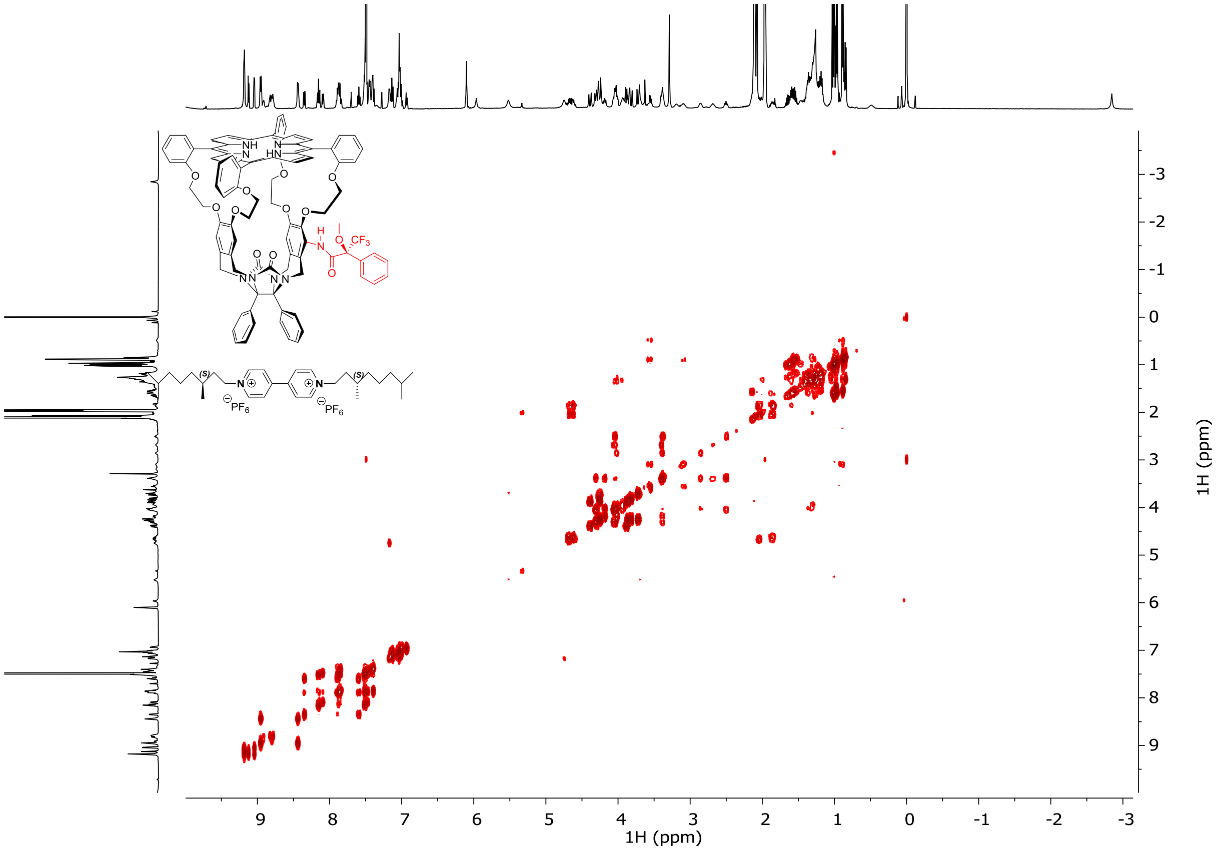

Figure S68. ^{1}H-^{1}H DQF-COSY NMR spectrum (500 MHz, 298 K) of (*R*,*R**)-**$H_2$5** complex with **(*S*,*S*)-V2** in $CDCl_3$: D_3CCN, (1:1 v/v).

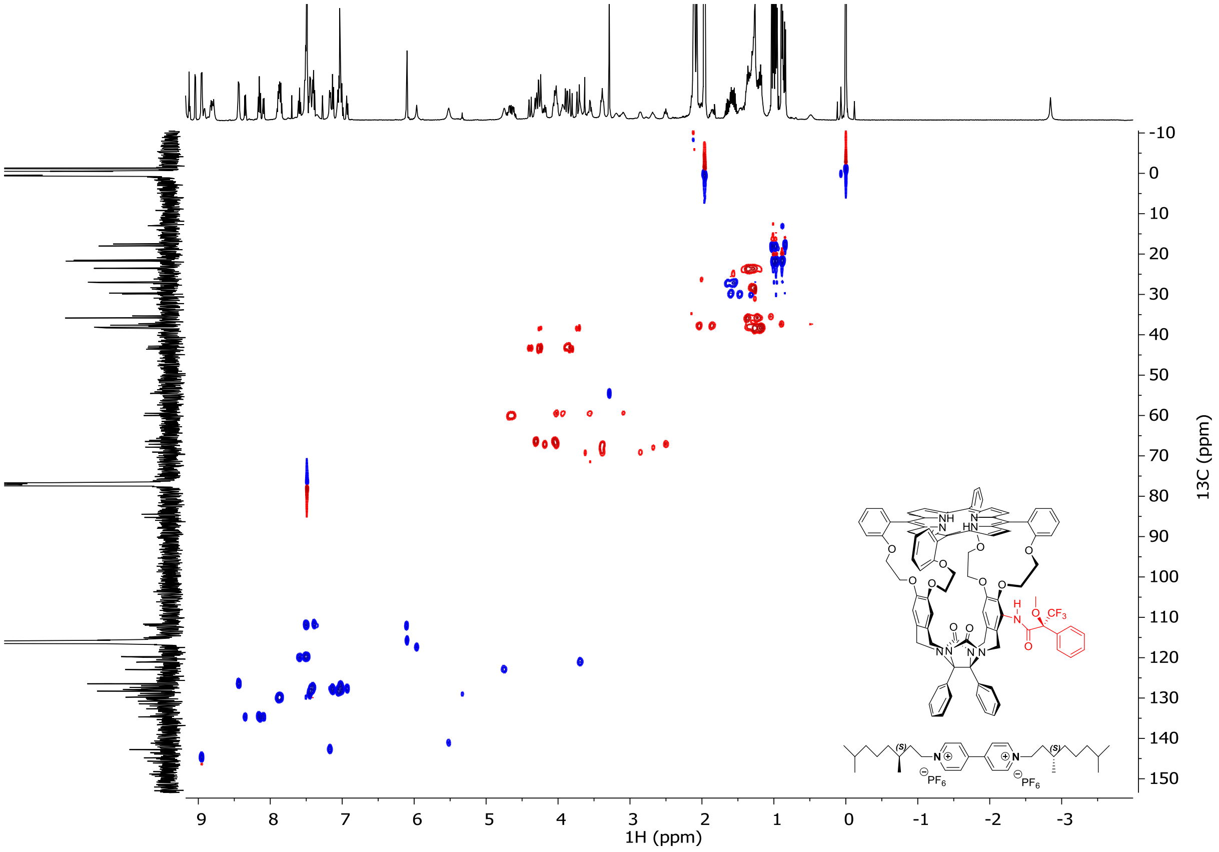

Figure S69. ^{1}H-^{13}C edited HSQC NMR spectrum (500 MHz, 298 K) of (*R,R**)-**$H_2$5** complex with ***(S,S)*-V2** in $CDCl_3$: D_3CCN, (1:1 v/v). CH_2 groups are indicated in blue and CH/CH_3 groups in red.

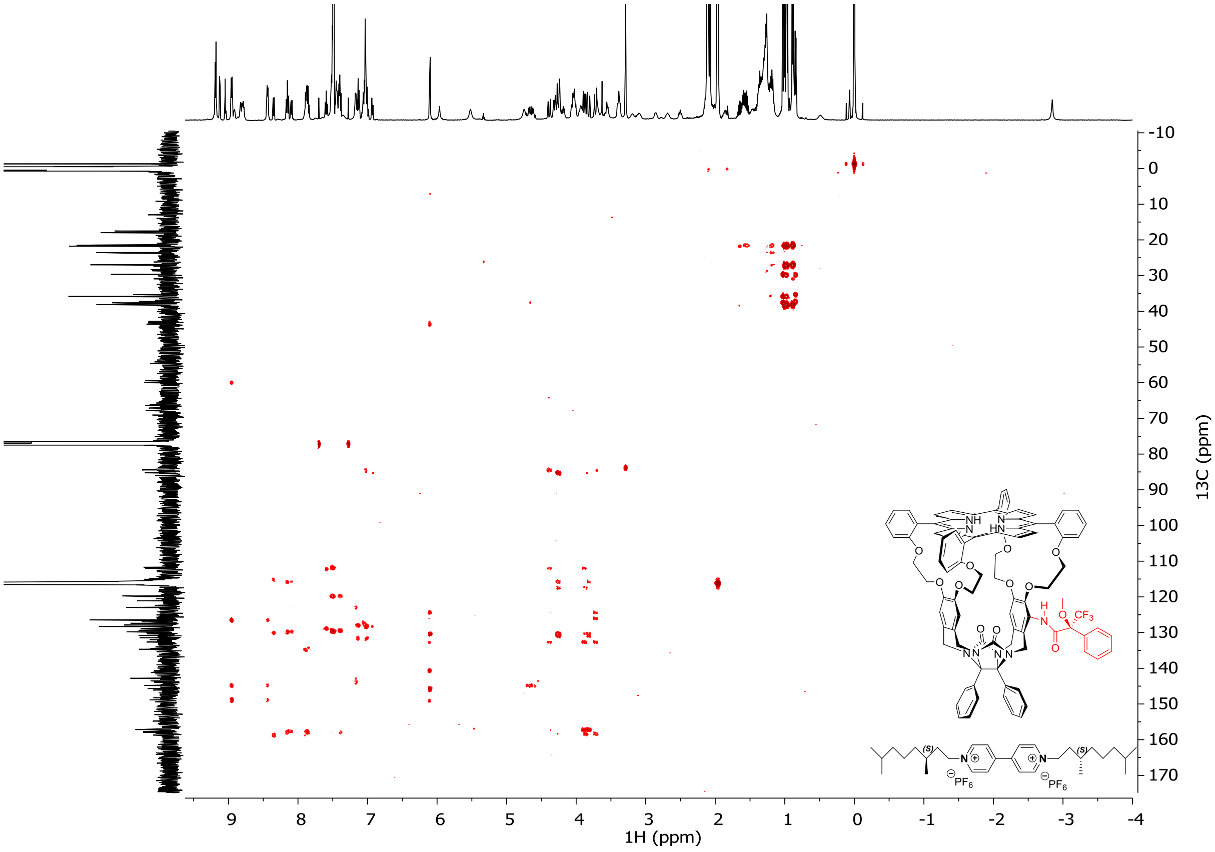

Figure S70. ^{1}H-^{13}C HMBC NMR spectrum (500 MHz, 298 K) of (*R*,*R**)-**$H_2$5** complex with **(*S*,*S*)-V2** in $CDCl_3$: D_3CCN, (1:1 v/v).

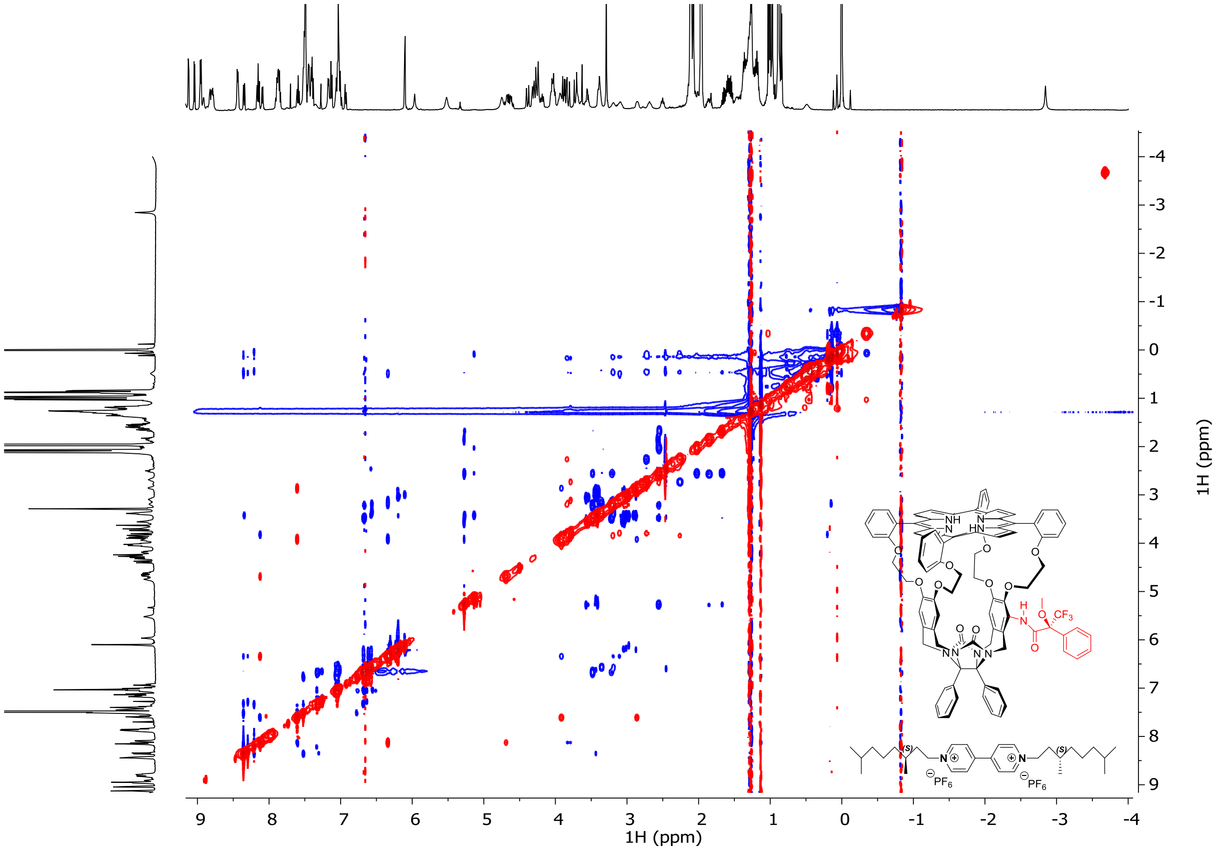

Figure S71. ^{1}H-^{1}H ROESY NMR spectrum (500 MHz, 298 K) of (*R*,*R**)-**H$_2$5** complex with **(*S*,*S*)-V2** in $CDCl_3$: D_3CCN, (1:1 v/v).

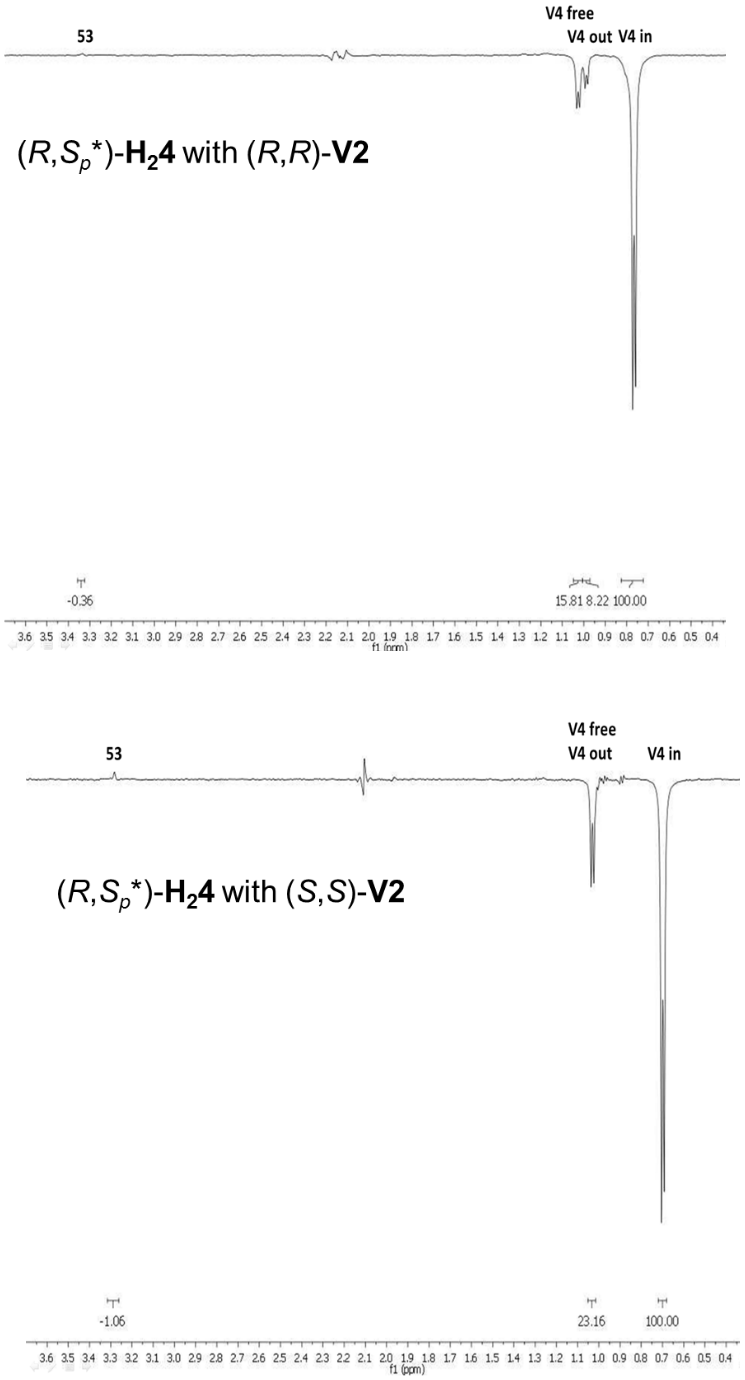

Figure S72. 1D-ROESY experiment in which protons *V4* of guests (*R*,*R*)-**V2** and (*S*,*S*)-**V2** bound inside host (*R*,S_p*)-$\mathbf{H_24}$ are excited. Most of the magnetization is transferred

to the *V4* of the free guest due to chemical exchange. However, a small amount is transferred to the methoxy proton *53* of the Mosher substituent in (R,S_p*)-**$H_2$4**. In the case of (*R*,*R*)-**V2** the magnetization transfer is 0.36% and in the case of (*S*,*S*)-**V2** 1.06%. V4in are the methyl protons of the bound guest closest to the Mosher substituent and V4out the methyl protons of the bound guest farthest away from this substituent. V4free are the methyl protons of the unbound guest.

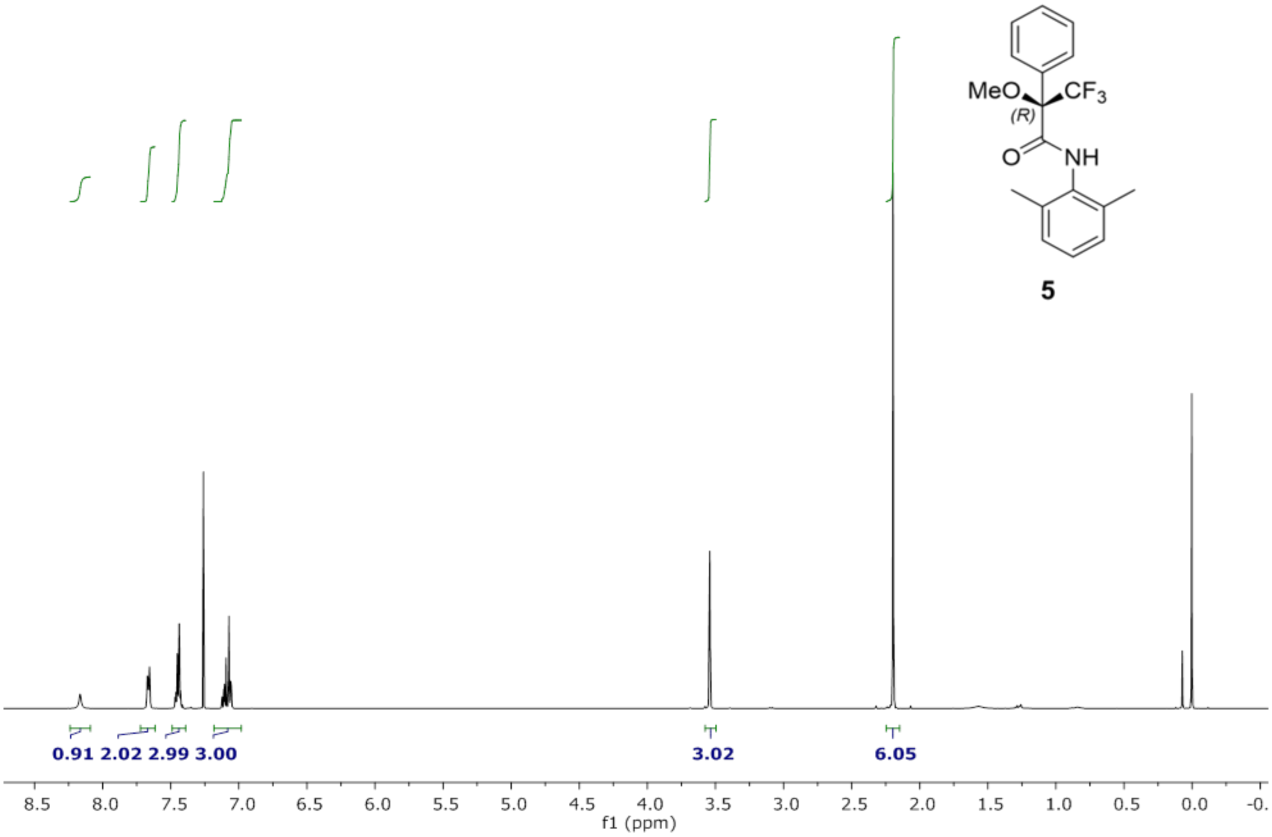

Figure S73. ^{1}HNMR spectrum of compound **5** in chloroform-*d*.

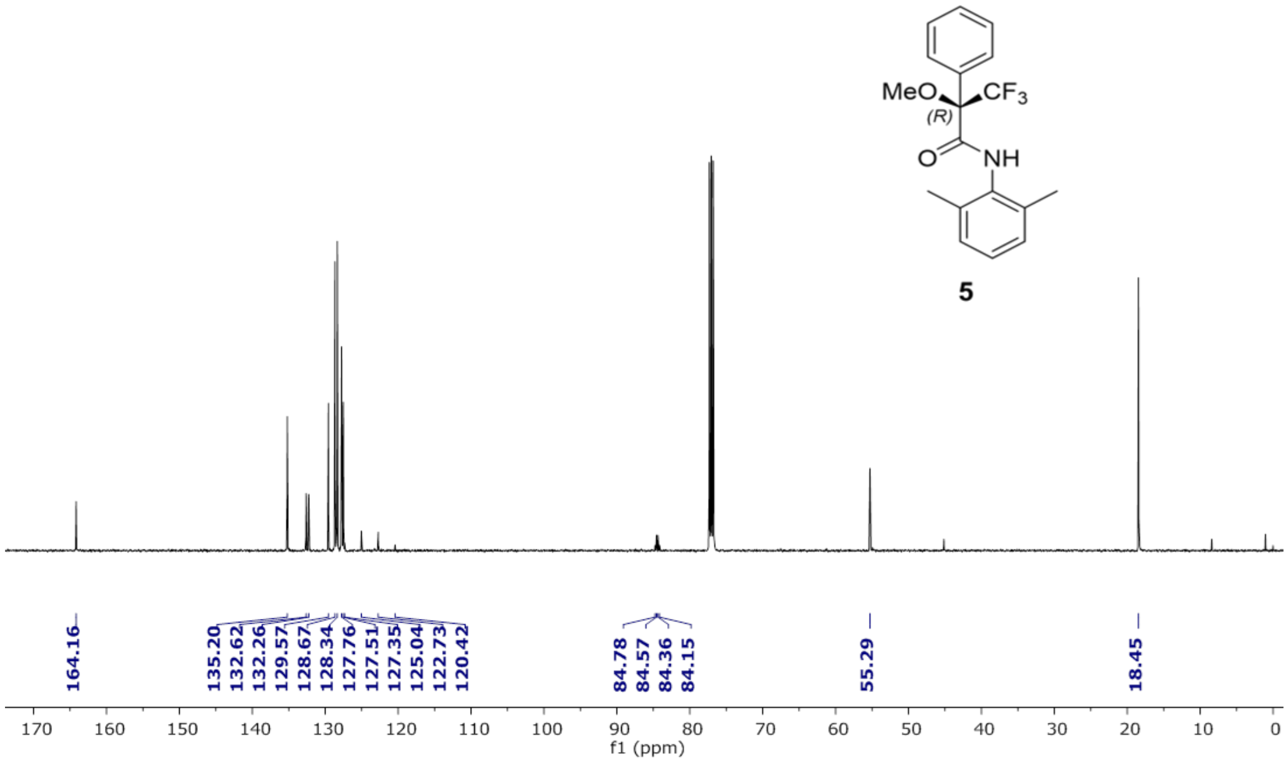

Figure S74. ^{13}C NMR (126 MHz, chloroform-*d*) of compound **5**.

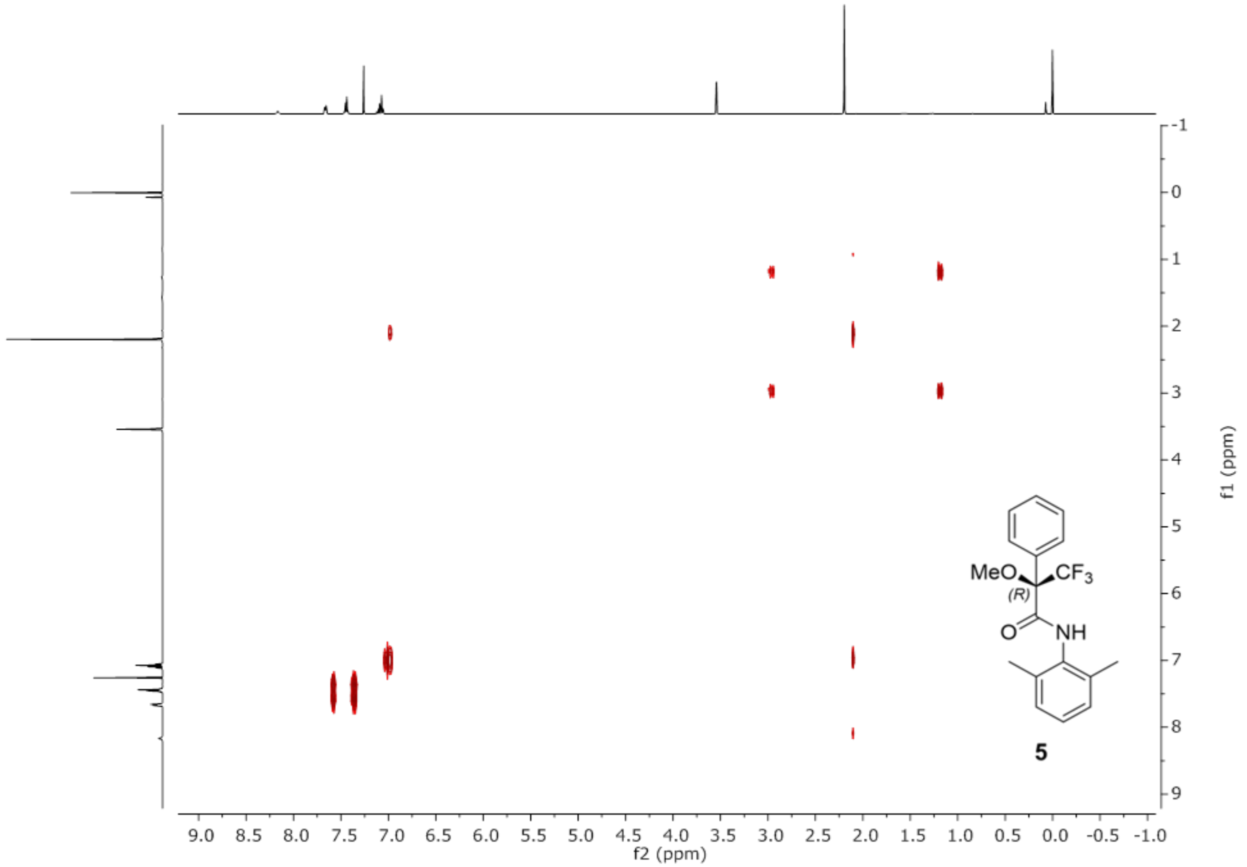

Figure S75. ^{1}H-^{1}H DQF-COSY NMR spectrum (500 MHz, 298 K) of compound **5** in chloroform-*d*.

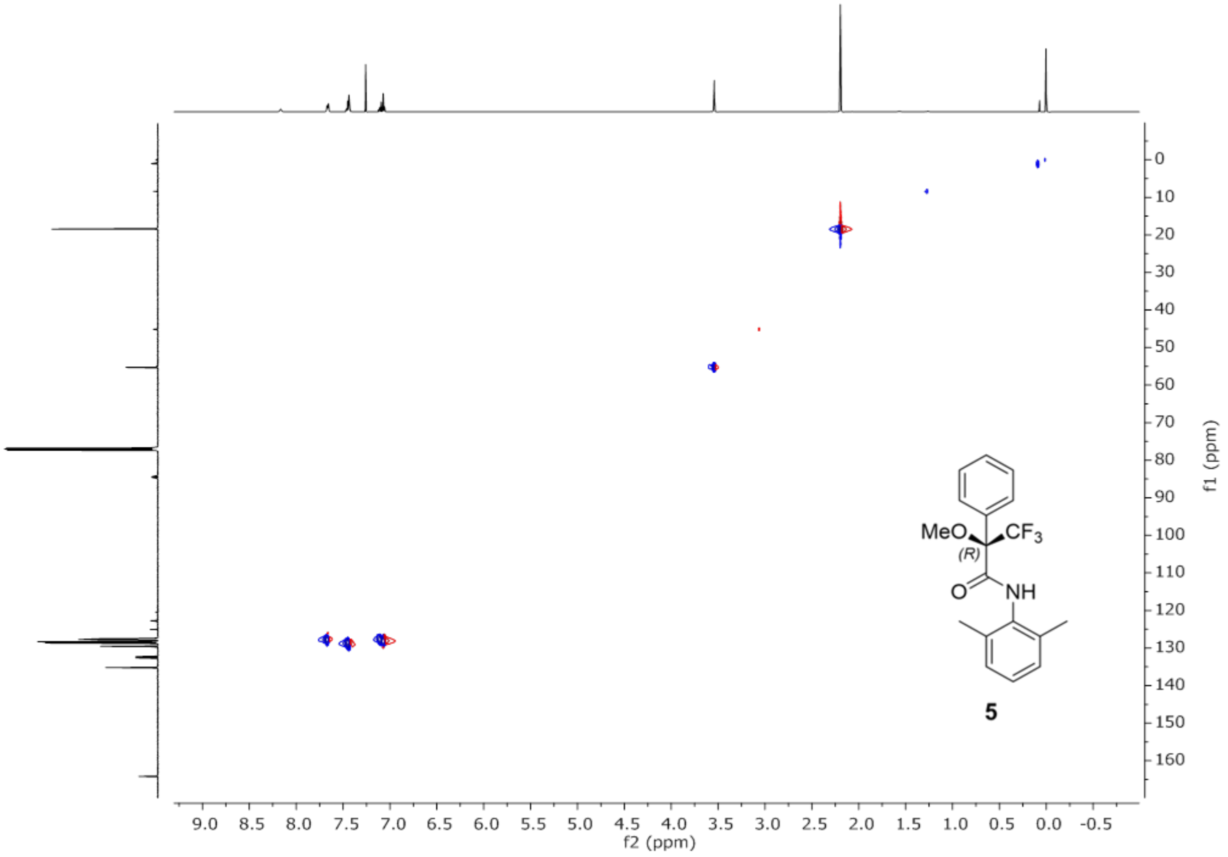

Figure S76. ^{1}H-^{13}C edited HSQC NMR spectrum (500 MHz, 298 K) of compound **5** in chloroform-*d*. CH_2 groups are indicated in red and CH/CH_3 groups in blue.

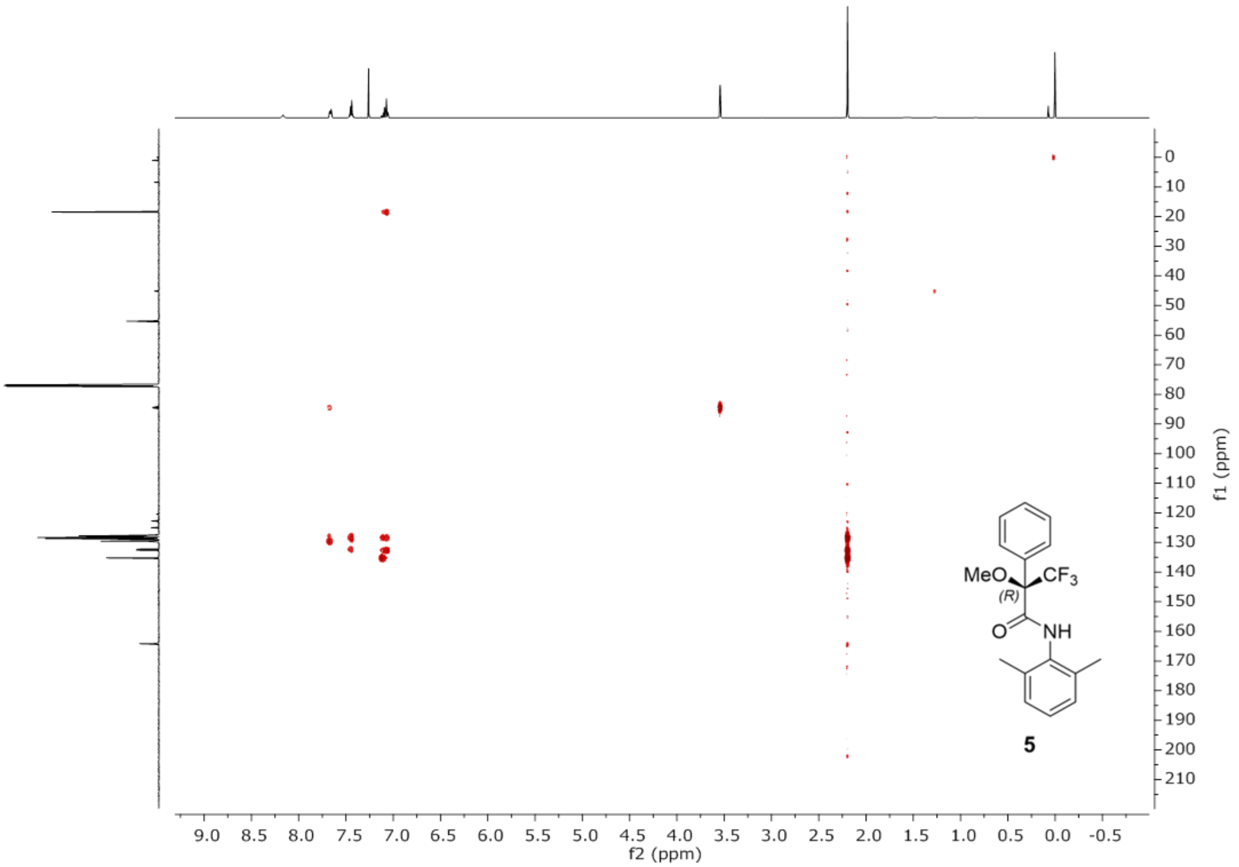

Figure S77. ^{1}H-^{13}C HMBC NMR spectrum (500 MHz, 298 K) of compound **5** in chloroform-*d*.
